# Supplementary material for: Antiviral RNAi defective 4 (AVI4) encodes a lysophospholipid acyltransferase coopting with AVI2 and ATG8a to promote antiviral immunity in Arabidopsis
Source: Plant Commun. 2026 Apr 14;7(9):101868. doi: 10.1016/j.xplc.2026.101868 (PMC13589543; doi:10.1016/j.xplc.2026.101868)
Supplement: Supplementary file 1 [file mmc1.pdf]

Plant Communications, Volume *7*

Supplemental information

Antiviral RNAi defective 4 (AVI4) encodes a lysophospholipid acyltransferase coopting with AVI2 and ATG8a to promote antiviral immunity in *Arabidopsis*

Wenkai Yan, Yaogui Zhou, Bei Jia, Jiangshuai Niu, Shihuai Jin, Zhuoning Zhao, and Zhongxin Guo

Supplemental Figures and Table:

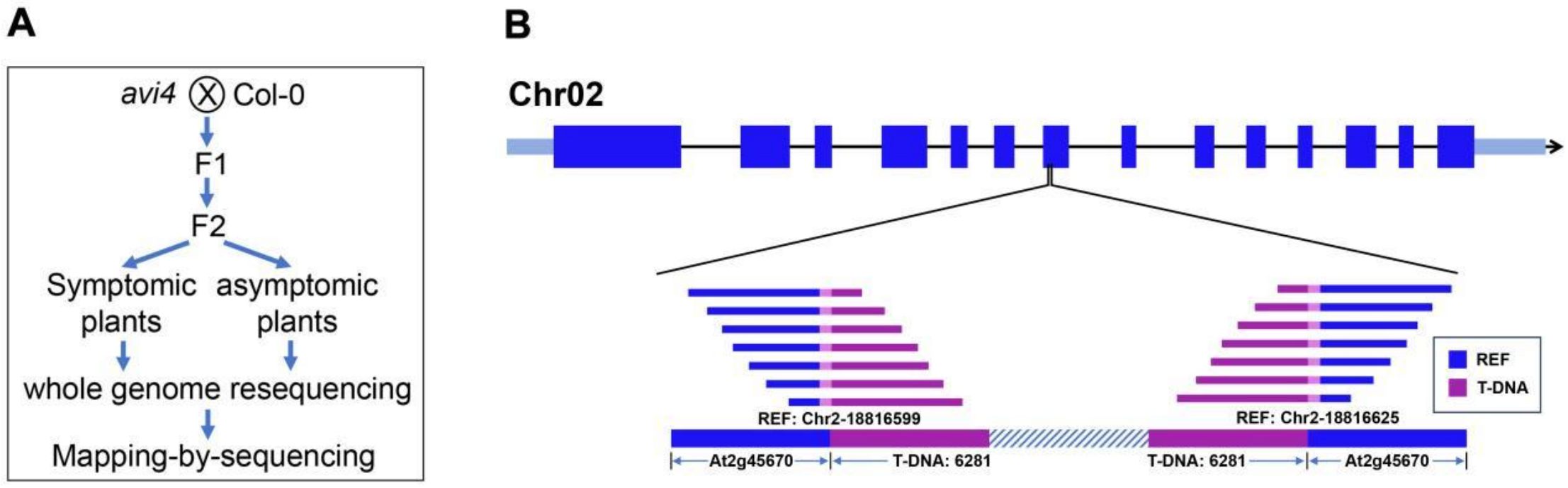

Figure S1. Identification of *avi4* T-DNA position. (A) The flowchart for identifying the causal mutation in *avi4* by whole-genome resequencing. (B) Schematic diagram showing reads flanking T-DNA borders and the position of T-DNA insertion on Chromosome 2 in *avi4* mutant.

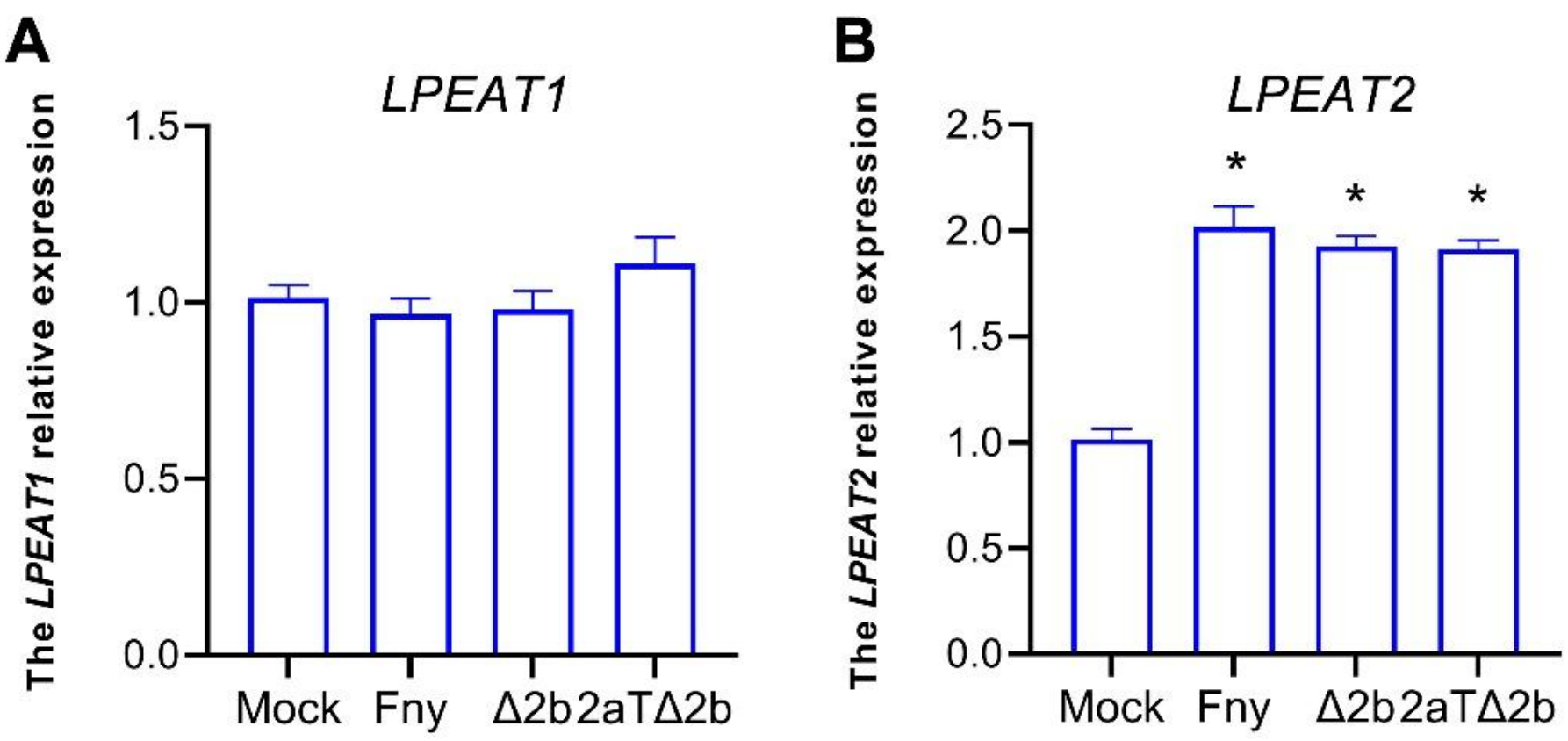

Figure S2. The expression of LPEAT2 is up-regulated in response to CMV infection. (A) and (B) Quantitative analysis of *LPEAT1* (A) and *LPEAT2* (B) mRNA accumulation in Arabidopsis, with or without viral infection. Col-0 plants were inoculated with buffer for mock treatment, or Fny-CMV, CMV-Δ2b, CMV-2aTΔ2b. Total RNA was extracted from systemic leaves 14 days post-infection, and the relative expression levels of the *LPEAT1* and *LPEAT2* genes were determined using RT-qPCR. *A. thaliana PP2A* mRNA served as an internal control. Error bars represent the standard deviation (SD) from three independent experiments, *$p < 0.05$ (Student's t-test).

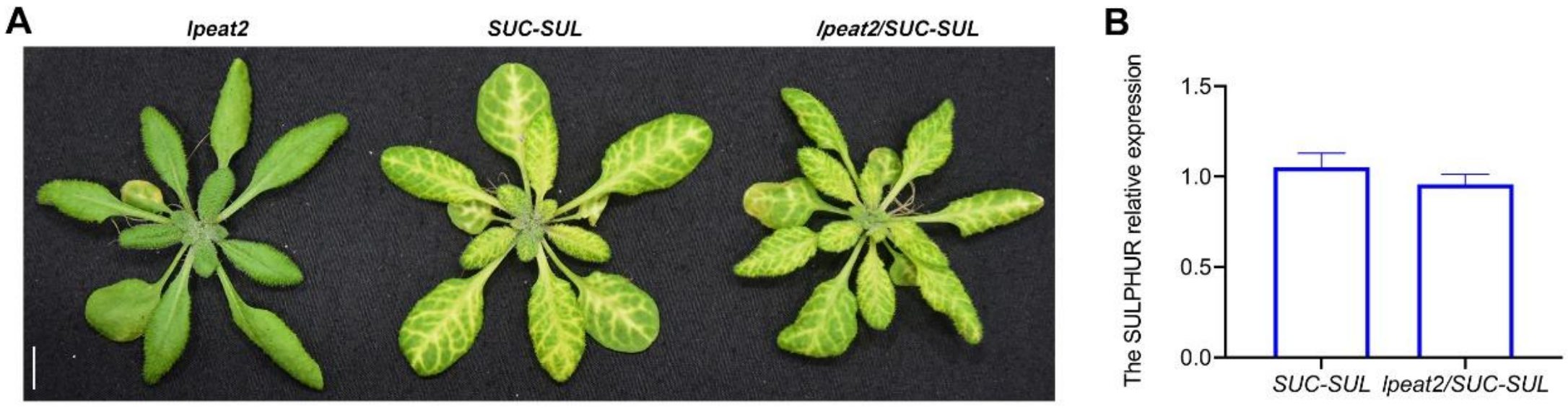

Figure S3. The silencing of *SULPHUR* (*SUL*) in SUC-SUL plants is not affected by LPAET2 mutation. (A) phenotype of *lpeat2*, *SUC-SUL* and *lpeat2 SUC-SUL* plants photographed approximately one month after germination. *SUC-SUL* and *lpeat2 SUC-SUL* showed the similar yellow vein phenotype caused by *SULPHUR* silencing. Bar= 1 cm. (B) RT-qPCR analysis showed that the relative expression level of *SULPHUR* gene in *SUC-SUL* and *lpeat2 SUC-SUL* plants did not show clear difference, *PP2A* was used as internal control, Error bars indicate standard deviation (SD).

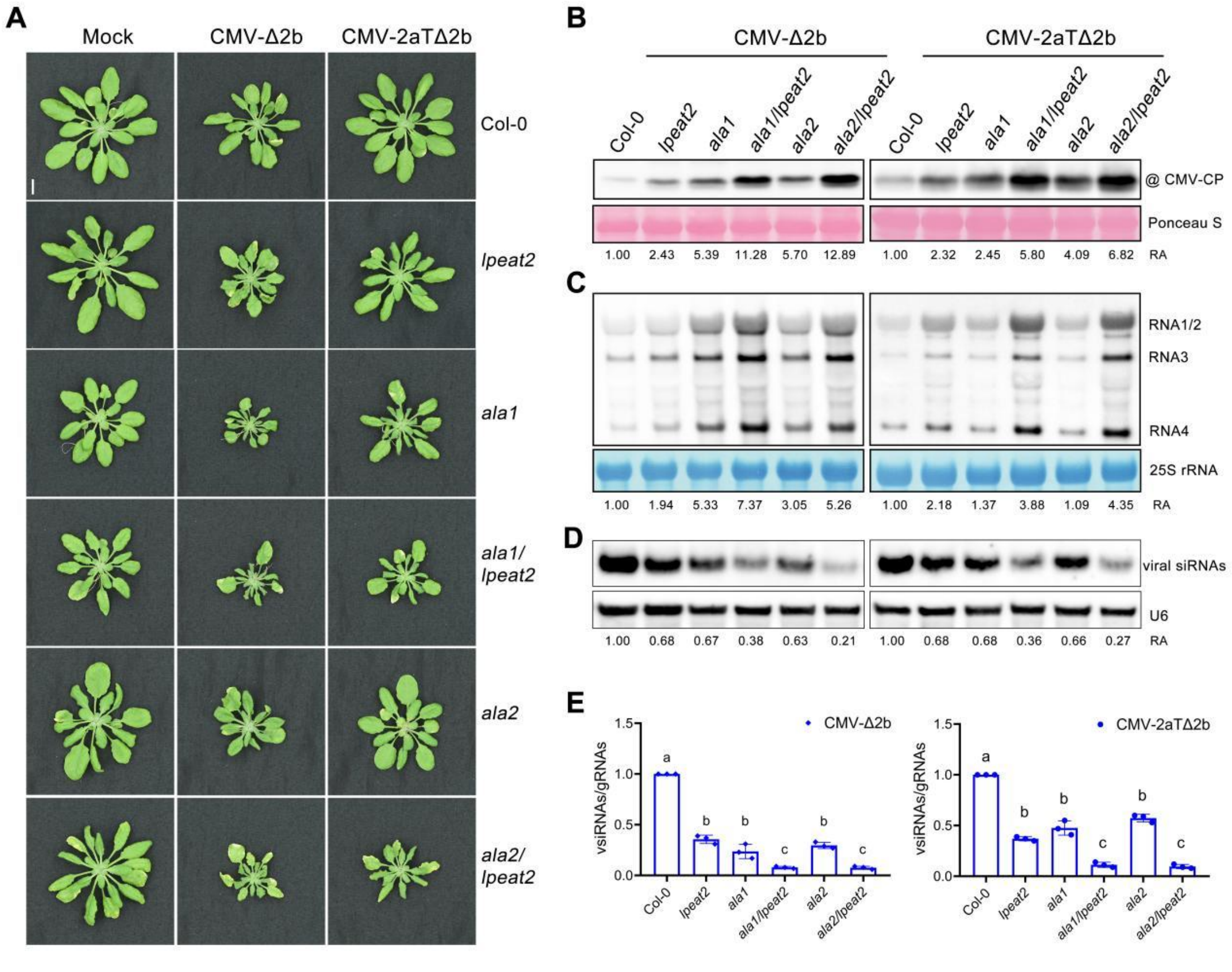

Figure S4. LPEAT2 functions independent of ALA1 and ALA2. (A) The phenotypes of plants photographed 14 days after inoculation with CMV-Δ2b, or CMV-2aTΔ2b or mock treatment with buffer, Bar= 1 cm. (B) Western blot analyses to detect the accumulation of virus coat protein in different plants, Rubisco in same membrane stained with ponceau staining to show equal loading. Relative accumulation (RA) of coat protein was calculated from Western blot signals using Image J. (C) and (D) Northern blot analyses of viral genomic RNAs or vsiRNAs accumulation in systemic leaves. 25S rRNA stained or U6

RNA probed as loading control, Relative accumulation (RA) of viral genomic RNAs or vsiRNAs was quantified using Image J by normalizing to 25S rRNA or U6, respectively. (E) Ratios of vsiRNAs/genomic RNAs were calculated from Northern hybridization signals in (C) and (D), with the ratio in Col-0 normalized to 1. The experiments were repeated three times independently with similar results. Data presented are means ± SEM from three replicates, letters indicate groups with significant differences (one-way ANOVA, Duncan, p<0.05) and dots represent the individual values.

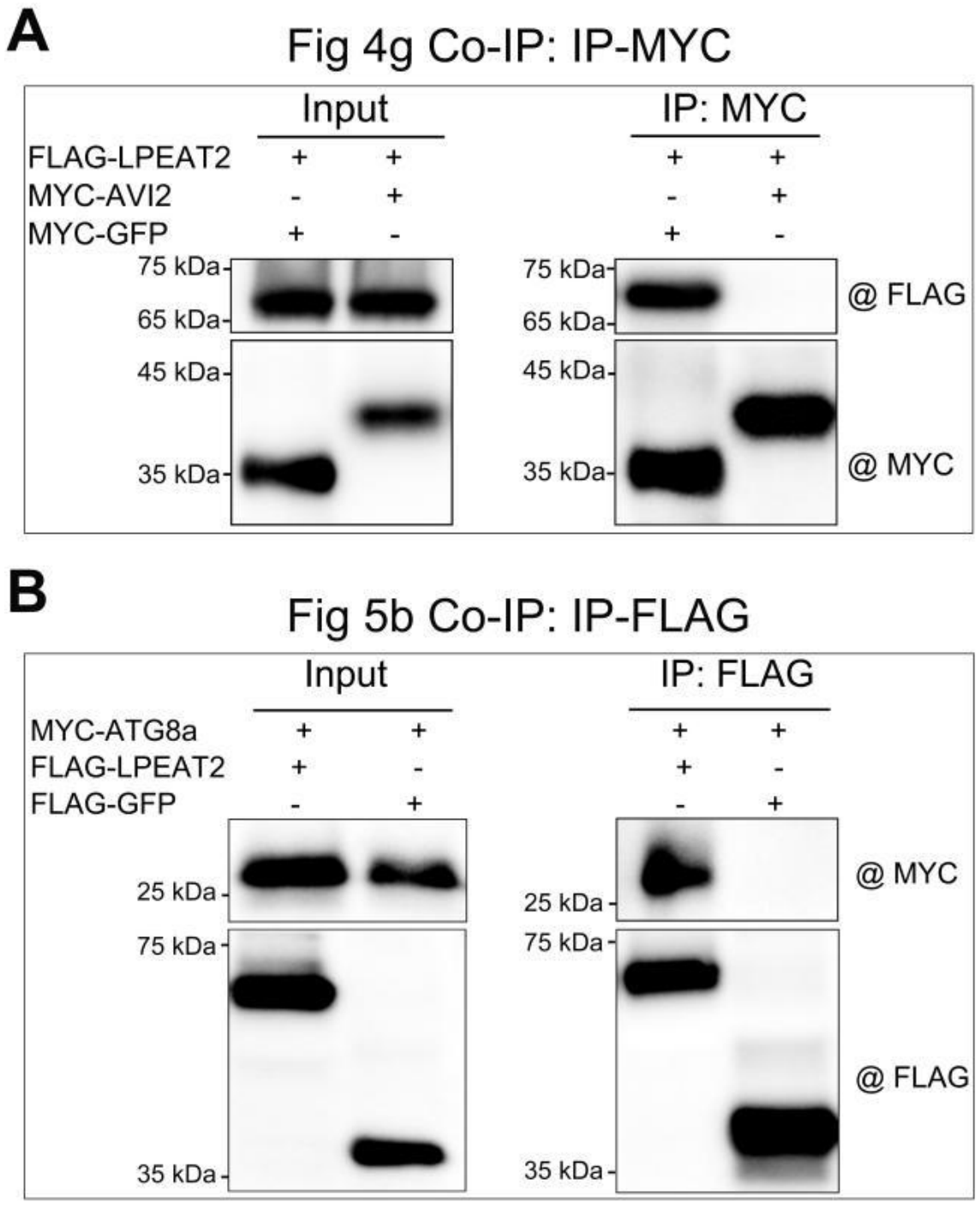

Fig. S5 Additional negative controls and experimental combinations for co-immunoprecipitation (Co-IP) assays corresponding to Fig. 4G and Fig. 5B. (A) Co-IP assay to assess the interaction between LPEAT2 and AVI2 proteins. (B) Co-IP assay to assess the interaction between LPEAT2 and ATG8a proteins.

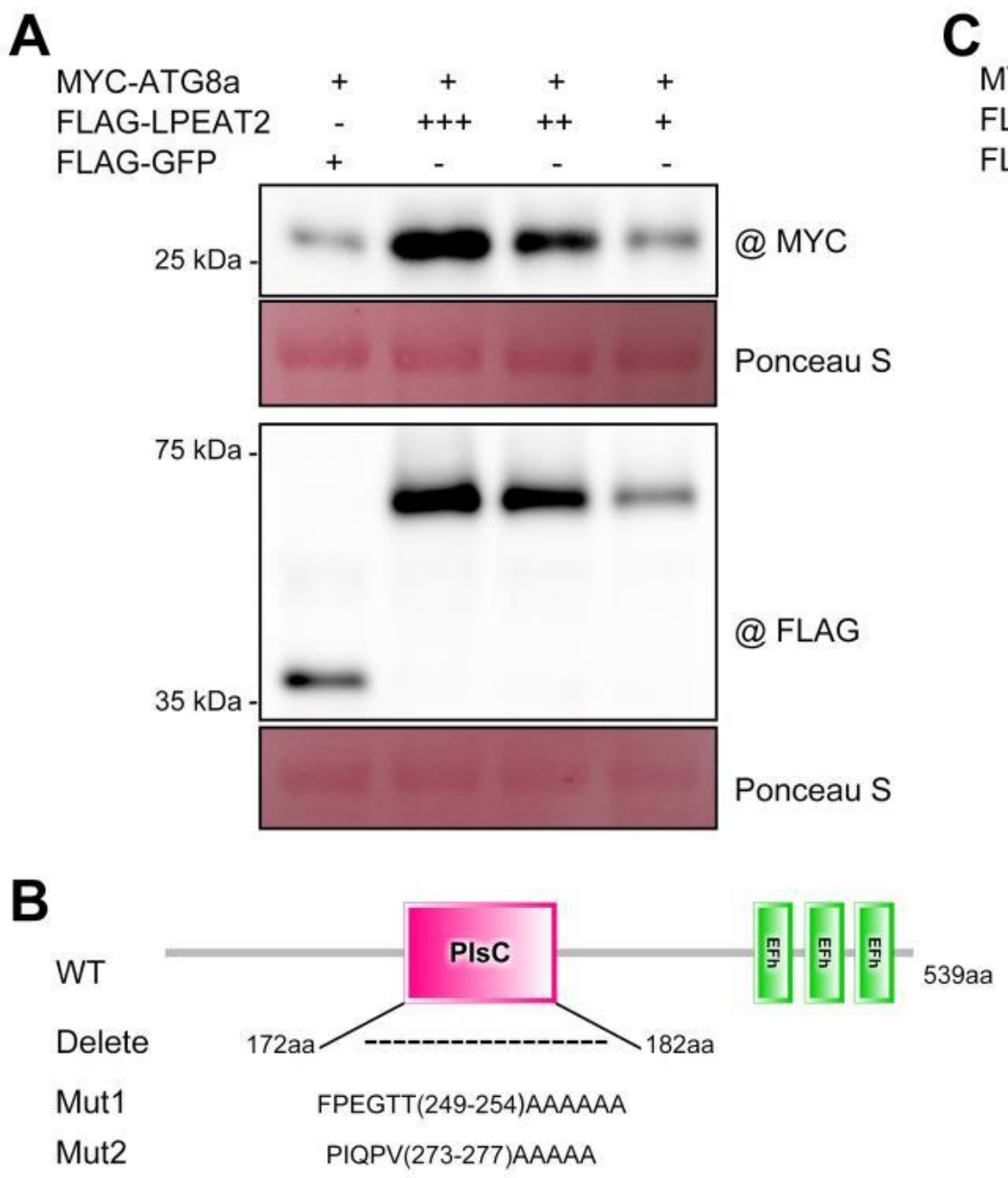

Fig. S6 The acyltransferase activity of LPEAT2 is essential for stabilizing the ATG8a protein. (A) AtLPEAT2 and AtATG8a were co-expressed in *N. benthamiana*, and protein accumulation levels were examined via Western blot assay using specific antibodies; Ponceau staining of Rubisco was employed as the loading control. (B) Schematic diagram of LPEAT2 acyltransferase activity-deficient mutants: the deletion mutation was generated by removing the 111-amino-acid (aa) PlsC domain, while the point mutations were constructed by substituting conserved amino acid residues with alanine. (C) Wild-type LPEAT2, LPEAT2 with deletion mutation or point mutation, and a GFP control were respectively co-expressed with ATG8a in *N. benthamiana*. The protein accumulation levels were detected by Western blot assay, with ponceau staining of Rubisco serving as the loading control.

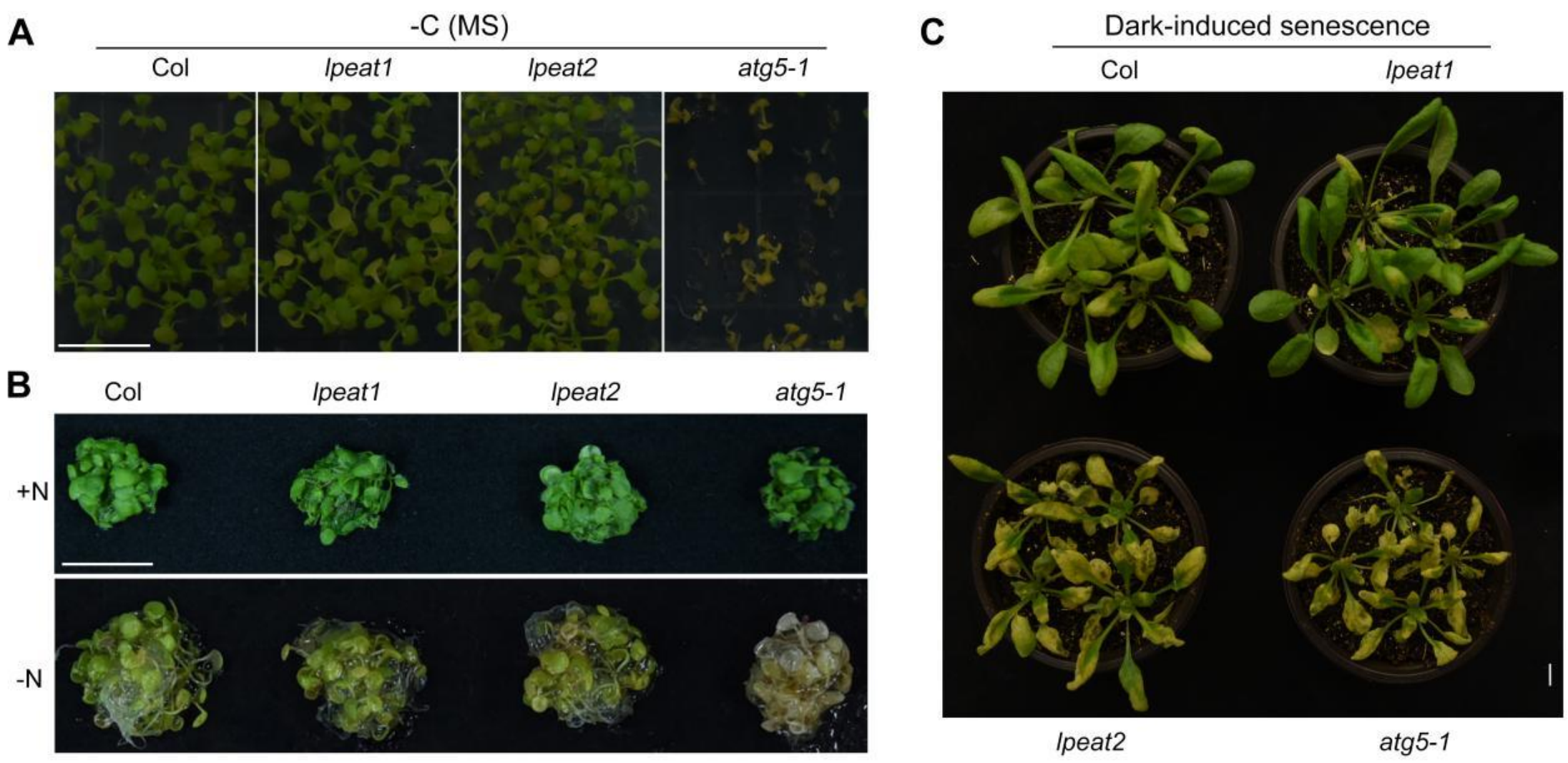

Figure S7. *lpeat2* mutants exhibit typical defects in autophagy under specific environmental stresses. (A) Plants were grown under long-day condition for 10 days in carbon-deficient MS (MS-C) medium plates, were then subjected to a 7-day dark period

treatment. Bar=1 cm. (B) Plants were grown under continuous light condition for 7 days on MS solid medium, were then transferred to Nitrogen-sufficient (+N) or Nitrogen-deficient (–N) MS liquid medium for an additional 7 days. Bar=1 cm. (C) enhanced darkness-induced senescence in *lpeat2*, *atg5-1*, compared to Col-0 and *lpeat1*. Plants were grown under long-day condition for approximately one month, were then subjected to a 7-day dark treatment, followed by a 5-7 day recovery period under long-day condition. Bar=1 cm.

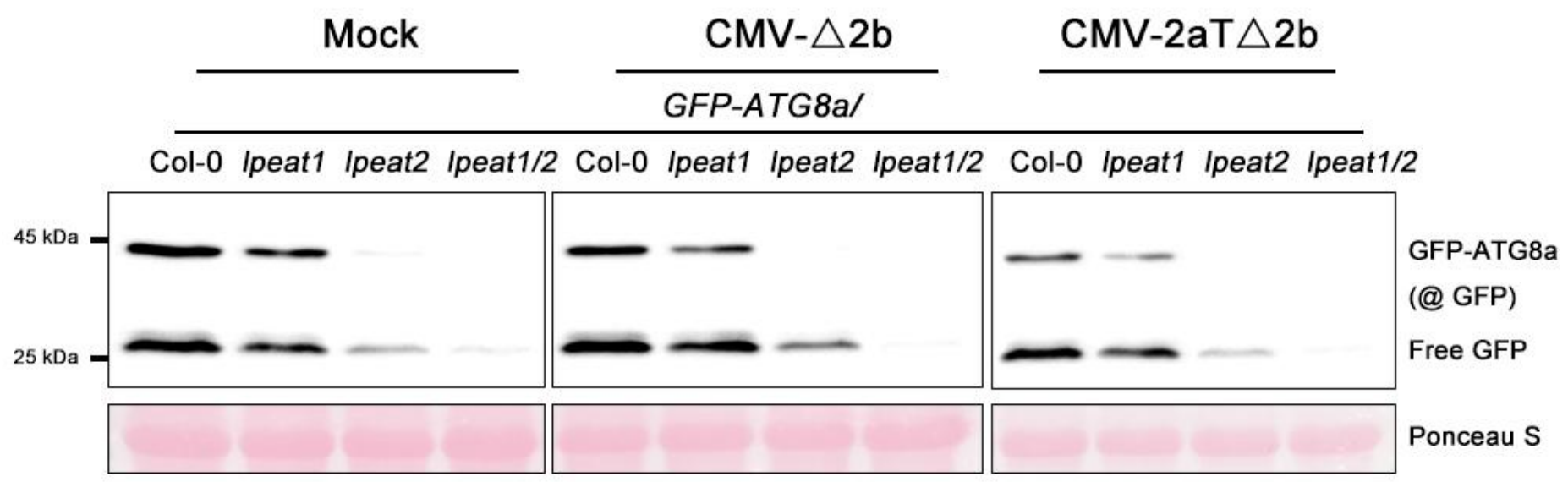

Figure S8. The accumulation of GFP-ATG8a protein in different crossed plants. Western blot analyses to detect the accumulation of GFP-ATG8a and free GFP proteins in plants after infection with CMV-Δ2b, CMV-2aTΔ2b, or mock treatment. Total proteins were probed with GFP antibodies, Rubisco in same membrane stained with ponceau staining to show equal loading.

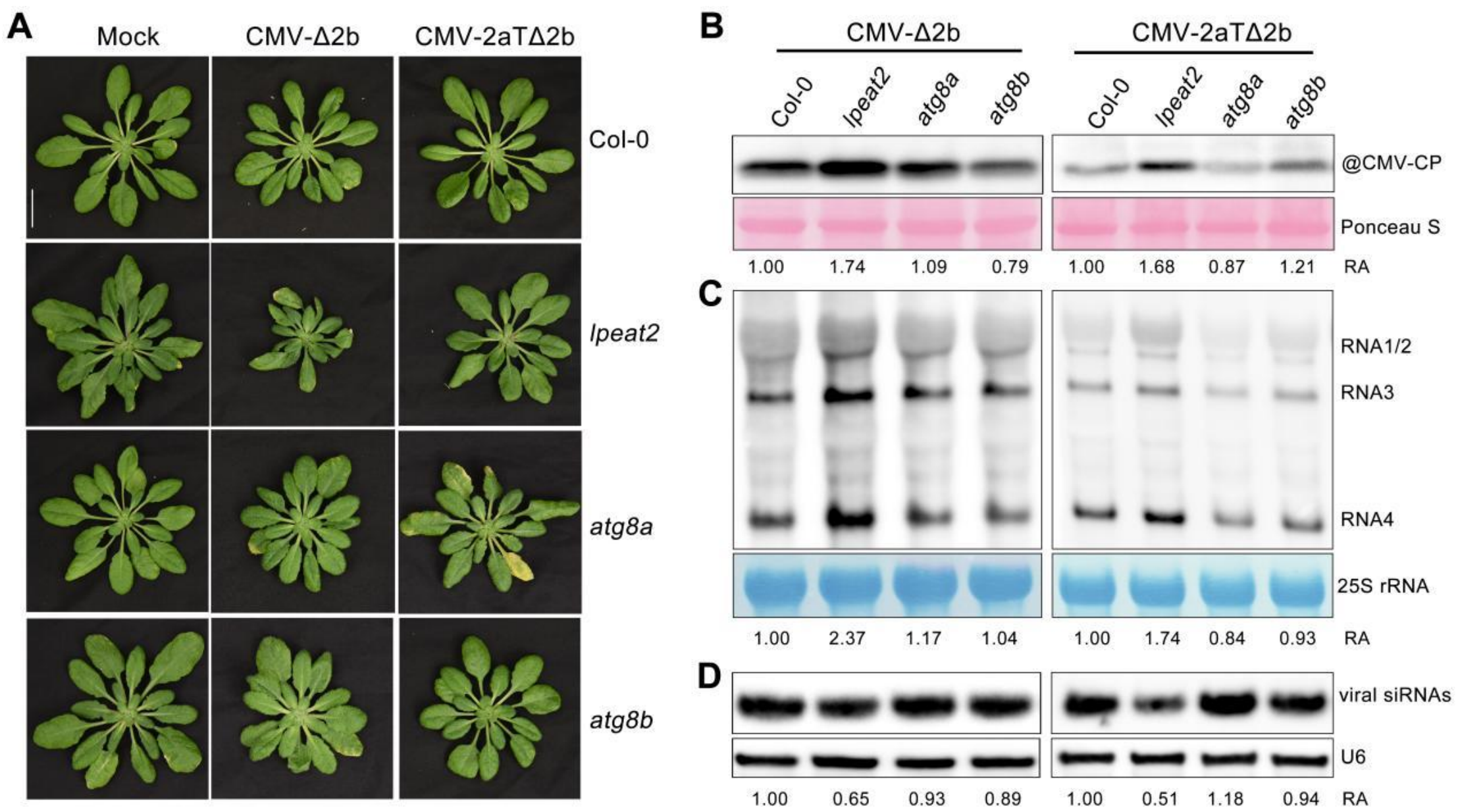

Figure S9. *atg8a* or *atg8b* single mutants did not show antiviral defects. (A) plants were photographed 2 weeks after infection with CMV-Δ2b, CMV-2aTΔ2b, or mock treatment, Bar= 1 cm. (B) Western blot analyses to detect the accumulation of virus coat protein in plants, Rubisco in same membrane stained with ponceau staining to show equal loading. Relative accumulation (RA) of coat protein was calculated from Western blot signals using Image J. (C) and (D) Northern blot analyses of viral genomic RNAs or

vsiRNAs accumulation in systemic leaves. 25S rRNA stained or U6 RNA probed as loading control, Relative accumulation (RA) of viral genomic RNAs or vsiRNAs was quantified using Image J by normalizing to 25S rRNA or U6, respectively.

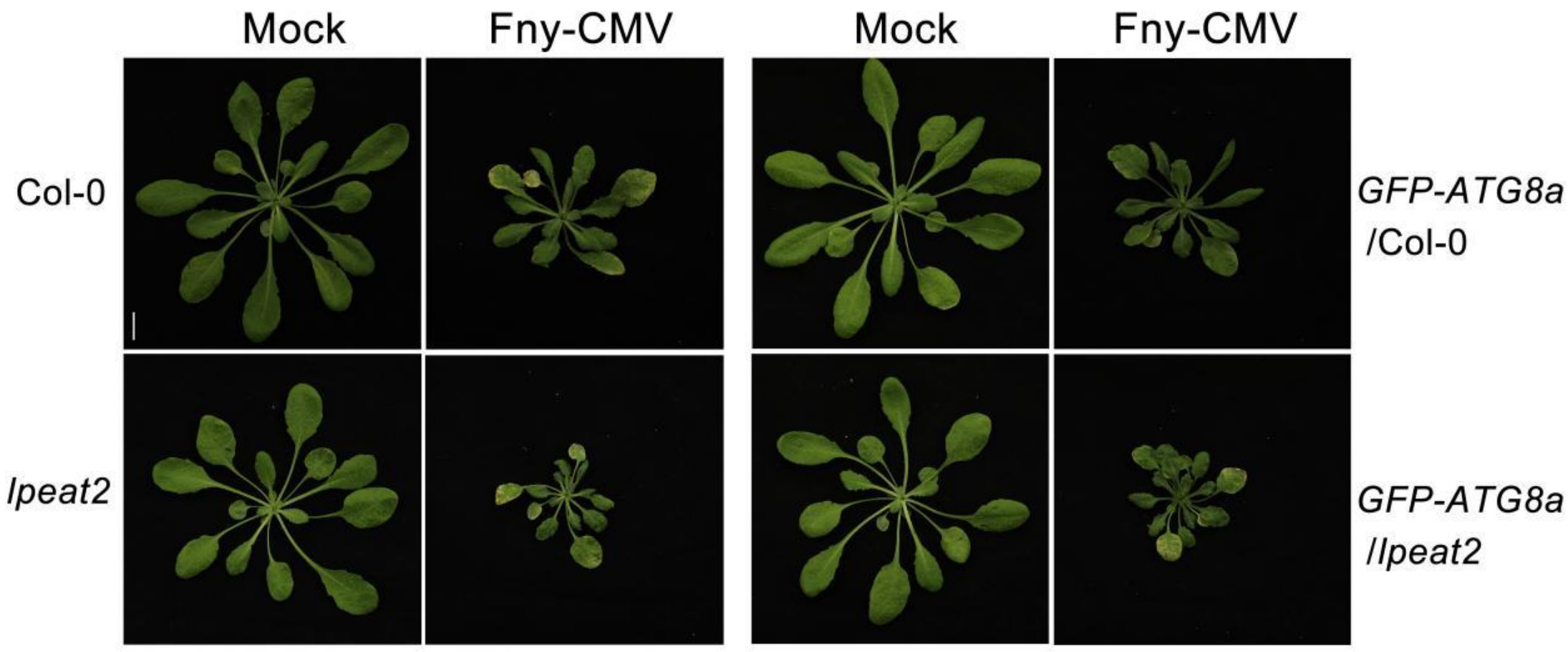

Fig S10. Phenotype of plants infected with Fny-CMV. The plants photographed 14 days after inoculation with Fny-CMV or mock treatment, Bar= 1 cm.

Fig. 2E Repeat#2

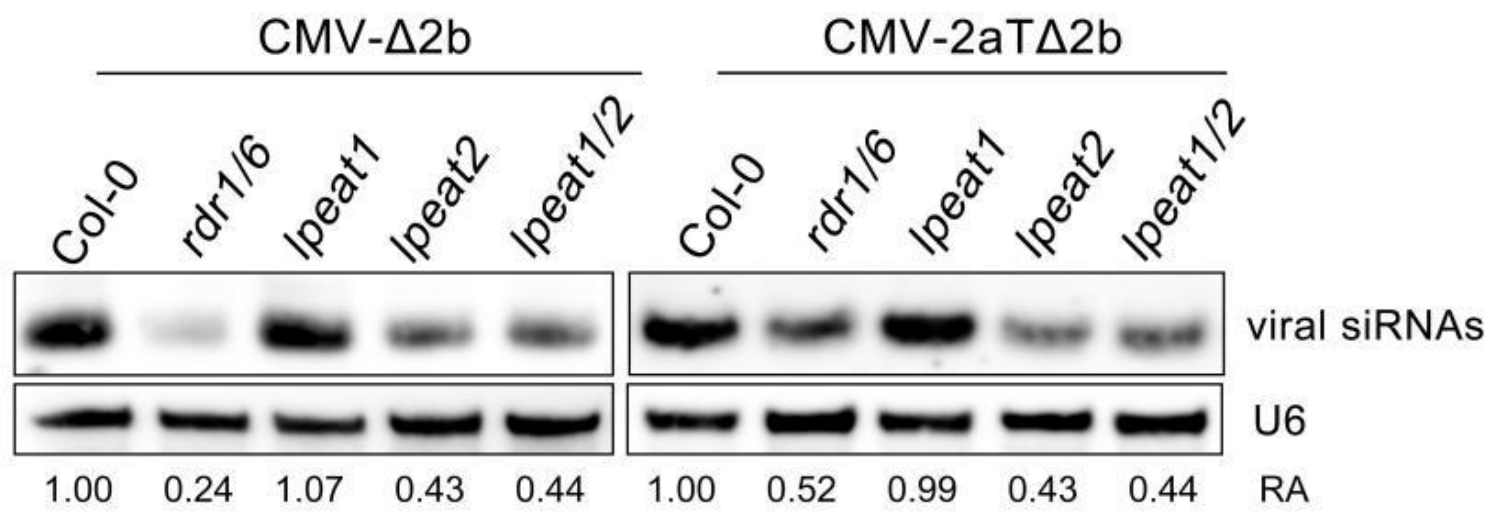

Fig. 2E Repeat#3

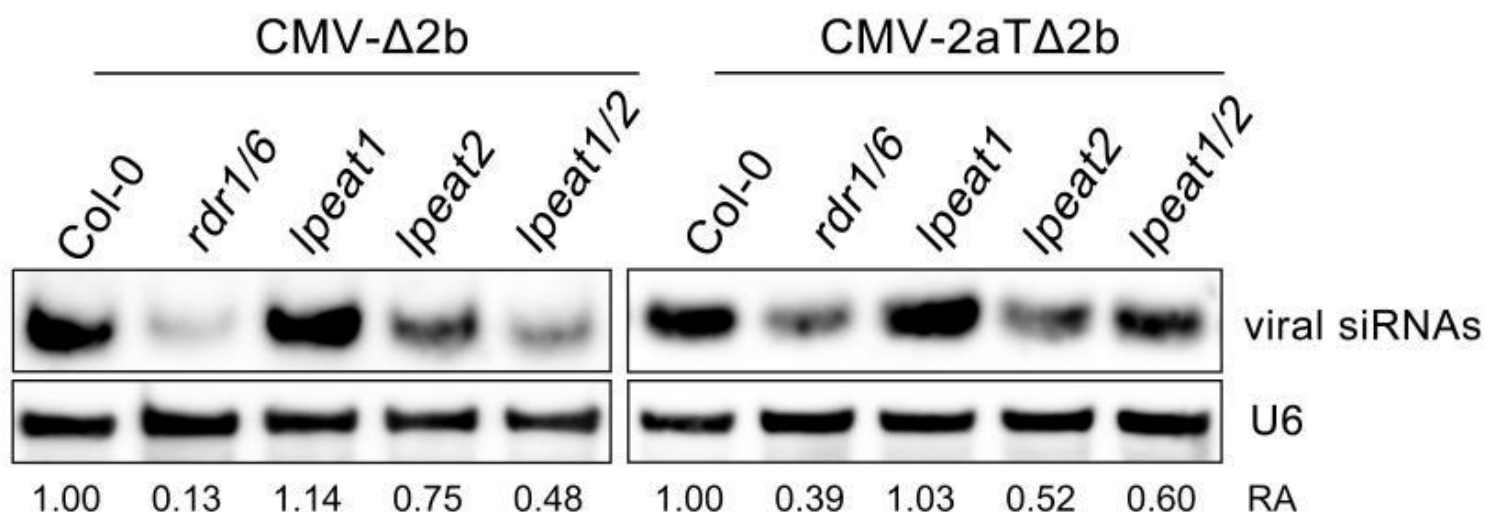

Fig. S11 Two additional biological replicates of viral siRNA detection via Northern blot assay, corresponding to Fig. 2E.

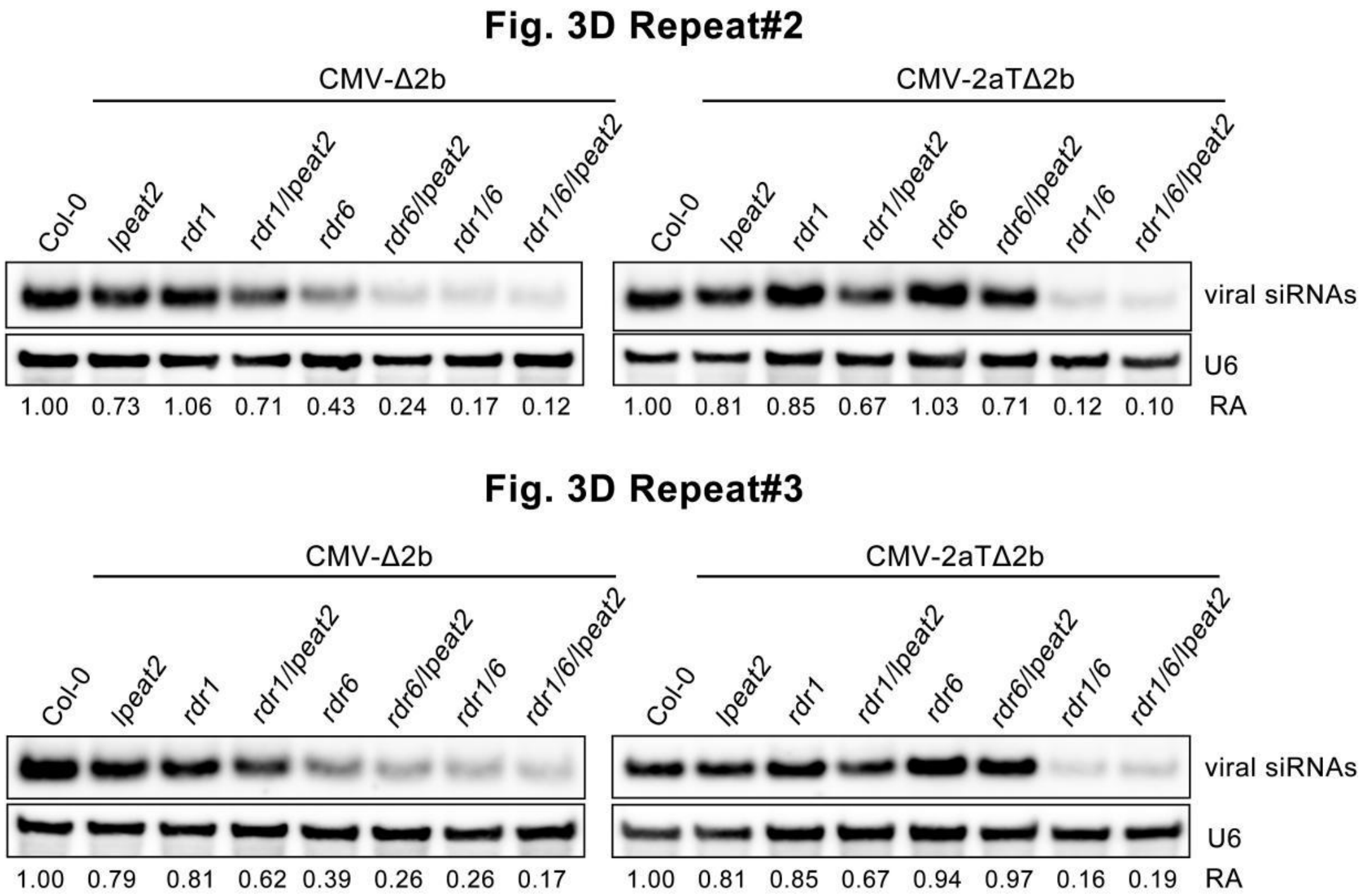

Fig. S12 Two additional biological replicates of viral siRNA detection via Northern blot assay, corresponding to Fig. 3D.

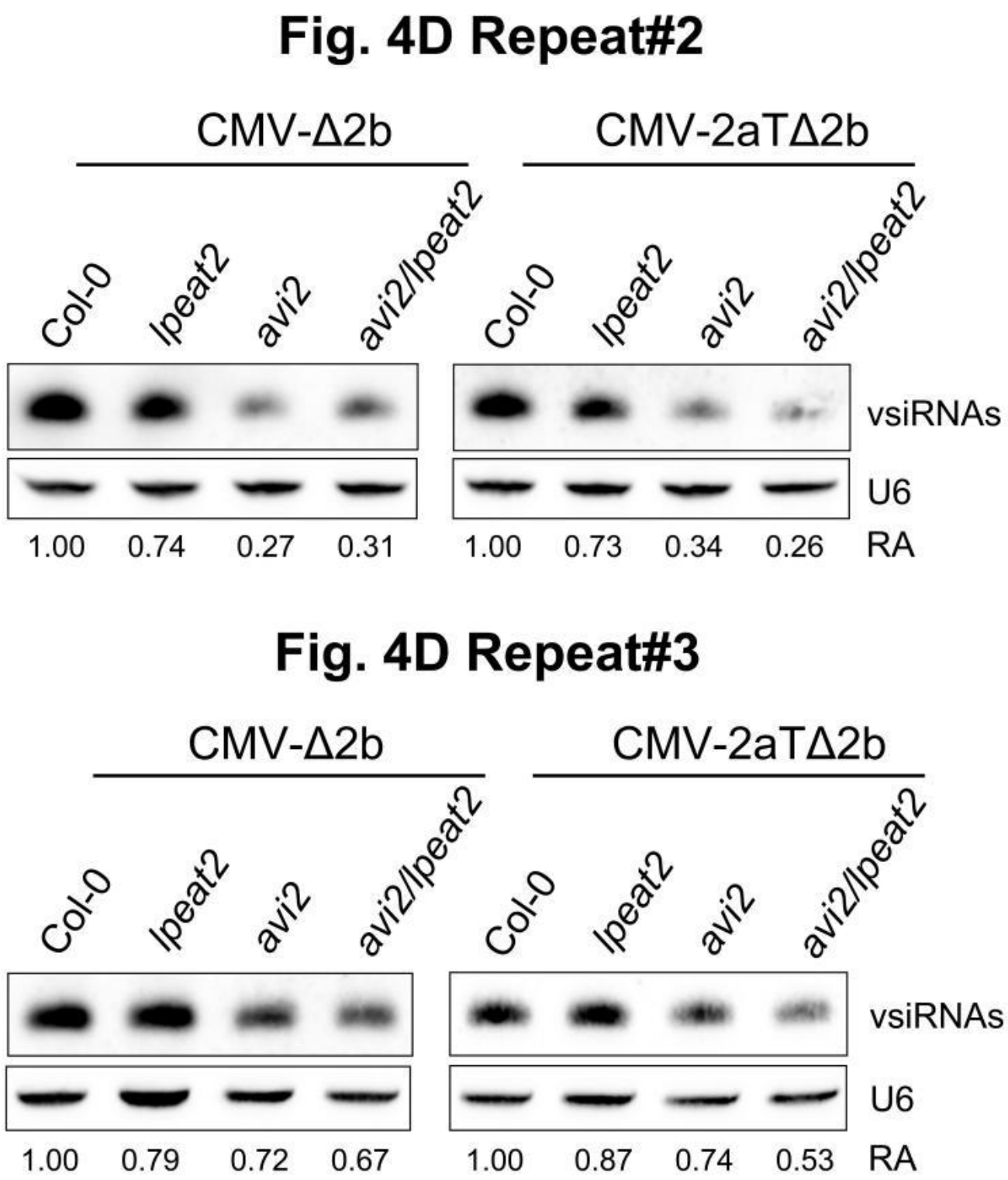

Fig. S13 Two additional biological replicates of viral siRNA detection via Northern blot assay, corresponding to Fig. 4D.

Fig 7D Repeat #2

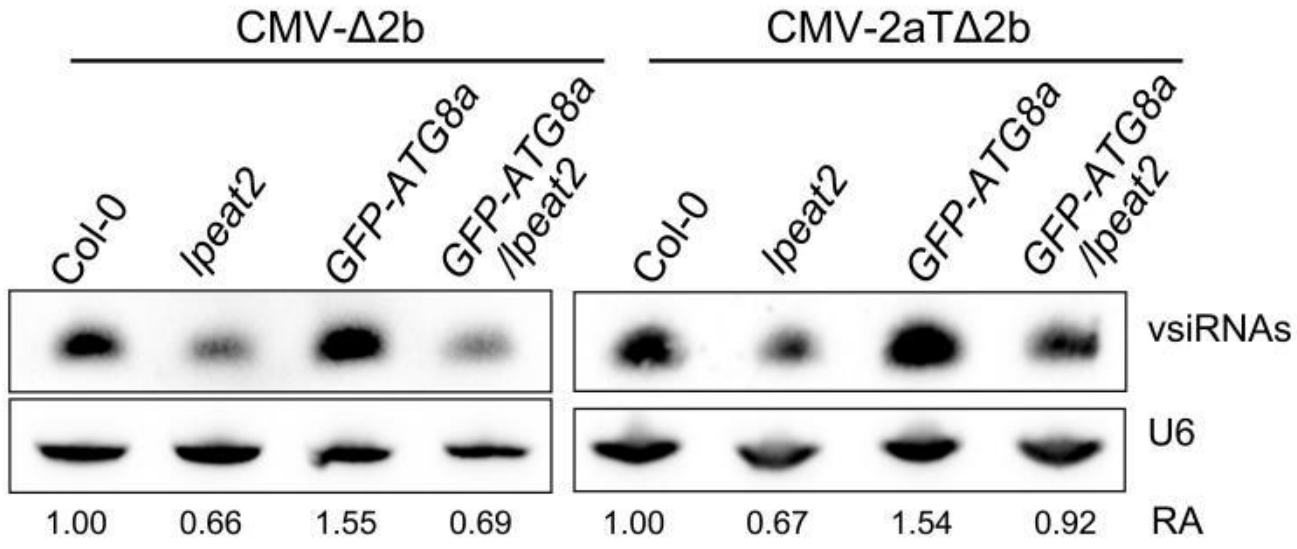

Fig 7D Repeat #3

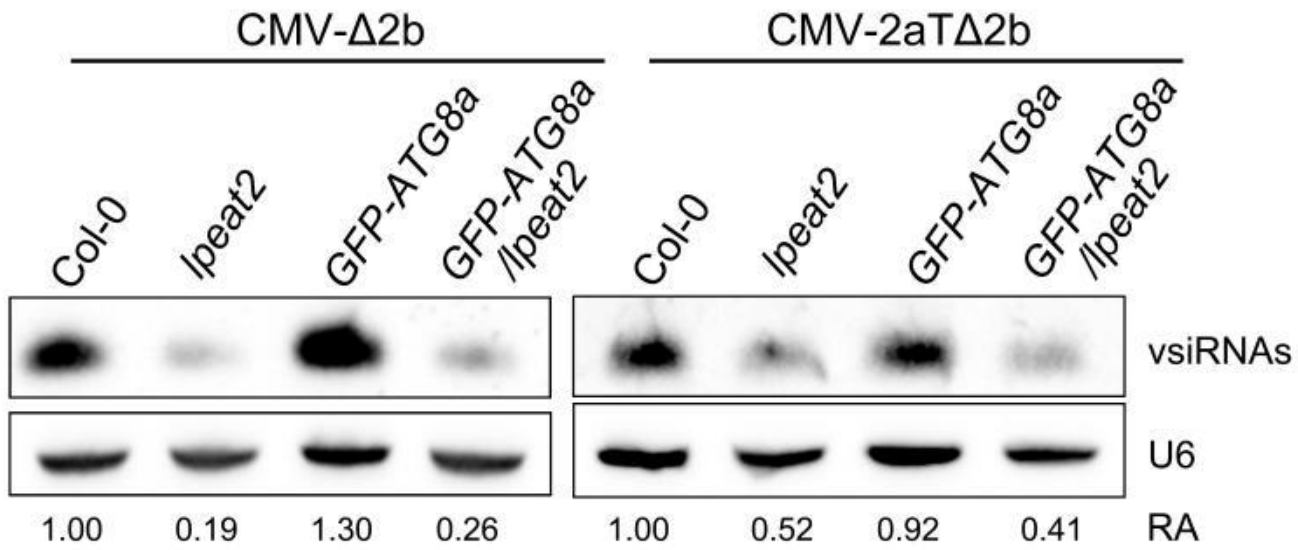

Fig. S14 Two additional biological replicates of viral siRNA detection via Northern blot assay, corresponding to Fig. 7D.

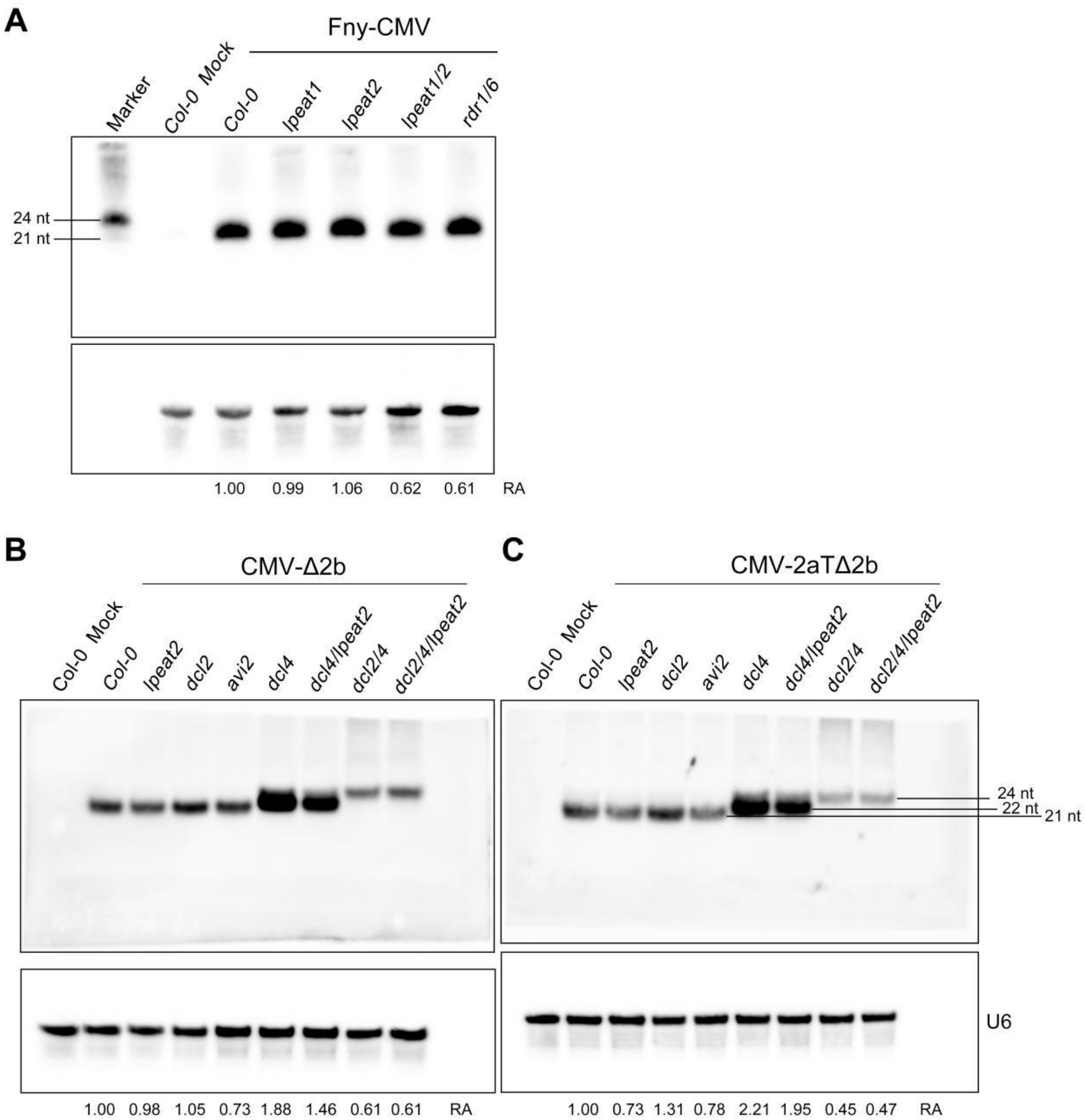

Fig. S15 Northern blot analyses of vsiRNAs produced in different Arabidopsis plants. (A) After wildtype Fny-CMV infection, 21 nt vsiRNAs were detected in Col-0, *lpeat1*, *lpeat2*, *lpeat1/2* and *rdr1/6* plants, the size of 21 nt vsiRNAs was indicated with small RNA marker. U6 RNA serving as the loading control. (B-C) after the infection of CMV-Δ2b (B) or CMV-2aT2b (C), 21 nt vsiRNAs were produced in Col-0, *lpeat2, dcl2, avi2*, compared to 22 nt viral siRNAs exclusively produced in *dcl4* and *dcl4 lpeat2* double mutants and 24 nt viral siRNAs only produced in *dcl2 dcl4* and *dcl2 dcl4 lpeat2* mutants. U6 RNA serving as the loading control.

Fig. 2E Original figures

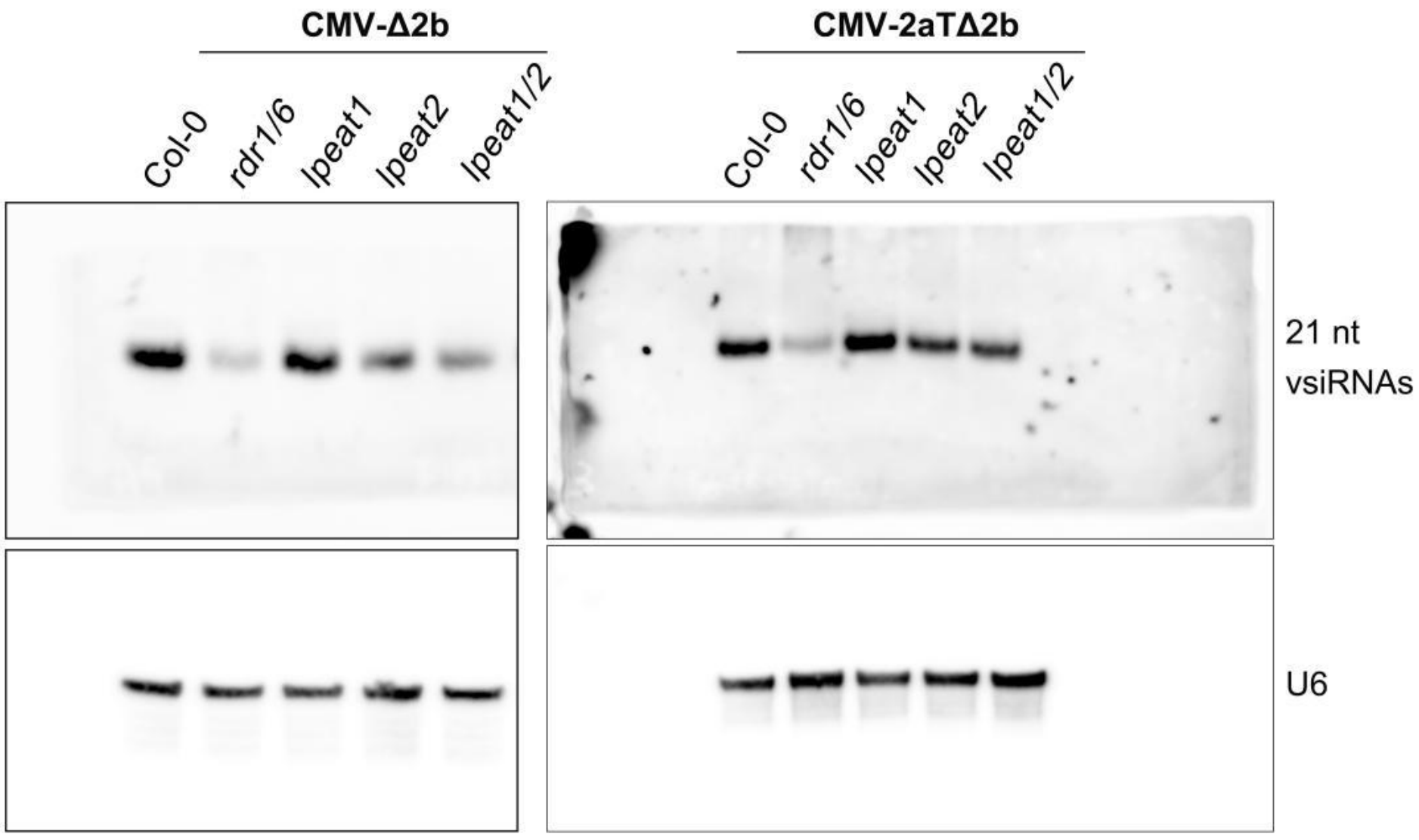

Fig. 3D Original figures

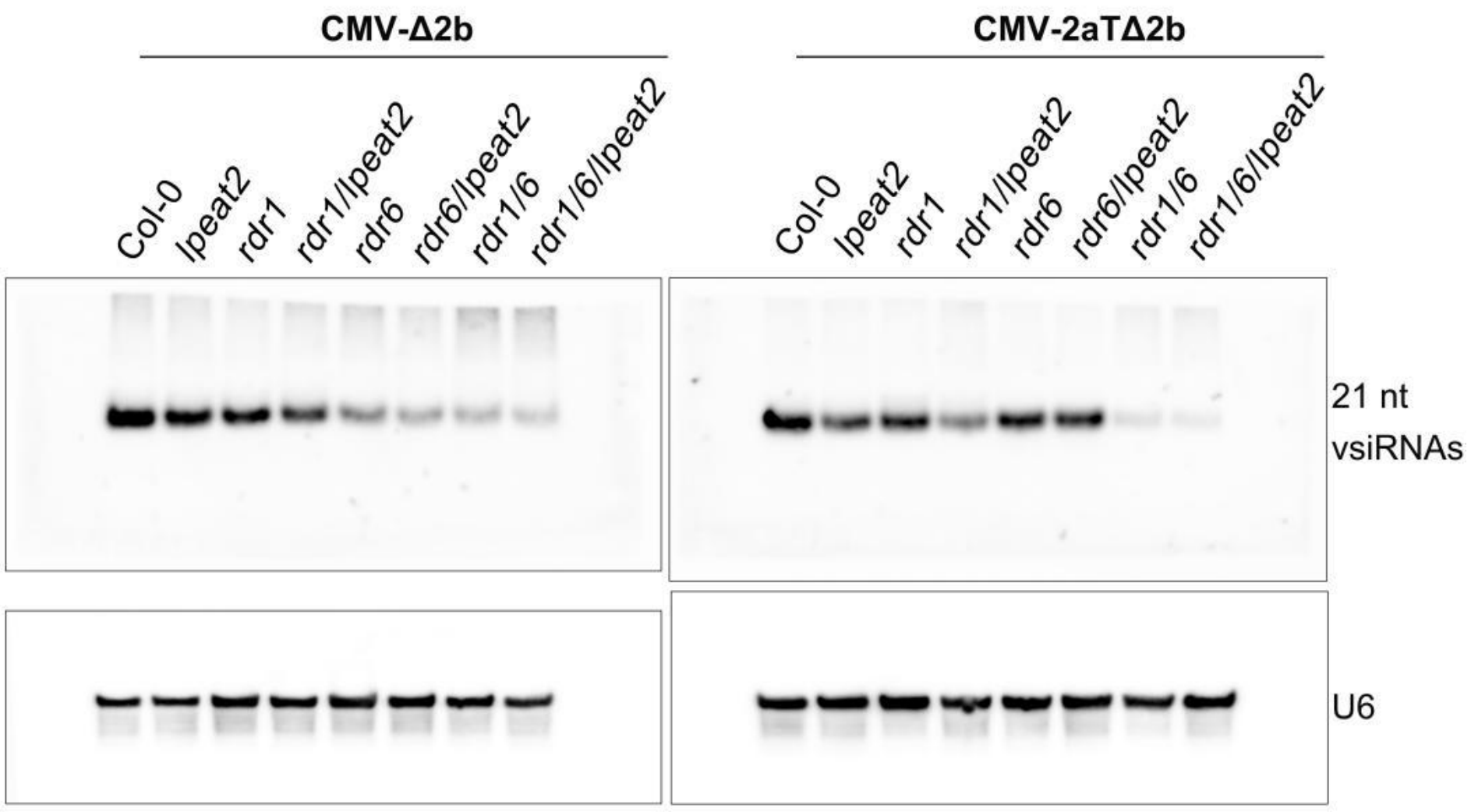

Fig. S16 The original images of Northern blot assay corresponding to Fig. 2E (up) or Fig. 3D (down), respectively.

Table S1. Primers used in the study

Name	5' to 3' Sequences
**Primers used for genotyping mutants by PCR**	
lpeat1 SALK_016798-LP	TTCAAAGCATGAGATAACCGG
lpeat1 SALK_016798-RP	AGTTTTGGGGCAATTTATTGG
lpeat2 SALK_073928-LP	TGGCTGATTCTTTGATCTTGG
lpeat2 SALK_073928-RP	ACATGGTTGAAATGGCAAGAG
lpeat2 SALK_107699-LP	ATGTATCCAAAAATCGCGTTG
lpeat2 SALK_107699-RP	AGCTTCCTGCGATAGATTTCC
atg8a SALK_045344-LP	TTTTCTGGGTTTGTTTCTCCC
atg8a SALK_045344-RP	TCATTAGCCCCAAAATCAATG
atg8b SALK_036033-LP	ACGAAAACGTTGAAAAACGAG
atg8b SALK_036033-RP	TTCTTCTCCATGATGATTCGG
LBb1.3	ATTTTGCCGATTTCGGAAC
**Primers used for quantitative RT-PCR**	
LPEAT1-qF	CTGTTTTCTGCTCCTTATCGTG
LPEAT1-qR	CGAAAAGCAAAACCCTAGAGAG
LPEAT2-qF	CGACGACCAAGATCAAAGAATC
LPEAT2-qR	GTGCAAAGATGGCTATGAGAAG
PP2A-qF	ATCGCTACTCAGTTTAACCACA
PP2A-qR	TAGCAATAGTTTGGGGCACTAA
SULFUR-qF	CTTCTGCAATCTGGGCTTCTC
SULFUR-qR	CAGTAGAGTTGATTTCAGTGGCT
**Primers used for *lpeat2* mutant complementation analysis**	
LPEAT2 native promoter-F	aaaagcaggctccgcGCATAATTCGAAACGTTTCG
LPEAT2 native promoter-R	catggtgaagggggcGTGGTGGAATGTTATTTGTTTC
pGWB10-npLPEAT2-F	caccATGGCGGATCCTGATCTGTCTTCTC
pGWB10-npLPEAT2-R	TTATGTTGGGGCCAAGTCAGGTGCA
**Primers used for gene overexpression**	
p1300-myc-LPEAT2-F	tctgaagaggacttgaatggtaccATGGCGGATCCTGATCTGTCTTCTC
p1300-myc-LPEAT2-R	tacgaacgaaagctctgcaggtcgacTTATGTTGGGGCCAAGTCAGGTGCA
**Primers used for Bimolecular Fluorescence Complementation (BiFC)**	
pDONR221-LPEAT2-F	ggggacaagtttgtacaaaaaagcaggcttcATGGCGGATCCTGATCTGTCTTCTC
pDONR221-LPEAT2-R	ggggaccactttgtacaagaaagctgggtcTGTTGGGGCCAAGTCAGGTGCAAAGA
pDONR221-ATG8a-F	ggggacaagtttgtacaaaaaagcaggcttcATGATCTTTGCTTGCTTGAAATTCGC
pDONR221-ATG8a-R	ggggaccactttgtacaagaaagctgggtcAGCAACGGTAAGAGATCCAAAAGTG
pDONR221-AVI2-F	ggggacaagtttgtacaaaaaagcaggcttcATGGAGACGGACAATGGGAAG
pDONR221-AVI2-R	ggggaccactttgtacaagaaagctgggtcGTATTCAGGACTGTACAGGC
pDONR221-GUS-F	ggggacaagtttgtacaaaaaagcaggcttcATGGTCCGTCCTGTAGAAACCCCAA
pDONR221-GUS-R	ggggaccactttgtacaagaaagctgggtcTTGTTTGCCTCCCTGCTGCGGTT
**Primers used for co-immunoprecipitation (Co-IP)**	
p1300-myc-AVI2-F	tctgaagaggacttgaatggtaccATGGAGACGGACAATGGGAAG

p1300-myc-AVI2-R	tacgaacgaaagctctgcaggtcgacCTAGTATTCAGGACTGTACAGGC
p1300-myc-ATG8a-F	tctgaagaggacttgaatggtaccATGATCTTTGCTTGCTTGAAATTCGC
p1300-myc-ATG8a-R	tacgaacgaaagctctgcaggtcgacTCAAGCAACGGTAAGAGATCCAAAAGTG
p1300-myc-GFP-F	tctgaagaggacttgaatggtacc
p1300-myc-GFP-R	tacgaacgaaagctctgcaggtcgac
p3301-flag-LPEAT2-F	gacgatgacaagacgcgtcccATGGCGGATCCTGATCTGTCTTCTC
p3301-flag-LPEAT2-R	attatggagaaagcttggatccTTATGTTGGGGCCAAGTCAGGTGCA
**Primers used for yeast two-hybrid (Y2H)**	
pGAD-ATG8a-F	gccatggaggccagtgaattcATGATCTTTGCTTGCTTGAAATTCGC
pGAD-ATG8a-R	agctcgagctcgatggatcccTCAAGCAACGGTAAGAGATCCAAAAGTG
pGAD-LPEAT2-F	gccatggaggccagtgaattcATGGCGGATCCTGATCTGTCTTCTC
pGAD-LPEAT2-R	agctcgagctcgatggatcccTTATGTTGGGGCCAAGTCAGGTGCA
pGAD-AVI2-F	gccatggaggccagtgaattcATGGAGACGGACAATGGGAAG
pGAD-AVI2-R	agctcgagctcgatggatcccCTAGTATTCAGGACTGTACAGGC
pGBK-ATG8a-F	atggccatggaggccgaattcATGATCTTTGCTTGCTTGAAATTCGC
pGBK-ATG8a-R	cgctgcaggtcgacggatcccTCAAGCAACGGTAAGAGATCCAAAAGTG
pGBK-LPEAT2-F	atggccatggaggccgaattcATGGCGGATCCTGATCTGTCTTCTC
pGBK-LPEAT2-R	cgctgcaggtcgacggatcccTTATGTTGGGGCCAAGTCAGGTGCA
pGBK-AVI2-F	atggccatggaggccgaattcATGGAGACGGACAATGGGAAG
pGBK-AVI2-R	cgctgcaggtcgacggatcccCTAGTATTCAGGACTGTACAGGC

F, forward; R, reverse.
