# Supplementary material for: Antiviral RNAi defective 4 (AVI4) encodes a lysophospholipid acyltransferase coopting with AVI2 and ATG8a to promote antiviral immunity in Arabidopsis
Source: Plant Commun. 2026 Apr 14;7(9):101868. doi: 10.1016/j.xplc.2026.101868 (PMC13589543; doi:10.1016/j.xplc.2026.101868)
Supplement: Supplementary file 2 [file mmc2.pdf]

Plant Communications
Research article

CellPress Partner Journal

Antiviral RNAi defective 4 (AVI4) encodes a lysophospholipid acyltransferase coopting with AVI2 and ATG8a to promote antiviral immunity in *Arabidopsis*

Wenkai Yan[2,3,4], Yaogui Zhou[1,2,4], Bei Jia[2,4,5], Jiangshuai Niu[1,2], Shihuai Jin[1,2], Zhuoning Zhao[1,2] and Zhongxin Guo[1,2,*]

[1]College of Life Sciences, Shandong Agricultural University, Tai'an, Shandong, China

[2]Vector-borne Virus Research Center, State Key Laboratory for Ecological Pest Control of Fujian and Taiwan Crops, College of Plant Protection, Fujian Agriculture and Forestry University, Fuzhou, China

[3]State Key Laboratory for Quality and Safety of Agro-Products, Key Laboratory of Biotechnology in Plant Protection of MARA, Key Laboratory of Green Plant Protection of Zhejiang Province, Institute of Plant Virology, Ningbo University, Ningbo, China

[4]These authors contributed equally to this article.

[5]Present address: Industrial Crop Research Institute, Heilongjiang Academy of Agricultural Sciences, Harbin, China

***Correspondence: Zhongxin Guo (zhongxin5919@163.com)**

https://doi.org/10.1016/j.xplc.2026.101868

ABSTRACT

RNA interference (RNAi)-mediated antiviral immunity is an evolutionarily conserved mechanism preventing virus infection in eukaryotes. In recent studies, we developed a sensitized genetic screen to identify *antiviral RNAi-defective* (*avi*) mutants for uncovering components of the antiviral pathway in *Arabidopsis*. We revealed that AVI2 is a novel regulator that enhances the biogenesis of viral small interfering RNAs (vsiRNAs) to promote small-RNA-based antiviral immunity. However, the molecular mechanisms that modulate the efficiency of RNAi-mediated antiviral immunity remain largely unclear. Here, we identified the *avi4* mutant using the same genetic screen. Mapping by sequencing identified a T-DNA insertion in *LPEAT2* as the causal mutation for *avi4*, and this result was confirmed through analysis of an allelic *lpeat2* mutant and complementation analysis. LPEAT2, a lysophospholipid acyltransferase in *Arabidopsis*, functions in the biosynthesis of phospholipids (especially phosphatidylethanolamine [PE]) and modulation of the autophagy pathway. We revealed that LPEAT2, but not its homolog LPEAT1, interacts with AVI2 to enhance RDR1/6-dependent vsiRNA biogenesis. Interestingly, LPEAT2 also interacts with and stabilizes the core autophagy component ATG8a and promotes autophagy before or after viral infection. Notably, overexpression of ATG8a enhances vsiRNA production and antiviral resistance, which is dependent on LPEAT2 function and can be repressed by VSR 2b. Therefore, our research demonstrates that AVI4 (LPEAT2) is a novel regulator of RNAi-mediated antiviral immunity, it cooperates with AVI2 and ATG8a to enhance vsiRNA biogenesis for robust antiviral defense in *Arabidopsis*.

Key words: AVI4, antiviral RNAi, AVI2, ATG8a, autophagy

Yan W., Zhou Y., Jia B., Niu J., Jin S., Zhao Z., and Guo Z. (2026). Antiviral RNAi defective 4 (AVI4) encodes a lysophospholipid acyltransferase coopting with AVI2 and ATG8a to promote antiviral immunity in *Arabidopsis*. Plant Comm. **7**, 101868.

INTRODUCTION

RNA interference (RNAi) is a gene-silencing mechanism that is evolutionarily conserved across eukaryotes (Hannon, 2002). This small-RNA-based mechanism regulates all aspects of plant growth and development (Baulcombe, 2004; Chen, 2009) and also plays a fundamental role in plant antiviral immunity (Guo et al., 2019; Lopez-Gomollon and Baulcombe, 2022). Research on the model plant *Arabidopsis* has shown that double-stranded viral RNAs produced after viral infection are recognized by various Dicer-like (DCL) proteins, which initiate antiviral RNAi by generating primary viral small interfering RNAs (vsiRNAs)

© 2026 The Author(s). Published by Elsevier Inc. on behalf of CAS Center for Excellence in Molecular Plant Sciences, Chinese Academy of Sciences, and Chinese Society for Plant Biology.
This is an open access article under the CC BY license (http://creativecommons.org/licenses/by/4.0/).

(Diaz-Pendon et al., 2007). DCL4, DCL2, and DCL3, together with the double-stranded RNA-binding proteins DRBs, produce vsiRNAs of 21, 22, or 24 nucleotides, respectively, to target a wide range of plant RNA and DNA viruses, whereas DCL1 primarily generates endogenous microRNAs that may indirectly modulate antiviral RNAi (Xie et al., 2004; Diaz-Pendon et al., 2007). A key step in vsiRNA biogenesis is the amplification of secondary vsiRNAs by RNA-dependent RNA polymerases (RDRs) such as RDR1 and the RDR6–SGS3 complex (Mourrain et al., 2000; Wang et al., 2010; Yoshikawa et al., 2021). The primary and secondary vsiRNAs are loaded into effector Argonaute proteins (AGOs) to induce efficient antiviral defense through transcriptional or post-transcriptional gene silencing (Wang et al., 2011; Brosseau et al., 2016).

During the evolutionary arms race between hosts and viruses, viruses have developed viral suppressors of RNA silencing (VSRs) that target various steps of the antiviral RNAi pathway, enabling them to evade host immunity (Jin et al., 2021). Cucumber mosaic virus (CMV), a model plant RNA virus with significant agricultural impacts, is a natural pathogen of *Arabidopsis* and many crops (Scholthof et al., 2011; Jones, 2021). Its 2b protein, a well-characterized VSR, inhibits both vsiRNA amplification and AGO activity, serving as a key virulence factor for CMV (Watt et al., 2020). Two engineered 2b-defective CMV mutants have recently been developed: CMV-Δ2b, in which the start codon of *2b is mutated from AUG to ACG,* and CMV-2aTΔ2b, in which bases 1–295 of the *2b* sequence have been deleted. These mutants were used to develop a sensitized genetic screen for identifying components of the antiviral RNAi machinery in *Arabidopsis*, leading to the discovery of several novel regulators of antiviral immunity (Guo et al., 2017, 2018; Liu et al., 2022). Notably, AVI2, an unknown protein, was recently shown to promote antiviral RNAi by enhancing RDR1/RDR6-mediated vsiRNA amplification (Guo et al., 2018), although its detailed functional mechanism remains to be characterized.

Phosphatidylethanolamine (PE), a primary component of lipid bilayers in cellular membranes, plays essential roles in maintaining membrane structure, responding to stress, and supporting organelle function in plants (Otsuru et al., 2013; Völz et al., 2021). LPEAT proteins are acyltransferases that acylate lysophospholipid substrates to produce phospholipids, especially PE, in *Arabidopsis* (Stålberg et al., 2009; Jasieniecka-Gazarkiewic et al., 2016). Although both LPEAT1 and LPEAT2 exhibit lysophosphatidyl acyltransferase activity, LPEAT2 is more critical for normal plant growth and development through maintenance of PE levels (Stålberg et al., 2009; Jasieniecka-Gazarkiewicz et al., 2017). Interestingly, LPEAT2, but not LPEAT1, has been found to participate in the autophagy pathway of *Arabidopsis* by modulating accumulation of the ATG8 complex, a core component of the autophagic machinery that is usually conjugated with PE to facilitate autophagosome formation in plants (Jasieniecka-Gazarkiewicz et al., 2021). The autophagy pathway plays a pivotal role in modulating plant–virus interactions (Yang and Liu, 2022; Kang et al., 2024; Sharma et al., 2024), and different isoforms of ATG8 (ATG8a–ATG8i) have been reported to positively or negatively regulate plant antiviral immunity (Li et al., 2020a, 2020b; Tong et al., 2021; Cao et al., 2023). However, whether LPEATs participate in antiviral resistance and their potential functional mechanisms in plants remain to be determined.

In this study, we characterized *avi4, a new antiviral RNAi-defective mutant isolated through our previously developed genetic screen, and demonstrated that the causal gene AVI4* (*LPEAT2*) encodes a key regulator of antiviral RNAi. We found that LPEAT2, but not its close homolog LPEAT1, promoted antiviral RNAi by interacting with AVI2 to enhance RDR1/RDR6-dependent vsiRNA production. Further analyses revealed that LPEAT2 also interacted with and stabilized ATG8a to facilitate autophagy. Notably, overexpression of ATG8a increased vsiRNA production and antiviral resistance, LPEAT2 was indispensable for the vsiRNA-based antiviral function of ATG8a, and their functions could be repressed by VSR 2b upon wild-type CMV infection. Thus, AVI4 emerges as a novel regulator that cooperates with AVI2 and ATG8a to efficiently produce vsiRNAs for small-RNA-based antiviral immunity, providing robust defense against viral infections in *Arabidopsis*.

RESULTS

Identification of *antiviral RNAi defective 4* (*avi4*)

A sensitized genetic screen, previously developed for isolating *antiviral RNAi-defective* (*avi*) mutants (Guo et al., 2017, 2018), identified *Salk_107644* as the *avi4* mutant. This mutant exhibited severe disease symptoms upon infection with CMV-Δ2b but showed no visible defects compared with Columbia-0 (Col-0) in the absence of viral infection (Figure 1A). Northern blot analysis showed that the enhanced susceptibility of *avi4* was associated with significantly increased virus accumulation compared with Col-0 plants (Figure 1B). Surprisingly, no T-DNA insertion was identified in the *AT2G37230* gene. To clone the causal mutation gene, we backcrossed *avi4* to Col-0, generating an F2 population for mapping-by-sequencing analysis as described previously (Guo et al., 2017) (Supplemental Figure 1A). Approximately one-quarter of the F2 plants were susceptible to CMV-Δ2b, suggesting that *avi4* is caused by a single recessive mutation. Two separate pools of symptomatic and asymptomatic plants (50 individuals each) were subjected to whole-genome sequencing, and further comparative analyses identified a T-DNA insertion in the seventh exon of *AT2G45670*, which encodes *LPEAT2* in *Arabidopsis* (Supplemental Figure 1B). Interestingly, the T-DNA insertion matched that of *Salk_107699*, indicating that *avi4* (*Salk_107699*) may have been accidently designated *Salk_107644*.

AVI4 encodes LPEAT2 in *Arabidopsis*

To confirm the T-DNA insertion in *LPEAT2*, we complemented *avi4* with the wild-type *LPEAT2* gene driven by its native promoter. Transgenic plants expressing LPEAT2 showed no disease symptoms upon infection with CMV-Δ2b or CMV-2aTΔ2b (Figure 1D). Northern blot analysis confirmed that viral RNA accumulation in the complemented lines was rescued to Col-0 levels, providing evidence that *avi4* resulted from *LPEAT2* mutation (Figure 1E). Another allelic mutant, *Salk_073928*, which contained a T-DNA insertion in the 10th exon of *LPEAT2* (Figure 1C), also exhibited severe disease symptoms after infection with CMV-Δ2b, although symptoms were less pronounced upon infection with CMV-2aTΔ2b (Figure 1D).

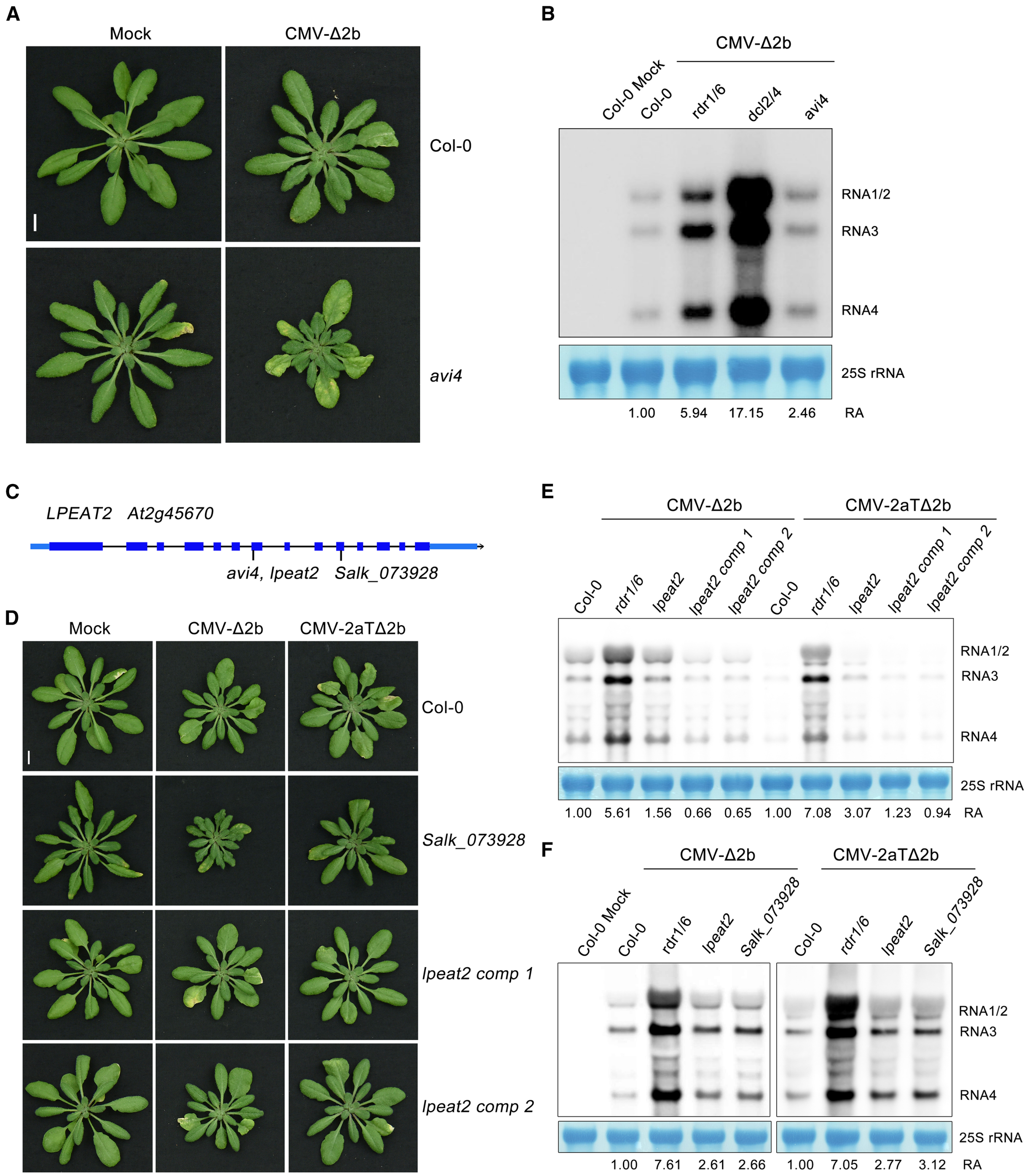

Figure 1. *AVI4* encodes *LPEAT2* in *Arabidopsis*.
(A) Plants photographed 14 days after inoculation with CMV-Δ2b or mock treatment. Scale bar, 1 cm.
(B) Northern blot analyses of viral genomic RNA accumulation in non-inoculated systemic leaves of plants; 25S rRNA on the same membrane was stained with Coomassie blue to show equal loading. Relative accumulation (RA) of viral genomic RNA was calculated by normalizing to 25S rRNA using ImageJ.
(C) Schematic diagram of the *LPEAT2* genomic structure and T-DNA insertion sites in *avi4*, *lpeat2*, and *Salk_073928*.
(D) Plants photographed 14 days after inoculation with CMV-Δ2b, CMV-2aTΔ2b, or buffer (mock). Scale bar, 1 cm.
(E and F) Northern blot analyses of viral genomic RNA accumulation in non-inoculated systemic leaves of the plants shown in **(D)**.

Northern blot analyses revealed that viral RNA accumulation in *Salk_073928* was comparable to that in *avi4* (Figure 1F). Together, these results confirmed that *avi4* is caused by mutation of *LPEAT2*.

LPEAT2 specifically promotes antiviral RNAi by enhancing vsiRNA production

LPEAT2 encodes a lyso-PE acyltransferase that transfers acyl groups from acyl-coenzyme A to phospholipid substrates in

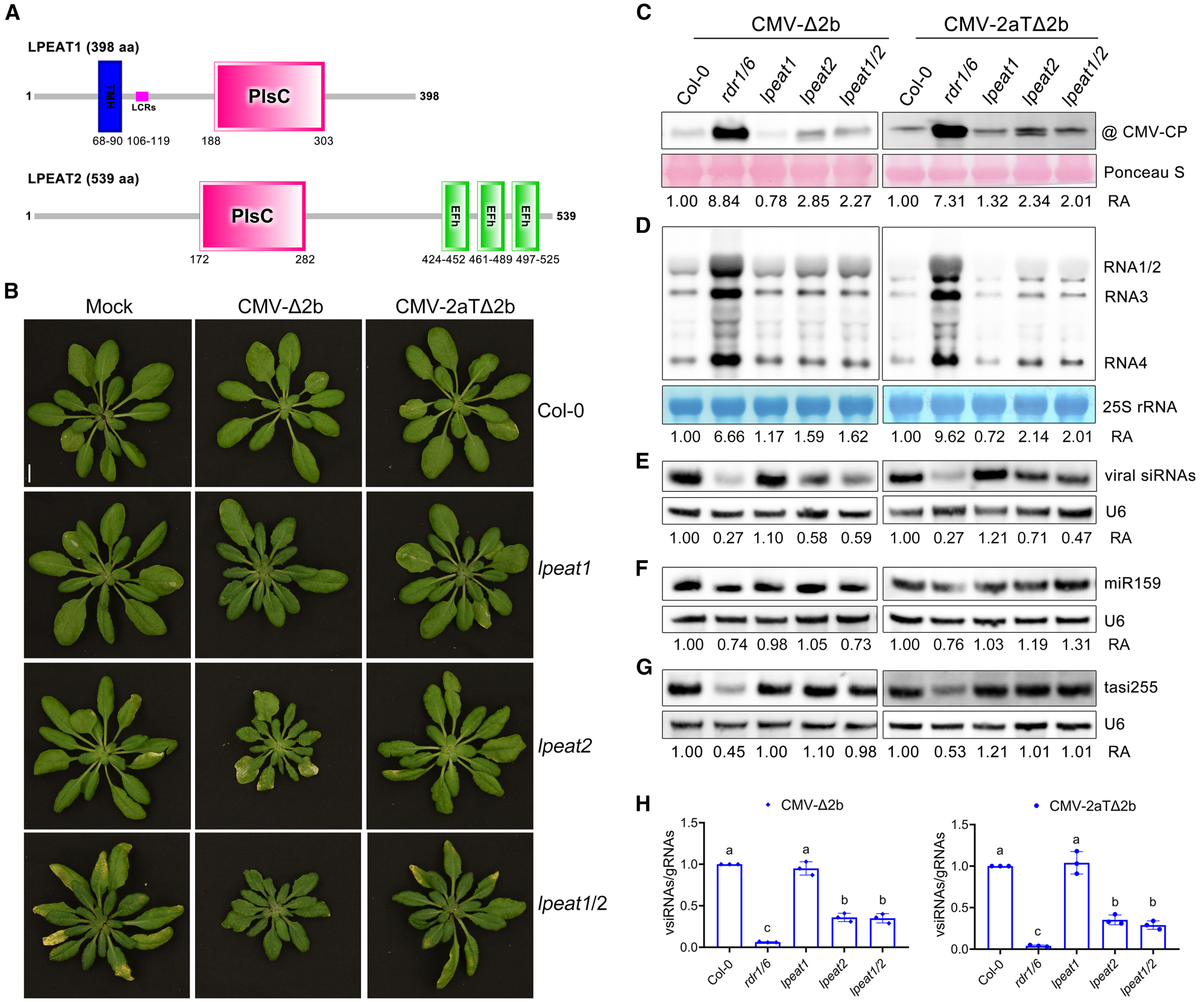

Figure 2. LPEAT2 promotes antiviral RNAi by enhancing vsiRNA production.

(A) Schematic diagram of LPEAT1 and LPEAT2 protein structures with functional domains.

(B) Plants photographed 14 days after inoculation with buffer (mock), CMV-Δ2b, or CMV-2aTΔ2b. Scale bar, 1 cm.

(C) Western blot analyses of viral coat protein accumulation in plants. Rubisco stained with Ponceau S was used as a loading control. Relative accumulation (RA) of coat protein was calculated using ImageJ.

(D and E) Northern blot analyses of viral genomic RNA or vsiRNA accumulation in systemic leaves. 25S rRNA staining or a U6 RNA probe was used as a loading control. Relative accumulation (RA) of viral genomic RNA or vsiRNA was quantified using ImageJ by normalizing to 25S rRNA or U6, respectively.

(F and G) Accumulation of miR159 **(F)** and tasiRNA255 **(G)** was detected by northern blotting in non-inoculated systemically infected leaves shown in **(E)**.

(H) Ratios of vsiRNA/genomic RNA were calculated from the northern hybridization signals in **(D)** and **(E)**, with the ratio in Col-0 normalized to 1.

The experiments in **(D)** and **(E)** were repeated independently three times with similar results. Data are presented as means ± SEM from independent experiments; letters indicate significant differences (one-way ANOVA, Duncan's test, $p < 0.05$), and dots represent individual values.

Arabidopsis (Stålberg et al., 2009; Jasieniecka-Gazarkiewicz et al., 2017). There is a single *LPEAT2* homolog in *Arabidopsis*, namely *LPEAT1* (*AT1g80950*). Both LPEAT1 and LPEAT2 proteins contain an acyltransferase (PlsC) domain; however, LPEAT2 possesses three additional EF-hand domains at its C terminus (Figure 2A). Interestingly, CMV infection induced the expression of *LPEAT2* but not *LPEAT1* (Supplemental Figure 2A and 2B). We obtained *Salk_016798*, a previously characterized *lpeat1* mutant (Jasieniecka-Gazarkiewicz et al., 2017). In contrast to *lpeat2*, *lpeat1* plants displayed no visible disease symptoms after CMV-Δ2b or CMV-2aTΔ2b infection, compared with Col-0 (Figure 2B). We next generated the *lpeat1 lpeat2* double mutant by crossing *avi4* with *Salk_016798*. After infection with CMV-Δ2b or CMV-2aTΔ2b, *lpeat1 lpeat2* developed disease symptoms similar to those of the *lpeat2* single mutant (Figure 2B). Consistent with the observed disease symptoms, the accumulation of viral coat protein (CP) and genomic RNA in *lpeat1* was unchanged relative to that in Col-0 after CMV-Δ2b or CMV-2aTΔ2b infection. However, the *lpeat1 lpeat2* double mutant exhibited significantly higher accumulation of viral CP and genomic RNA after infection with either virus, similar to that of the *lpeat2* single mutants (Figure 2C and 2D).

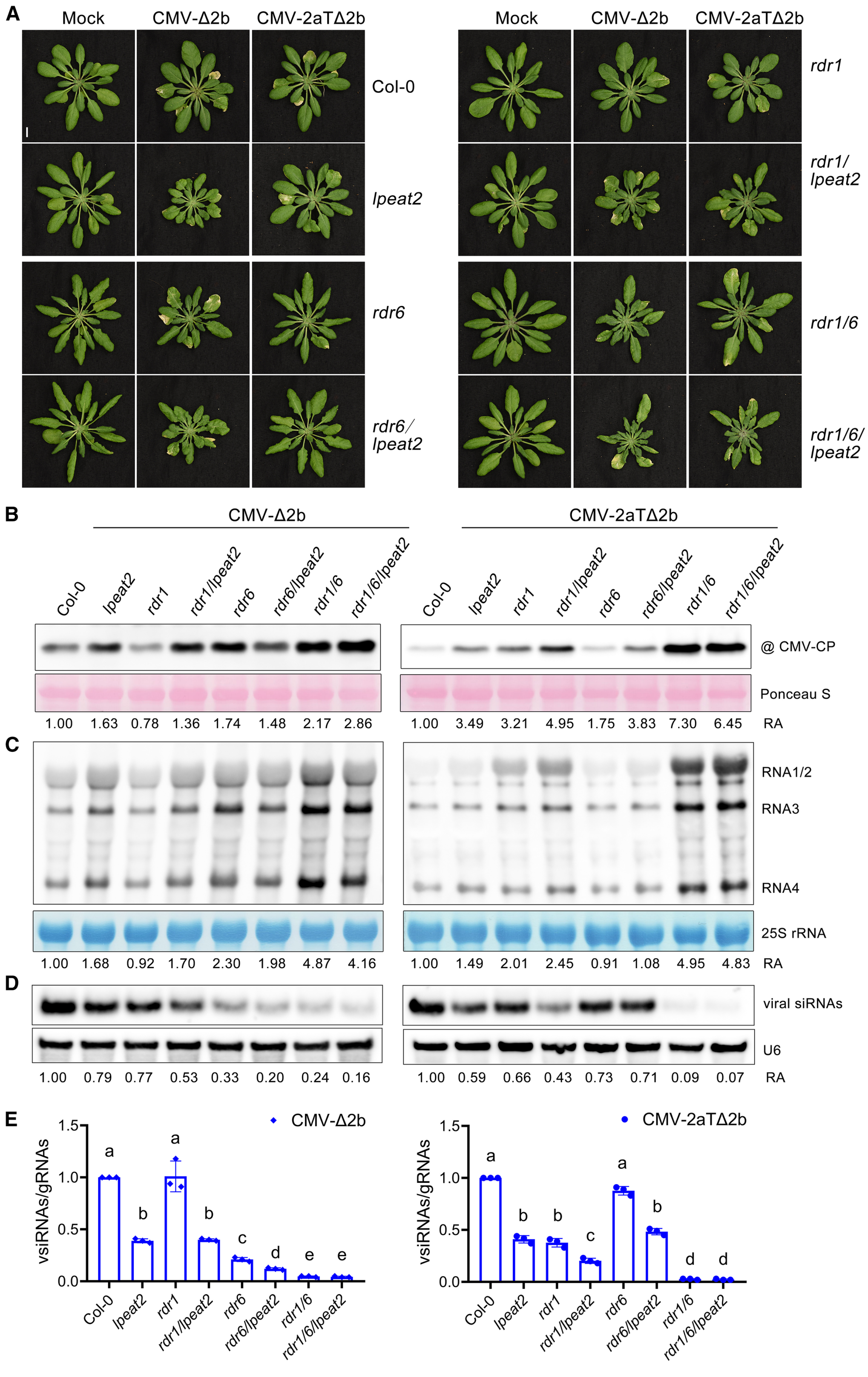

Figure 3. LPEAT2 modulates RDR1/6-mediated antiviral RNAi.

(A) Plants photographed 14 days after mock inoculation or infection with CMV-Δ2b or CMV-2aTΔ2b. Scale bar, 1 cm.

(B) Western blot analyses to detect the accumulation of virus coat protein in different plants shown in Figure 2C.

(legend continued on next page)

These findings indicate that LPEAT2, but not LPEAT1, plays a significant role in defense against viral infection.

vsiRNA is the core factor that initiates and determines antiviral RNAi (Guo et al., 2019). To determine whether antiviral RNAi was compromised, we examined vsiRNA production in Col-0, the *lpeat1* and *lpeat2* single mutants, and the *lpeat1 lpeat2* double mutant. Northern blot analyses demonstrated that vsiRNA production was significantly reduced in *lpeat2* (Figure 2E and Supplemental Figure 11) and in the *lpeat1/2* double mutant relative to Col-0 (Figure 2E and Supplemental Figure 11). However, mutation of *LPEAT1* alone did not affect vsiRNA production (Figure 2E and Supplemental Figure 11). The ratio of vsiRNA/viral RNA was also dramatically reduced in *lpeat2* and *lpeat1 lpeat2* but not in *lpeat1* (Figure 2H). These results demonstrate that LPEAT2 promotes antiviral RNAi by enhancing vsiRNA production, whereas LPEAT1 does not play an important role in antiviral defense.

Interestingly, miR159 and tasi255 levels were unaffected in *lpeat1*, *lpeat2*, and *lpeat1 lpeat2* compared with Col-0, suggesting a specific function of LPEAT2 in antiviral defense (Figure 2F and 2G). To confirm this possibility, we generated a homozygous plant by crossing *lpeat2* with a transgenic *SUC-SUL* plant in which a small RNA derived from the *SULPHUR* (*SUL)* transgene suppresses endogenous *SULPHUR* expression in phloem companion cells to cause leaf vein yellowing. There were no significant differences in the degree of leaf vein yellowing or the expression levels of *SULPHUR* mRNA between *lpeat2 SUC-SUL* plants and *SUC-SUL* lines (Supplemental Figure 3A and 3B), confirming that LPEAT2 specifically regulates plant antiviral RNAi.

LPEAT2 modulates RDR1/6-mediated antiviral RNAi

The majority of vsiRNAs produced after CMV-Δ2b or CMV-2aTΔ2b infection are secondary vsiRNAs dependent on RDR1/6 (Wang et al., 2010). CMV-Δ2b infection mainly stimulates RDR6-mediated vsiRNA amplification, whereas CMV-2aTΔ2b infection mainly induces RDR1-mediated vsiRNA biogenesis (Wang et al., 2011; Liu et al., 2022). To determine whether LPEAT2 regulates RDR1/6-mediated vsiRNA production, we crossed *avi4* with *rdr1*, *rdr6*, and *rdr1 rdr6* to generate *rdr1 lpeat2*, *rdr6 lpeat2*, and *rdr1 rdr6 lpeat2*, then infected these mutants with CMV-Δ2b or CMV-2aTΔ2b.

Like *rdr1/6,* the *rdr6 lpeat2* double mutant showed further reductions in vsiRNA production and a lower vsiRNA/viral RNA ratio compared with *rdr6* upon infection with CMV-Δ2b (Figure 3D and 3E; Supplemental Figure 12), suggesting that LPEAT2 functions independent of RDR6. However, the *rdr1 lpeat2* mutant exhibited severe disease symptoms and increased viral accumulation, resembling the single *lpeat2* mutant (Figure 3A–3E), indicating that LPEAT2 probably functions in RDR1-mediated defense. Consistent with these observations, the *rdr1 rdr6 lpeat2* triple mutant showed phenotypes comparable to those of *rdr1 rdr6* (Figure 3A–3E and Supplemental Figure 12). These results indicate that LPEAT2 functions mainly in RDR1-mediated antiviral RNAi upon CMV-Δ2b infection.

Upon infection with CMV-2aTΔ2b, the *rdr1 lpeat2* double mutant exhibited increased levels of viral CP and genomic RNA, together with a further reduction in vsiRNA levels compared with *rdr1* alone, mirroring the *rdr1 rdr6* phenotype (Figure 3B–3D and Supplemental Figure 12). However, the *rdr6 lpeat2* mutant showed vsiRNA production and disease symptoms similar to those of the single *lpeat2* mutant (Figure 3A–3E). Furthermore, the accumulation of viral CP, genomic RNA, and vsiRNAs in the *rdr1 rdr6 lpeat2 triple mutant* was comparable to those in the *rdr1 rdr6* mutant (Figure 3 and Supplemental Figure 12). These results indicate that LPEAT2 functions in RDR1/6-mediated antiviral defense upon CMV-2aTΔ2b infection but has a more pronounced role in the RDR6 pathway.

LPEAT2 interacts with AVI2 to regulate vsiRNA biogenesis

ALA1, ALA2 (AVI1), and AVI2, three novel regulators of antiviral RNAi, were recently characterized as key components of RDR1/6-mediated vsiRNA production in *Arabidopsis* (Guo et al., 2017, 2018). To determine whether LPEAT2 functions together with these components, we generated homozygous *ala1 lpeat2*, *ala2 lpeat2*, and *avi2 lpeat2* double mutants by genetic crossing. After infection with CMV-Δ2b or CMV-2aTΔ2b, both the *ala1 lpeat2* and *ala2 lpeat2* double mutants exhibited more severe disease symptoms, significantly higher viral CP and genomic RNA levels (Supplemental Figure 4A–4C), and a marked reduction in vsiRNA levels and the vsiRNA/viral RNA ratio (Supplemental Figure 4D and 4E) compared with their respective parental mutants. These results indicate that LPEAT2 functions independent of ALA1/2 in antiviral immunity.

By contrast, the *avi2 lpeat2* double mutant did not differ significantly from the *avi2 and lpeat2 single mutants* in viral CP and genomic RNA accumulation, vsiRNA levels, or the vsiRNA/viral RNA ratio after CMV-Δ2b or CMV-2aTΔ2b infection (Figure 4B–4E and Supplemental Figure 13). This result indicates that LPEAT2 collaborates with AVI2 to enhance RDR1/6-dependent vsiRNA biogenesis in *Arabidopsis*. Surprisingly, the *avi2 lpeat2* double mutant exhibited more severe disease symptoms than the parental mutants (Figure 4A), suggesting that LPEAT2 may also participate in additional pathways to modulate disease symptoms.

The genetic interaction between LPEAT2 and AVI2 prompted us to investigate whether LPEAT2 physically interacts with AVI2. Reciprocal yeast two-hybrid (Y2H) analysis revealed that LPEAT2 interacted with AVI2, but not with the empty vector control (pGAD-T7), when expressed in yeast cells (Figure 4F). Co-immunoprecipitation (co-IP) assays confirmed the interaction between LPEAT2 and AVI2 in plant cells, with Myc-GFP serving as a negative control (Figure 4G and Supplemental Figure 5A).

(C and D) Northern blot analyses of viral genomic RNA or vsiRNA accumulation in non-inoculated systemic leaves shown in Figure 2D and 2E.
(E) Ratios of vsiRNA/genomic RNA were calculated from the northern hybridization signals in **(C)** and **(D)**, with the ratio in Col-0 normalized to 1.
The experiments were repeated independently three times with similar results. Data are presented as means ± SEM from three replicates; letters indicate significant differences among groups (one-way ANOVA, Duncan's test, $p < 0.05$), and dots represent individual values.

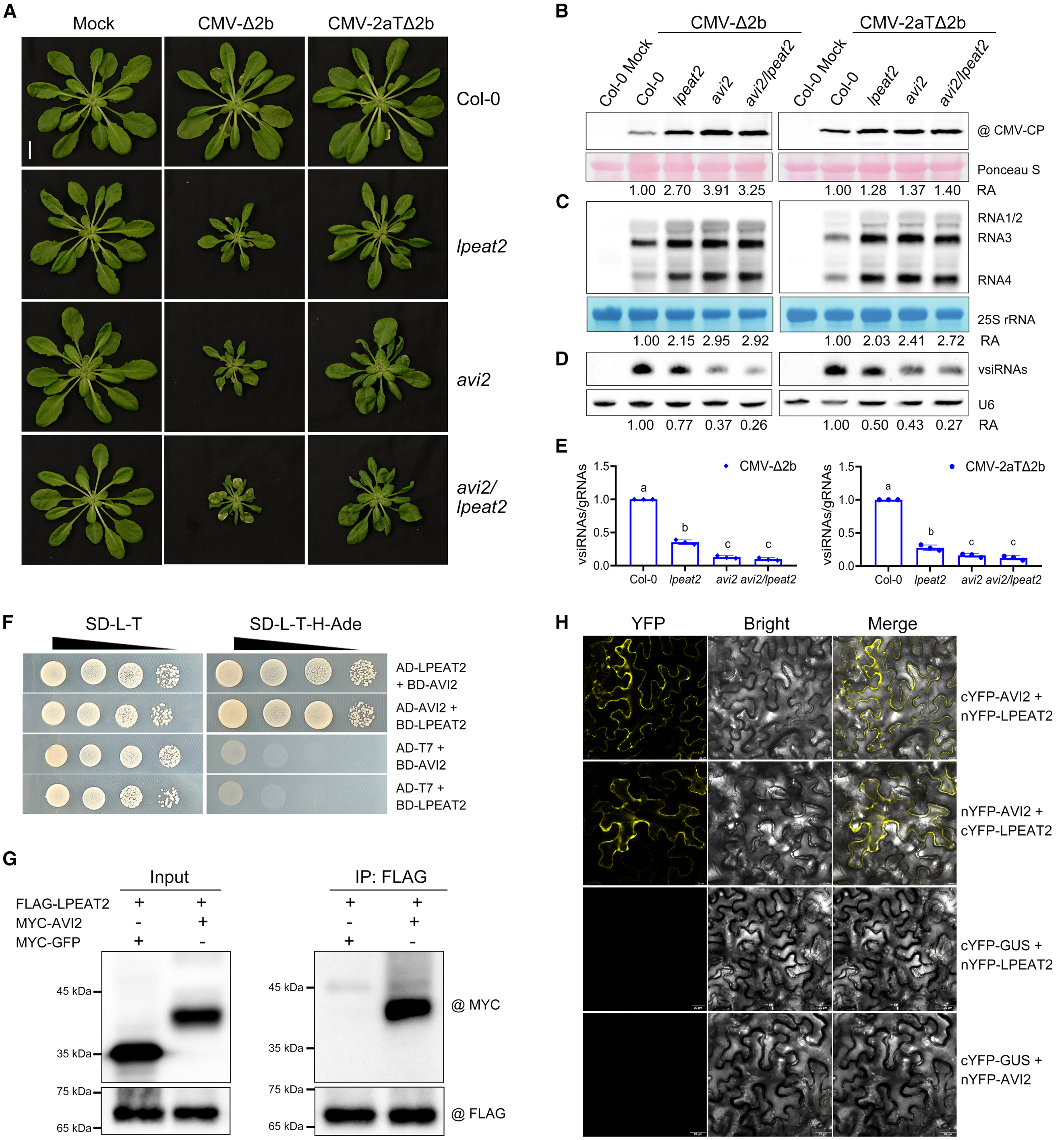

Figure 4. LPEAT2 interacts with AVI2 to regulate vsiRNA biogenesis.

(A) Plants photographed 14 days after inoculation with CMV-Δ2b, CMV-2aTΔ2b, or mock (buffer). Scale bar, 1 cm.

(B) Western blot analyses detecting the accumulation of virus coat protein in different homozygous mutants as shown in Figure 2C.

(C and D) Northern blot analyses of viral genomic RNA or vsiRNA accumulation in non-inoculated systemic leaves. 25S rRNA staining or a U6 RNA probe was used as a loading control. Relative accumulation (RA) of viral genomic RNA or vsiRNA was quantified using ImageJ by normalizing to 25S rRNA or U6, respectively.

(E) Ratios of vsiRNA/genomic RNA were calculated from the northern hybridization signals in **(C)** and **(D)**, with the ratio in Col-0 normalized to 1. The experiments were repeated independently three times with similar results. Data presented are means ± SEM from three replicates; letters indicate significant differences among groups (one-way ANOVA, Duncan's test, $p < 0.05$), and dots represent individual values.

(legend continued on next page)

Bimolecular fluorescence complementation (BiFC) assays demonstrated that LPEAT2 interacted with AVI2 but not with β-glucuronidase (the negative control) in plant cells (Figure 4H). Together, these analyses indicate that LPEAT2 directly interacts with AVI2 to regulate vsiRNA biogenesis.

LPEAT2 interacts with and stabilizes ATG8a

LPEAT2 has been reported to maintain the accumulation of the ATG8 complex, a key component of the autophagic machinery in *Arabidopsis* (Jasieniecka-Gazarkiewicz et al., 2021). Among the nine members of the ATG8 family (ATG8a–ATG8i), LPEAT2 specifically interacted only with ATG8a in Y2H assays (Figure 5C), and both co-IP and BiFC assays confirmed the LPEAT2–ATG8a interaction in plant cells (Figure 5A and 5B; Supplemental Figure 5B).

To determine whether LPEAT2 contributes to the stabilization of ATG8a, we obtained GFP-tagged ATG8a driven by the cauliflower mosaic virus (CaMV) 35S promoter and transformed it into Col-0 plants. The resulting GFP-ATG8a/Col-0 transgenic plants were then crossed with *lpeat1*, *lpeat2*, and *lpeat1 lpeat2* mutants to generate corresponding GFP-ATG8a *lpeat1*, GFP-ATG8a *lpeat2*, and GFP-ATG8a *lpeat1 lpeat2* plants. Confocal microscopy revealed that GFP-ATG8a accumulated abundantly in both GFP-ATG8a/Col and GFP-ATG8a *lpeat1* plants, but its accumulation was drastically reduced in GFP-ATG8a *lpeat2* and GFP-ATG8a *lpeat1 lpeat2* plants (Figure 5D and 5E). Consistent with these findings, western blot analyses showed much higher accumulation of GFP-ATG8a in GFP-ATG8a/Col-0 and GFP-ATG8a *lpeat1* plants but markedly reduced accumulation in GFP-ATG8a *lpeat2* and GFP-ATG8a *lpeat1 lpeat2* plants (Figure 5F). Interestingly, the acyltransferase activity of LPEAT2 is critical for this function, as deletion of the PlsC domain or point mutation of conserved amino acids in this domain impaired the function of LPEAT2 in stabilizing GFP-ATG8a (Supplemental Figure 6). These results strongly suggest that LPEAT2 interacts with and stabilizes ATG8a, which likely contributes to the modulation of the autophagy pathway.

LPEAT2 enhances ATG8a-mediated autophagy

To assess whether LPEAT2 modulates ATG8a-mediated autophagy, we examined the accumulation of autophagosomes labeled with GFP-ATG8a in plant roots following treatment with concanamycin A (ConA) under carbon-starvation conditions, a method routinely used to induce autophagosome formation. Substantial induction of autophagosome formation was observed in GFP-ATG8a/Col-0 and GFP-ATG8a *lpeat1* plants (Figure 6A–6C). However, significantly fewer autophagosomes were present in GFP-ATG8a *lpeat2* and GFP-ATG8a *lpeat1 lpeat2* plants, and this was accompanied by a dramatic reduction in GFP-ATG8a protein levels in these plants, but not in GFP-ATG8a *lpeat1* plants, compared with GFP-ATG8a/Col-0 (Figure 6A–6C). These results indicate that LPEAT2 contributes to facilitating autophagosome formation.

Interestingly, like the *atg5-1* mutant, *lpeat2* exhibited autophagy-deficient phenotypes under dark-induced senescence (Supplemental Figure 7C), whereas *lpeat1* did not (Supplemental Figure 7A and 7B). However, unlike *atg5-1*, *lpeat2* did not show autophagy-deficient phenotypes under conditions of carbon or nitrogen starvation (Supplemental Figure 7A and 7B), suggesting that LPEAT2 regulates autophagy responses only under specific environmental stresses.

We next examined the accumulation of GFP-ATG8a or endogenous ATG8 proteins in non-inoculated systemic leaves before and after virus infection. Western blot analysis revealed significantly reduced levels of both the endogenous ATG8 complex (Figure 6D) and transgenic GFP-ATG8a (Supplemental Figure 8) in *lpeat2* and *lpeat1 lpeat2* mutants compared with Col-0 plants, both before and after infection with CMV-Δ2b or CMV-2aTΔ2b. We then assessed the protein levels of NBR1, an autophagy cargo receptor that is used to monitor autophagic flux (Rasmussen et al., 2022). Accumulation of NBR1 was significantly higher in *lpeat2* and *lpeat1 lpeat2* mutants than in Col-0 before and after infection with CMV-Δ2b or CMV-2aTΔ2b (Figure 6D). By contrast, ATG8 levels were substantially higher and NBR1 levels were markedly lower in LPEAT2-overexpressing transgenic plants compared with Col-0 before and after viral infection (Figure 6E). Together, these findings demonstrate that LPEAT2 plays critical roles in autophagy, particularly in enhancing ATG8a-mediated autophagy upon viral infection.

LPEAT2 modulates ATG8a-mediated antiviral defense

Autophagy is known to play a critical role in the antiviral defense of *Arabidopsis* (Yang and Liu, 2022; Sharma et al., 2024). To investigate whether LPEAT2 modulates antiviral immunity through ATG8a-mediated autophagy, we next examined the phenotypic and molecular responses of *atg8a*, *atg8b*, *lpeat2*, and GFP-ATG8a transgenic plants to infection with CMV-Δ2b or CMV-2aTΔ2b. The single mutants of *atg8a* or *atg8b* did not differ significantly from Col-0 in disease symptoms or accumulation of viral CP, viral genomic RNA, or vsiRNAs, probably because of functional redundancy with other members of the ATG8 family (Supplemental Figure 9).

By contrast, infection of GFP-ATG8a/Col-0 plants with either CMV-Δ2b or CMV-2aTΔ2b did not induce disease symptoms, and viral CP and genomic RNA accumulation was significantly reduced compared with that of Col-0 plants (Figure 7A–7C). These results indicate that overexpression of GFP-ATG8a enhances resistance to both viral strains. However, GFP-ATG8a *lpeat2* plants developed disease symptoms similar to those observed in *lpeat2* mutants (Figure 7A), and the accumulation of viral CP and genomic RNA in these plants was comparable to that in *lpeat2* plants after viral infection (Figure 7B and 7C), indicating that LPEAT2 is required for ATG8a-mediated antiviral defense.

(F) A Y2H assay was performed to examine the interaction between LPEAT2 and AVI2 proteins.

(G) Co-IP analyses comparing LPEAT2 and AVI2 proteins 48 h post infiltration (hpi) after transient expression in *N. benthamiana* leaves.

(H) BiFC analysis was performed to examine the interaction between LPEAT2 and AVI2 in *N. benthamiana* leaves. Fluorescence signals were imaged at 48 hpi under a confocal microscope. Scale bar, 20 μm.

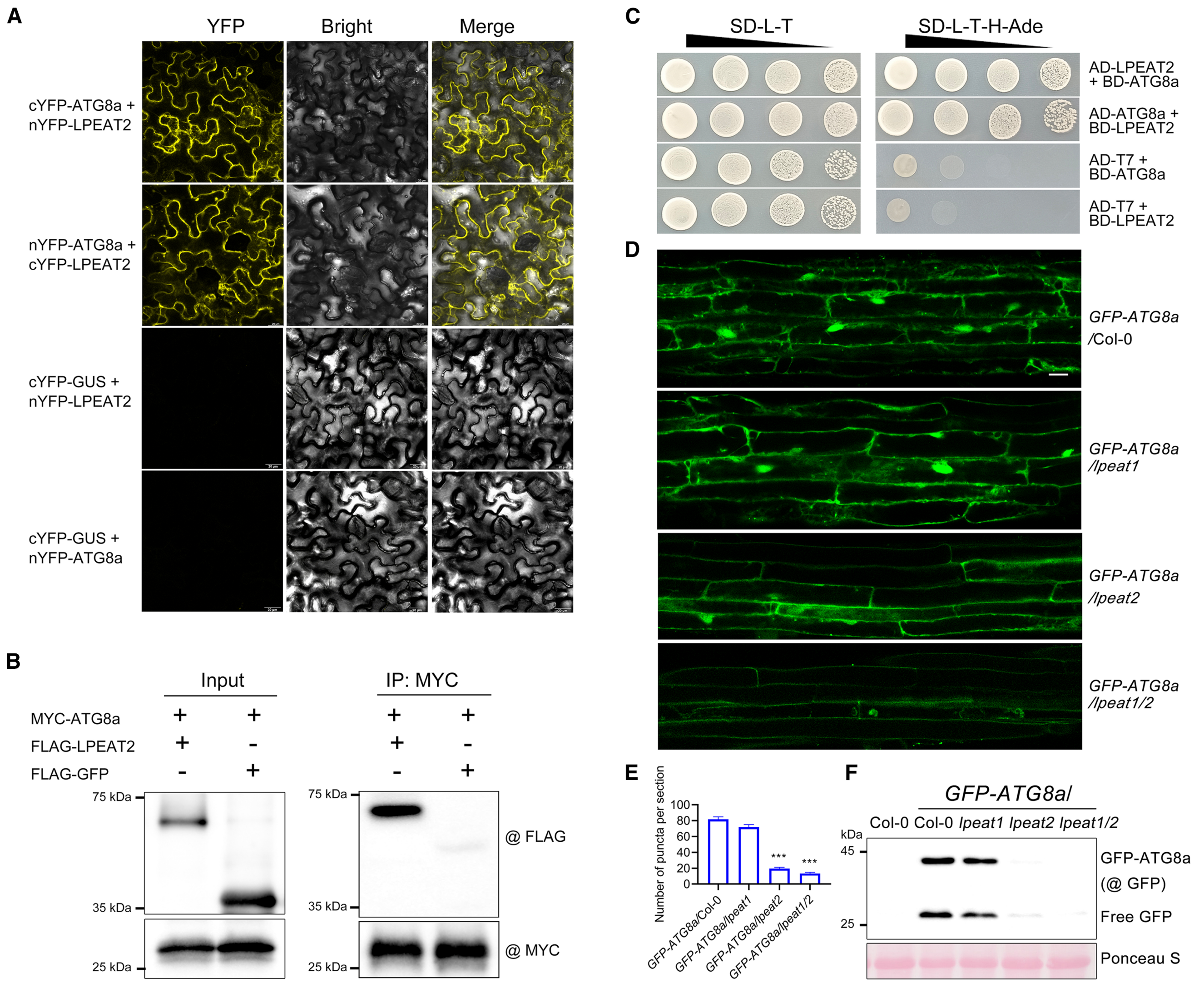

Figure 5. LPEAT2 interacts with ATG8a to maintain ATG8a accumulation.

(A) BiFC analysis was performed to investigate the interaction between LPEAT2 and ATG8a in *N. benthamiana* leaves. Fluorescence signals were captured at 48 hpi under a confocal microscope. Scale bar, 20 μm.

(B) Co-IP analysis comparing LPEAT2 and ATG8a proteins at 48 hpi after transient expression in *N. benthamiana* leaves.

(C) Y2H assay detecting the interaction between LPEAT2 and ATG8a proteins. Various vector combinations were transformed into yeast cells, and protein interactions were assessed based on colony growth on SD/−Leu−Trp and SD/−Leu−Trp−His−Ade selective medium.

(D) Detection of GFP-ATG8a-labeled autophagosome bodies in root cells by confocal microscopy. Seven-day-old seedlings were grown on 1/2 MS plates without sucrose in darkness. Scale bar, 20 μm.

(E) Number of autophagosome bodies per section in root cells. Data represent the average values calculated from three biological replicates. Error bars indicate standard deviation; ***$p < 0.001$ (Student's *t*-test).

(F) Levels of GFP-ATG8a and free GFP proteins were detected in plants by western blotting with anti-GFP antibodies **(D)**; Ponceau S staining of Rubisco served as a loading control.

Interestingly, whereas vsiRNA production was elevated in GFP-ATG8a/Col plants, accompanied by a significant reduction in viral RNA, accumulation of vsiRNA was markedly reduced in GFP-ATG8a *lpeat2* plants and was accompanied by increased accumulation of viral genomic RNA (Figure 7D and Supplemental Figure 14). The vsiRNA/viral RNA ratio was significantly higher in GFP-ATG8a/Col plants but was notably reduced in GFP-ATG8a *lpeat2* plants, consistent with that in *lpeat2* mutants (Figure 7E). These findings demonstrate that LPEAT2 functions as a key regulator of ATG8a-mediated antiviral defense, probably by controlling vsiRNA production and virus accumulation.

VSR 2b represses the AVI4- and ATG8a-dependent antiviral response

CMV 2b protein is one of the first identified VSRs and has been shown to suppress antiviral RNAi by inhibiting the biogenesis of vsiRNAs and the activities of effector AGOs (Ding et al., 1995; Diaz-Pendon et al., 2007; Duan et al., 2012). To determine

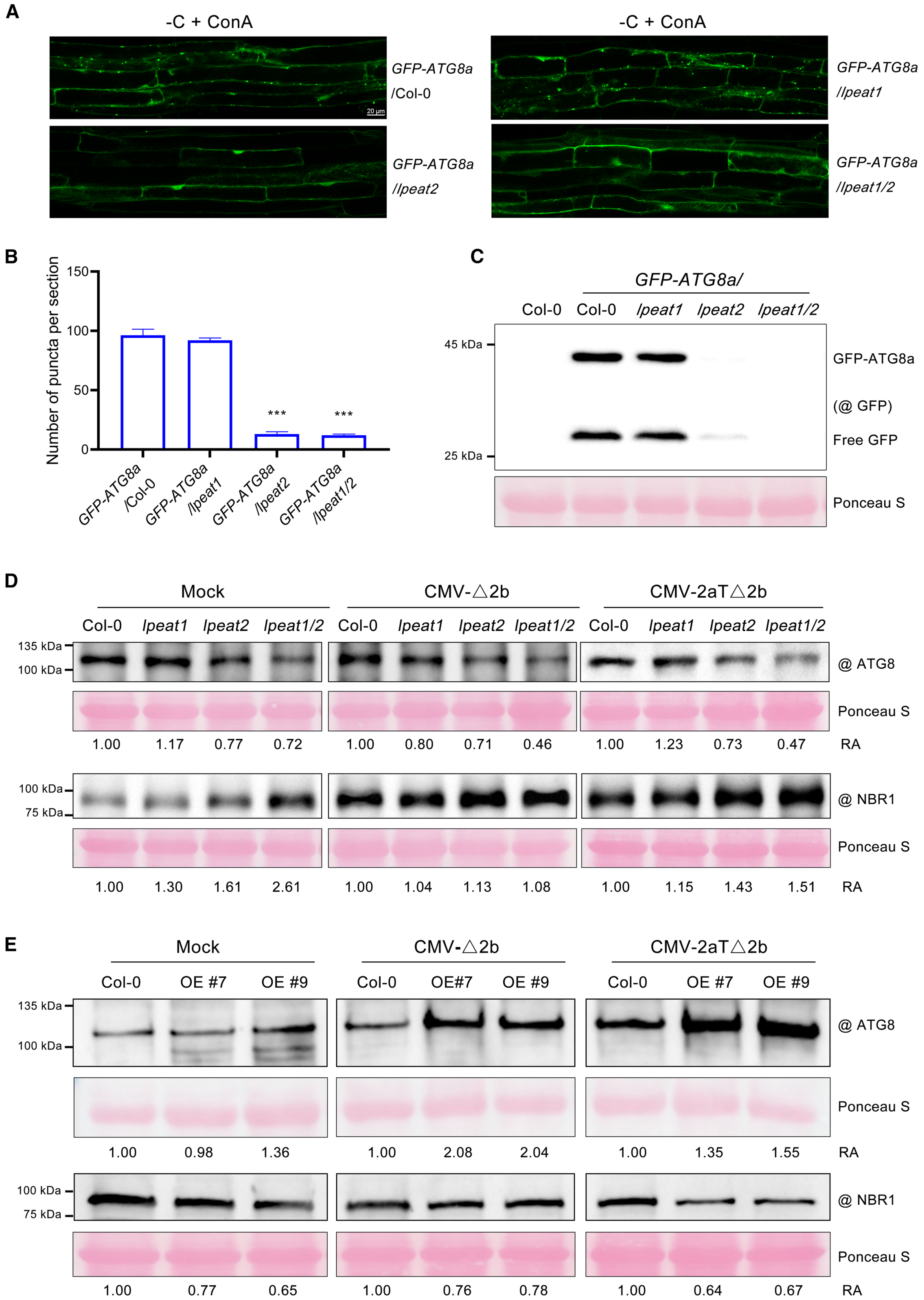

Figure 6. LPEAT2 enhances ATG8a-mediated autophagy.
(A) Detection of GFP-ATG8a-labeled autophagosome bodies in root cells by confocal microscopy. Seven-day-old seedlings grown on 1/2 MS plates were transferred to MS−C liquid medium containing 1 μM ConA and incubated in darkness for 12 h. Scale bar, 20 μm.

(legend continued on next page)

whether 2b represses the AVI4-dependent antiviral response, we infected Col-0, *lpeat2*, GFP-ATG8a, and GFP-ATG8a *lpeat2 plants* with wild-type Fny-CMV, in which 2b is intact. Owing to the potent suppressive function of 2b, all of these plants developed severe disease symptoms after Fny-CMV infection (Supplemental Figure 10), which was consistent with previous reports (Diaz-Pendon et al., 2007; Guo et al., 2017). Notably, northern blot analyses showed that viral RNA and vsiRNAs accumulated to the same levels in Col-0, *lpeat2*, GFP-ATG8a, and GFP-ATG8a *lpeat2* plants after Fny-CMV infection (Figure 7F and 7G). Statistical analysis based on three independent experiments confirmed that the accumulation of viral RNA and vsiRNAs did not differ significantly among Col-0, *lpeat2*, GFP-ATG8a, and GFP-ATG8a *lpeat2* plants after Fny-CMV infection (Figure 7H).

These results indicate that the vsiRNA biogenesis and antiviral defense mediated by AVI4 and ATG8a could be repressed by 2b, a well-known VSR. However, from another perspective, these results provide evidence for the critical function of AVI4 in regulating small-RNA-based antiviral immunity, although its role is constrained by wild-type viruses that have evolved potent VSRs during the virus–host evolutionary arms race.

DISCUSSION

We identified LPEAT2 (AVI4) as a novel regulator of antiviral RNAi through an unbiased forward genetic screen (Figure 1 and Supplemental Figure 1). We demonstrated that LPEAT2 is induced by viral infection and plays a vital role in enhancing RDR1/6-dependent production of vsiRNAs while not affecting endogenous small-RNA pathways (Figures 2, 3, and 4). Genetic and biochemical analyses revealed that LPEAT2 directly interacts with AVI2 to facilitate RDR1/6-mediated vsiRNA biogenesis. In addition, we demonstrated that LPEAT2 interacts with and stabilized ATG8a, promoting autophagosome formation and host resistance to viral infection (Figures 5 and 6). Notably, LPEAT2 mutation compromised the antiviral resistance conferred by ATG8a overexpression, a process that was strongly associated with vsiRNA production (Figure 7A–7E), and VSR 2b repressed AVI4- and ATG8a-mediated antiviral responses (Figure 7F and 7G; Supplemental Figure 10). Taken together, we propose that LPEAT2 cooperates with AVI2 and ATG8a to promote vsiRNA production for robust small-RNA-based antiviral defense in *Arabidopsis* (Figure 7I).

LPEAT2, a lysophospholipid acyltransferase, plays a major role in catalyzing the biosynthesis of phospholipids, particularly PE, in *Arabidopsis* (Stålberg et al., 2009; Jasieniecka-Gazarkiewicz et al., 2016, 2017). PE is one of the primary phospholipids in the lipid bilayer of cellular membranes (Yu et al., 2021). PE, together with another anionic phospholipid, PS, is exclusively distributed in the outer leaflet of the lipid bilayer in cell membranes, facilitating formation of the classical cellular-membrane structure characterized by the asymmetrical distribution of specific phospholipids (Vance and Tasseva, 2013; van der Veen et al., 2017). This asymmetrical membrane structure may contribute to the formation of membrane microdomains involved in various physiological and biochemical processes, including biotic and abiotic stress responses (Yu et al., 2021). Interestingly, in our previous studies, we identified AVI2, AVI1 (ALA2), and ALA1 as novel regulators that enhance vsiRNA biogenesis to promote antiviral immunity, probably by remodeling the cell membrane structure (Guo et al., 2017, 2018). Here, we discovered that LPEAT2 (AVI4) directly interacts with AVI2 to regulate RDR1/6-mediated vsiRNA biogenesis. Together, our findings reveal that a complex may be formed by these components to efficiently produce v-siRNAs for small-RNA-based antiviral defense, probably by remodeling the cellular membrane structure.

LPEAT2 has been reported to positively modulate the accumulation of the ATG8 complex in *Arabidopsis* (Jasieniecka-Gazarkiewicz et al., 2021). For ATG8, a core component of the autophagic machinery, conjugation with PE constitutes a crucial step in autophagosome formation (Yu et al., 2018; Nakatogawa 2020; Zhuang et al., 2024). We demonstrated that LPEAT2 specifically interacts with and stabilized ATG8a and promoted autophagosome formation. Modulation of antiviral defense by the autophagy pathway is well documented in plants (Yang et al., 2022; Sharma et al., 2024). Interestingly, we found that overexpression of ATG8a promotes plant antiviral resistance, and the induced antiviral response is abrogated after LPEAT2 mutation (Figure 7A–7E). Therefore, LPEAT2 also functions to promote autophagy-mediated antiviral resistance in plants, probably by facilitating autophagosome formation.

An interesting observation in this study is that the antiviral resistance of GFP-ATG8a transgenic plants is strongly associated with vsiRNA accumulation, whereas the compromised antiviral resistance of GFP-ATG8a *lpeat2* plants is associated with reduced vsiRNA accumulation. These findings indicate that ATG8a-mediated autophagy may facilitate vsiRNA production and thereby promote antiviral RNAi in *Arabidopsis*. The autophagy pathway plays vital roles in modulating antiviral RNAi in plants (Li et al., 2025). A selective autophagy pathway has been reported to modulate antiviral RNAi by mediating the degradation of positive or negative regulators in the antiviral pathway (Tong et al., 2023). Selective autophagy may also contribute to the supply of materials or energy essential for vsiRNA biogenesis, thereby facilitating efficient antiviral RNAi (Masclaux-Daubresse et al., 2017; Marshall and Vierstra, 2018;

(B) Number of autophagosome bodies per section in root cells. Data represent the average values calculated from three biological replicates. Error bars indicate standard deviation; $^{***}p < 0.001$ (Student's *t*-test).

(C) Levels of GFP-ATG8a and free GFP proteins were assessed in plants by western blotting with GFP-specific antibodies; Ponceau S staining served as a loading control.

(D) Western blot analysis was performed to assess accumulation of the ATG8a–i complex and NBR1 in plants following mock treatment or virus infection. Non-denatured total proteins were subjected to western blotting using specific antibodies against ATG8 and NBR1; Ponceau S staining served as a loading control. Relative accumulation (RA) of proteins was quantified from western blot signals relative to the loading control using ImageJ, with the ratio in Col-0 normalized to 1.

(E) The ATG8a–i complex and NBR1 were detected in LPEAT2-overexpressing plants using western blot analysis following the method described in **(D)**.

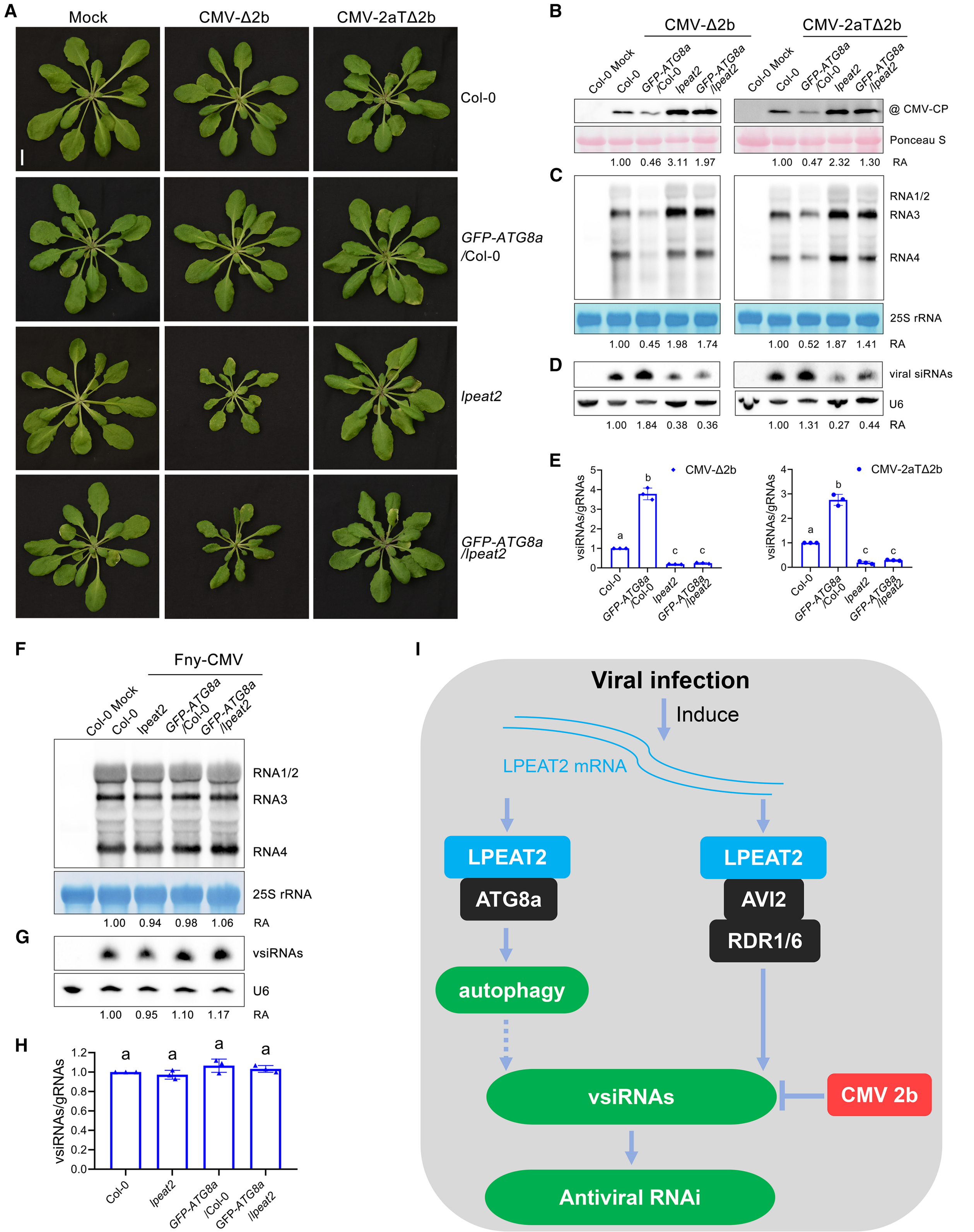

Figure 7. LPEAT2 controls ATG8a-mediated antiviral defense by enhancing vsiRNA production.

(A) Plants photographed 14 days after inoculation with CMV-Δ2b, CMV-2aTΔ2b, or mock (buffer). Scale bar, 1 cm.

(B) Western blot analysis detecting the accumulation of virus coat protein in different plants, with Rubisco in the same membrane stained with Ponceau S to show equal loading. Relative accumulation (RA) of coat protein was calculated using ImageJ.

(legend continued on next page)

Gouguet and Üstün, 2022). It will be interesting to determine whether AVI4 promotes ATG8a-mediated autophagy to enhance AVI2-mediated vsiRNA biogenesis.

Overall, our findings highlight the intricate interplay among a plant metabolic enzyme, antiviral defense mechanisms, and virus counteraction. AVI4 (LPEAT2) exemplifies how metabolic enzymes can integrate disparate pathways to reinforce small-RNA-based antiviral immunity. Future studies will illuminate how phospholipid remodeling through AVI4 and other AVIs coordinates antiviral RNAi and autophagy, potentially offering insights into strategies for engineering crops with enhanced resistance to viral diseases.

METHODS

Plant materials and virus inoculation

Arabidopsis lpeat2 (*avi4* and *Salk_073928*), *lpeat1* (*Salk_016798*), *atg8a* (*Salk_045344*), and *atg8b* (*Salk_036033*) were obtained from the Arabidopsis Biological Resource Center. Transgenic plants expressing *GFP-ATG8a* were obtained from the Nottingham Arabidopsis Stock Center. Homozygous mutants for *rdr1-1*, *rdr6-15*, and *rdr1-1 rdr6-15* were described previously (Wang et al., 2010). The *ala1* and *ala2-1* mutants were characterized in Guo et al. (2017) and the *avi2* mutant in Guo et al. (2018); the *SUC-SUL* transgenic line was kindly provided by Professor Shou-Wei Ding at the University of California-Riverside. Seeds were vernalized for at least 2 days at 4°C, and seedlings were grown under an 8-h/16-h light/dark cycle at 23°C in a controlled growth room. *Arabidopsis* plants with approximately 7–8 leaves were used for virus inoculation as described previously (Guo et al., 2017). The mutant viruses CMV-Δ2b and CMV-2aTΔ2b were derived from the subgroup I strain Fny-CMV (Guo et al., 2017).

Mapping by sequencing

The F2 population generated after backcrossing *avi4* with Col-0 was infected with CMV-Δ2b, and approximately 50 symptomatic and asymptomatic plants were separately pooled for genomic DNA extraction. DNA libraries were constructed according to the manual of the NEXTFLEX PCR-Free DNA sequencing kit (Bioo Scientific, USA). The two DNA libraries were sequenced, and the reads were mapped to the *Arabidopsis* genome for identification of the candidate gene as described previously (Guo et al., 2017).

Development of *LPEAT2* transgenic *Arabidopsis*

LPEAT2 was amplified from Col-0 cDNA using Q5 High-Fidelity DNA Polymerase (New England Biolabs, USA) and cloned into the *pCAMBIA1300* vector by in-fusion cloning. The resulting *pCAMBIA1300-LPEAT2* plasmid was transformed into Col-0 plants for overexpression, and the native promoter-driven *pGWB10-LPEAT2* construct was transformed into *avi4* for complementation analysis. Transformations were performed using the *Agrobacterium*-mediated floral dip method to generate transgenic plants. Primers are listed in Supplemental Table 1.

Northern blot analysis

RNA was extracted from the upper systemic leaves of plants that had received virus or mock infection treatment. Leaves from ~10 individual plants were pooled for each biological replicate. High- and low-molecular-weight RNA were isolated using the hot phenol method. Northern blotting was performed as described previously (Guo et al., 2017) using biotin-labeled DNA probes with biotin-11-dUTP (Thermo Scientific, USA), and signals were detected using a chemiluminescent Nucleic Acid Detection Module Kit (Thermo Scientific). The probes used to detect viral genomic RNA, vsiRNAs, tasiRNA255, miR159, and U6 were described previously (Guo et al., 2017).

Western blot analysis

Plant proteins were extracted using a buffer (20 mM Tris–HCl [pH 7.5], 5 mM $MgCl_2$, 150 mM NaCl, 5 mM dithiothreitol [DTT], and 2% β-mercaptoethanol) at a ratio of 1:2 (w/v). Proteins were separated by SDS–PAGE and transferred onto nitrocellulose membranes. GFP antibodies (TransGen, China) and CMV CP antibody were diluted at 1:5000, and a peroxidase-conjugated goat anti-rabbit immunoglobulin G (IgG) secondary antibody (TransGen, China) was used at a dilution of 1:10 000. For detection of ATG8a–i complex and NBR1 proteins, a native protein extraction buffer (50 mM Tris–MES [2-(*N-morpholino)ethanesulfonic acid]* [pH 8.0], 0.5 M sucrose, 1 mM $MgCl_2$, 10 mM EDTA, 5 mM DTT, and Roche protease inhibitor) was used, and the ATG8 (#ab98830, Abcam, UK) and NBR1-IgG (Agrisera, Sweden) antibodies were diluted at 1:3000 and used as primary antibodies for western blotting.

Confocal microscopy of autophagosomes

Surface-sterilized seeds were sown on plates with half-strength Murashige and Skoog medium (1/2 MS), vernalized at 4°C for 2 days, and transferred to a long-day photoperiod for 7 days. The seedlings were then placed in MS liquid medium without sucrose (carbon starvation, −C) or without nitrogen (nitrogen starvation, −N), with or without 1 μM ConA (Aladdin), and incubated in darkness for 12 h. Autophagosomes labeled with GFP-ATG8a in plant cells of the maturation zone of the primary root were observed using a Leica TCS SP8 confocal microscope; GFP was imaged with 488-nm excitation and a detection window of 500–530 nm.

Y2H assay

The coding sequences of the target genes were cloned into the pGBKT7 and pGADT7 vectors to investigate protein–protein interactions using the Matchmaker Gold Yeast Two-Hybrid System (Clontech, CA, USA). The pGADT7-T plasmid was used as a negative control, and the primers are listed in Supplemental Table 1. The prey and bait plasmids were co-transformed into the *AH109* yeast strain. Yeast cultures were serially diluted at ratios of 1:1, 1:10, 1:100, and 1:1000 before plating; β-galactosidase activity was measured by plating the yeast on synthetic

(C and D) Northern blot analyses of viral genomic RNA or vsiRNA accumulation in non-inoculated systemic leaves. 25S rRNA staining or a U6 RNA probe was used as a loading control. Relative accumulation (RA) of viral genomic RNA or vsiRNA was quantified using ImageJ by normalizing to 25S rRNA or U6, respectively.

(E) Ratios of vsiRNA/genomic RNA were calculated from the northern hybridization signals in **(C)** and **(D)**, with the ratio in Col-0 normalized to 1. The experiments were repeated independently three times with similar results. Data presented are means ± SEM from three replicates; letters indicate significant differences among groups (one-way ANOVA, Duncan's test, $p < 0.05$), and dots represent individual values.

(F and G) Northern blot analyses of viral genomic RNA or vsiRNA accumulation in non-inoculated systemic leaves using the method described for **(C)** and **(D)**.

(H) Ratios of vsiRNA/genomic RNA were calculated from the northern hybridization signals in **(F)** and **(G)** using the method described for **(E)**.

(I) A working model showing how LPEAT2 promotes antiviral RNAi and autophagy to enhance antiviral immunity in *Arabidopsis*. LPEAT2 is induced upon viral infection. It interacts with AVI2 to enhance the biogenesis of RDR1/6-dependent vsiRNAs. It also interacts with and stabilizes ATG8a to enhance autophagosome formation, contributing to ATG8a-mediated antiviral defense, at least in part, by enhancing vsiRNA production. On the other hand, 2b suppresses the antiviral response mediated by AVI4 and ATG8a by repressing small-RNA-mediated antiviral RNAi.

defined (SD)/−Leu−Trp and SD/−Leu−Trp−His−Ade selection media at 30°C.

In vivo co-IP assay

Plasmids with MYC tags or FLAG tags were co-expressed in *Nicotiana benthamiana* leaves, and a GFP-FLAG plasmid was used as a negative control. After 48 h of infiltration, the leaf tissues were harvested, and proteins were extracted by homogenizing the tissue in IP lysis buffer (Thermo Scientific, #87788) containing 10 mM DTT and a 1× EDTA-free protease inhibitor cocktail (Roche, Switzerland). The mixture was incubated on ice for 30 min, followed by centrifugation at 12 000 *g* for 10 min at 4°C. The protein lysate was then incubated with 20 μl of ChromoTek Myc-Trap magnetic agarose (Proteintech, USA) or monoRab anti-FLAG magnetic beads (Genscript, China) for 2 h at 4°C with gentle shaking. After co-incubation, the immunoprecipitates were washed three times with 1× PBS and resuspended in 100 μl of 2× SDS–PAGE sample buffer (10% SDS, 500 mM Tris–HCl, 50% glycerol, 1% bromophenol blue, and 2% β-mercaptoethanol; pH 6.8). Proteins were detected by western blotting using anti-FLAG and anti-MYC antibodies at a 1:5000 dilution (TransGen, China). The primers used in the experiments are listed in Supplemental Table 1.

BiFC assay

Genes were amplified via PCR and cloned into the pDONR221 vector. The inserts were then recombined into the pEarleyGate-nYFP and pEarleyGate-cYFP vectors through an LR reaction. These recombinant plasmids were introduced into *Agrobacterium tumefaciens* strain GV3101 by electroporation and co-infiltrated into *N. benthamiana* leaves. The reconstituted YFP fluorescence signal was detected with 514-nm excitation and a detection window of 525–550 nm at 48 h post infiltration using a Leica TCS SP8 confocal microscope. The primers used in the experiments are listed in Supplemental Table 1.

DATA AND CODE AVAILABILITY

All data are available in the main text or supplemental information.

FUNDING

This research was supported by Fujian Hundred-Talents Plan, China and a start-up fund from Shandong Agricultural University, China.

ACKNOWLEDGMENTS

We are grateful to Prof. Shou-Wei Ding at the University of California-Riverside for his advice regarding the research. No conflict of interest declared.

AUTHOR CONTRIBUTIONS

W.Y., Y.Z., and B.J. designed and performed the experiments, analyzed the data, and drafted the manuscript; J.N., S.J., and Z.Z. performed the experiments and analyzed the data; and Z.G. supervised the research and wrote the paper. All authors have read and approved the final manuscript.

SUPPLEMENTAL INFORMATION

Supplemental information is available at *Plant Communications Online*.

Received: September 10, 2025
Revised: February 15, 2026
Accepted: April 13, 2026
Published: April 14, 2026

REFERENCES

Baulcombe, D. (2004). RNA silencing in plants. Nature **431**:356–363. https://doi.org/10.1038/nature02874.

Brosseau, C., El Oirdi, M., Adurogbangba, A., Ma, X., and Moffett, P. (2016). Antiviral defense involves AGO4 in an arabidopsis-potexvirus interaction. Mol. Plant Microbe In. **29**:878–888. https://doi.org/10.1094/Mpmi-09-16-0188-R.

Cao, B., Ge, L., Zhang, M., Li, F., and Zhou, X. (2023). Geminiviral C2 proteins inhibit active autophagy to facilitate virus infection by impairing the interaction of ATG7 and ATG8. J. Integr. Plant Biol. **65**:1328–1343. https://doi.org/10.1111/jipb.13452.

Chen, X. (2009). Small RNAs and their roles in plant development. Annu. Rev. Cell Dev. Biol. **25**:21–44. https://doi.org/10.1146/annurev.cellbio.042308.113417.

Diaz-Pendon, J.A., Li, F., Li, W.X., and Ding, S.W. (2007). Suppression of antiviral silencing by cucumber mosaic virus 2b protein in is associated with drastically reduced accumulation of three classes of viral small interfering RNAs. Plant Cell **19**:2053–2063. https://doi.org/10.1105/tpc.106.047449.

Ding, S.W., Li, W.X., and Symons, R.H. (1995). A novel naturally-occurring hybrid gene encoded by a plant RNA virus facilitates long-distance virus movement. EMBO J. **14**:5762–5772. https://doi.org/10.1002/j.1460-2075.1995.tb00265.x.

Duan, C.G., Fang, Y.Y., Zhou, B.J., Zhao, J.H., Hou, W.N., Zhu, H., Ding, S.W., and Guo, H.S. (2012). Suppression of *Arabidopsis* ARGONAUTE1-mediated slicing, transgene-induced RNA silencing, and DNA methylation by distinct domains of the cucumber mosaic virus 2b protein. Plant Cell **24**:259–274. https://doi.org/10.1105/tpc.111.092718.

Gouguet, P., and Üstün, S. (2022). Crossing paths: recent insights in the interplay between autophagy and intracellular trafficking in plants. FEBS Lett. **596**:2305–2313. https://doi.org/10.1002/1873-3468.14404.

Guo, Z., Li, Y., and Ding, S.W. (2019). Small RNA-based antimicrobial immunity. Nat. Rev. Immunol. **19**:31–44. https://doi.org/10.1038/s41577-018-0071-x.

Guo, Z., Lu, J., Wang, X., Zhan, B., Li, W., and Ding, S.W. (2017). Lipid flippases promote antiviral silencing and the biogenesis of viral and host siRNAs in *Arabidopsis*. Proc. Natl. Acad. Sci. USA **114**:1377–1382. https://doi.org/10.1073/pnas.1614204114.

Guo, Z., Wang, X.B., Wang, Y., Li, W.X., Gal-On, A., and Ding, S.W. (2018). Identification of a new host factor required for antiviral RNAi and amplification of viral siRNAs. Plant Physiol. **176**:1587–1597. https://doi.org/10.1104/pp.17.01370.

Hannon, G.J. (2002). RNA interference. Nature **418**:244–251. https://doi.org/10.1038/418244a.

Jasieniecka-Gazarkiewicz, K., Demski, K., Lager, I., Stymne, S., and Banaś, A. (2016). Possible role of different yeast and plant lysophospholipid:Acyl-CoA acyltransferases (LPLATs) in Acyl remodelling of phospholipids. Lipids **51**:15–23. https://doi.org/10.1007/s11745-015-4102-0.

Jasieniecka-Gazarkiewicz, K., Lager, I., Carlsson, A.S., Gutbrod, K., Peisker, H., Dörmann, P., Stymne, S., and Banaś, A. (2017). Acyl-CoA: lysophosphatidylethanolamine acyltransferase activity regulates growth of *Arabidopsis*. Plant Physiol. **174**:986–998. https://doi.org/10.1104/pp.17.00391.

Jasieniecka-Gazarkiewicz, K., Demski, K., Gidda, S.K., Klińska, S., Niedojadło, J., Lager, I., Carlsson, A.S., Minina, E.A., Mullen, R.T., Bozhkov, P.V., et al. (2021). Subcellular localization of Acyl-CoA: lysophosphatidylethanolamine acyltransferases (LPEATs) and the effects of knocking-out and overexpression of their genes on autophagy markers level and life span of *A. thaliana*. Int. J. Mol. Sci. **22**:3006. https://doi.org/10.3390/ijms22063006.

Jin, Y., Zhao, J.H., and Guo, H.S. (2021). Recent advances in understanding plant antiviral RNAi and viral suppressors of RNAi. Curr. Opin. Virol. **46**:65–72. https://doi.org/10.1016/j.coviro.2020.12.001.

Jones, R.A.C. (2021). Global plant virus disease pandemics and epidemics. Plants-Basel **10**:233. https://doi.org/10.3390/plants10020233.

Kang, Y., Lin, W., and Nagy, P.D. (2024). Subversion of selective autophagy for the biogenesis of tombusvirus replication organelles inhibits autophagy. PLoS Pathog. **20**:e1012085. https://doi.org/10.1371/journal.ppat.1012085.

Li, F., Zhang, M., Zhang, C., and Zhou, X. (2020a). Nuclear autophagy degrades a geminivirus nuclear protein to restrict viral infection in solanaceous plants. New Phytol. **225**:1746–1761. https://doi.org/10.1111/nph.16268.

Li, F., Zhang, C., Tang, Z., Zhang, L., Dai, Z., Lyu, S., Li, Y., Hou, X., Bernards, M., and Wang, A. (2020b). A plant RNA virus activates selective autophagy in a UPR-dependent manner to promote virus infection. New Phytol. **228**:622–639. https://doi.org/10.1111/nph.16716.

Li, F., Li, X., Zhao, S., Pan, F., Li, Z., Hao, Y., He, J., Wang, A., Kormelink, R., and Zhou, X. (2025). Antiviral RNA interference in plants: Increasing complexity and integration with other biological processes. Plant Commun. **6**:101490. https://doi.org/10.1016/j.xplc.2025.101490.

Liu, S., Chen, M., Li, R., Li, W.X., Gal-On, A., Jia, Z., and Ding, S.W. (2022). Identification of positive and negative regulators of antiviral RNA interference in *Arabidopsis thaliana*. Nat. Commun. **13**:2994. https://doi.org/10.1038/s41467-022-30771-0.

Lopez-Gomollon, S., and Baulcombe, D.C. (2022). Roles of RNA silencing in viral and non-viral plant immunity and in the crosstalk between disease resistance systems. Nat. Rev. Mol. Cell Biol. **23**:645–662. https://doi.org/10.1038/s41580-022-00496-5.

Marshall, R.S., and Vierstra, R.D. (2018). Autophagy: the master of bulk and selective recycling. Annu. Rev. Plant Biol. **69**:173–208. https://doi.org/10.1146/annurev-arplant-042817-040606.

Masclaux-Daubresse, C., Chen, Q., and Havé, M. (2017). Regulation of nutrient recycling via autophagy. Curr. Opin. Plant Biol. **39**:8–17. https://doi.org/10.1016/j.pbi.2017.05.001.

Mourrain, P., Béclin, C., Elmayan, T., Feuerbach, F., Godon, C., Morel, J.B., Jouette, D., Lacombe, A.M., Nikic, S., Picault, N., et al. (2000). *Arabidopsis* SGS2 and SGS3 genes are required for posttranscriptional gene silencing and natural virus resistance. Cell **101**:533–542. https://doi.org/10.1016/S0092-8674(00)80863-6.

Nakatogawa, H. (2020). Mechanisms governing autophagosome biogenesis. Nat. Rev. Mol. Cell Biol. **21**:439–458. https://doi.org/10.1038/s41580-020-0241-0.

Otsuru, M., Yu, Y., Mizoi, J., Kawamoto-Fujioka, M., Wang, J., Fujiki, Y., and Nishida, I. (2013). Mitochondrial phosphatidylethanolamine level modulates Cyt c oxidase activity to maintain respiration capacity in *Arabidopsis thaliana* rosette leaves. Plant Cell Physiol. **54**:1612–1619. https://doi.org/10.1093/pcp/pct104.

Rasmussen, N.L., Kournoutis, A., Lamark, T., and Johansen, T. (2022). NBR1: The archetypal selective autophagy receptor. J. Cell Biol. **221**:e202208092. https://doi.org/10.1083/jcb.202208092.

Scholthof, K.B.G., Adkins, S., Czosnek, H., Palukaitis, P., Jacquot, E., Hohn, T., Hohn, B., Saunders, K., Candresse, T., Ahlquist, P., et al. (2011). Top 10 plant viruses in molecular plant pathology. Mol. Plant Pathol. **12**:938–954. https://doi.org/10.1111/J.1364-3703.2011.00752.X.

Sharma, S., Prasad, A., and Prasad, M. (2024). Selective autophagy: the fulcrum of plant-virus interaction. Trends Plant Sci. **29**:4–6. https://doi.org/10.1016/j.tplants.2023.10.003.

Stålberg, K., Ståhl, U., Stymne, S., and Ohlrogge, J. (2009). Characterization of two *Arabidopsis thaliana* acyltransferases with preference for lysophosphatidylethanolamine. BMC Plant Biol. **9**:60. https://doi.org/10.1186/1471-2229-9-60.

Tong, X., Liu, S.Y., Zou, J.Z., Zhao, J.J., Zhu, F.F., Chai, L.X., Wang, Y., Han, C., and Wang, X.B. (2021). A small peptide inhibits siRNA amplification in plants by mediating autophagic degradation of SGS3/RDR6 bodies. EMBO J. **40**:e108050. https://doi.org/10.15252/embj.2021108050.

Tong, X., Zhao, J.J., Feng, Y.L., Zou, J.Z., Ye, J., Liu, J., Han, C., Li, D., and Wang, X.B. (2023). A selective autophagy receptor VISP1 induces symptom recovery by targeting viral silencing suppressors. Nat. Commun. **14**:3852. https://doi.org/10.1038/s41467-023-39426-0.

van der Veen, J.N., Kennelly, J.P., Wan, S., Vance, J.E., Vance, D.E., and Jacobs, R.L. (2017). The critical role of phosphatidylcholine and phosphatidylethanolamine metabolism in health and disease. Biochim. Biophys. Acta. Biomembr. **1859**:1558–1572. https://doi.org/10.1016/j.bbamem.2017.04.006.

Vance, J.E., and Tasseva, G. (2013). Formation and function of phosphatidylserine and phosphatidylethanolamine in mammalian cells. Biochim. Biophys. Acta **1831**:543–554. https://doi.org/10.1016/j.bbalip.2012.08.016.

Völz, R., Park, J.Y., Harris, W., Hwang, S., and Lee, Y.H. (2021). Lysophosphatidylethanolamine primes the plant immune system and promotes basal resistance against hemibiotrophic pathogens. BMC Biotechnol. **21**:12. https://doi.org/10.1186/s12896-020-00661-8.

Wang, X.B., Wu, Q., Ito, T., Cillo, F., Li, W.X., Chen, X., Yu, J.L., and Ding, S.W. (2010). RNAi-mediated viral immunity requires amplification of virus-derived siRNAs in *Arabidopsis thaliana*. Proc. Natl. Acad. Sci. USA **107**:484–489. https://doi.org/10.1073/pnas.0904086107.

Wang, X.B., Jovel, J., Udomporn, P., Wang, Y., Wu, Q., Li, W.X., Gasciolli, V., Vaucheret, H., and Ding, S.W. (2011). The 21-nucleotide, but not 22-nucleotide, viral secondary small interfering RNAs direct potent antiviral defense by two cooperative Argonautes in *Arabidopsis thaliana*. Plant Cell **23**:1625–1638. https://doi.org/10.1105/tpc.110.082305.

Watt, L.G., Crawshaw, S., Rhee, S.J., Murphy, A.M., Canto, T., and Carr, J.P. (2020). The cucumber mosaic virus 1a protein regulates interactions between the 2b protein and ARGONAUTE 1 while maintaining the silencing suppressor activity of the 2b protein. PLoS Pathog. **16**:e1009125. https://doi.org/10.1371/journal.ppat.1009125.

Xie, Z., Johansen, L.K., Gustafson, A.M., Kasschau, K.D., Lellis, A.D., Zilberman, D., Jacobsen, S.E., and Carrington, J.C. (2004). Genetic and functional diversification of small RNA pathways in plants. PLoS Biol. **2**:E104. https://doi.org/10.1371/journal.pbio.0020104.

Yang, M., and Liu, Y. (2022). Autophagy in plant viral infection. FEBS Lett. **596**:2152–2162. https://doi.org/10.1002/1873-3468.14349.

Yang, M., and Liu, Y. (2022). Autophagy in plant viral infection. FEBS Lett. **596**:2152–2162. https://doi.org/10.1002/1873-3468.14349.

Yoshikawa, M., Han, Y.W., Fujii, H., Aizawa, S., Nishino, T., and Ishikawa, M. (2021). Cooperative recruitment of RDR6 by SGS3 and SDE5 during small interfering RNA amplification in *Arabidopsis*. Proc. Natl. Acad. Sci. USA **118**:e2102885118. https://doi.org/10.1073/pnas.2102885118.

Yu, L., Chen, Y., and Tooze, S.A. (2018). Autophagy pathway: cellular and molecular mechanisms. Autophagy **14**:207–215. https://doi.org/10.1080/15548627.2017.1378838.

Yu, L., Zhou, C., Fan, J., Shanklin, J., and Xu, C. (2021). Mechanisms and functions of membrane lipid remodeling in plants. Plant J. **107**:37–53. https://doi.org/10.1111/tpj.15273.

Zhuang, X., Li, B., and Jiang, L. (2024). Autophagosome biogenesis and organelle homeostasis in plant cells. Plant Cell **36**:3009–3024. https://doi.org/10.1093/plcell/koae099.

Plant Communications, Volume 7

Supplemental information

Antiviral RNAi defective 4 (AVI4) encodes a lysophospholipid acyltransferase coopting with AVI2 and ATG8a to promote antiviral immunity in *Arabidopsis*

Wenkai Yan, Yaogui Zhou, Bei Jia, Jiangshuai Niu, Shihuai Jin, Zhuoning Zhao, and Zhongxin Guo

Supplemental Figures and Table:

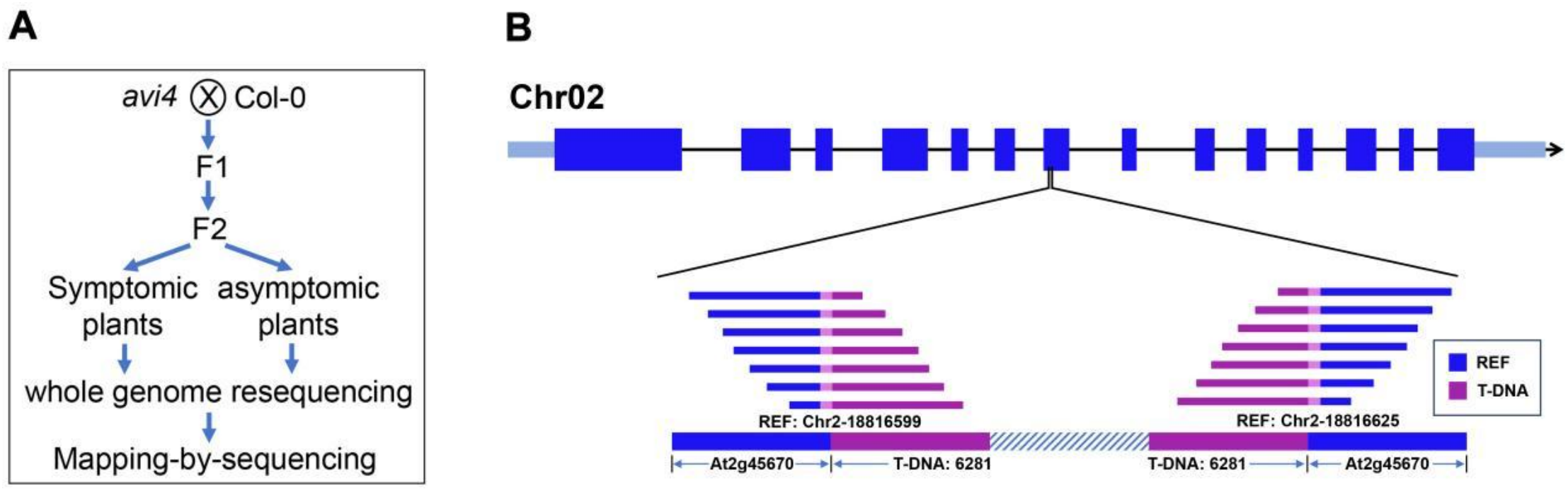

Figure S1. Identification of *avi4* T-DNA position. (A) The flowchart for identifying the causal mutation in *avi4* by whole-genome resequencing. (B) Schematic diagram showing reads flanking T-DNA borders and the position of T-DNA insertion on Chromosome 2 in *avi4* mutant.

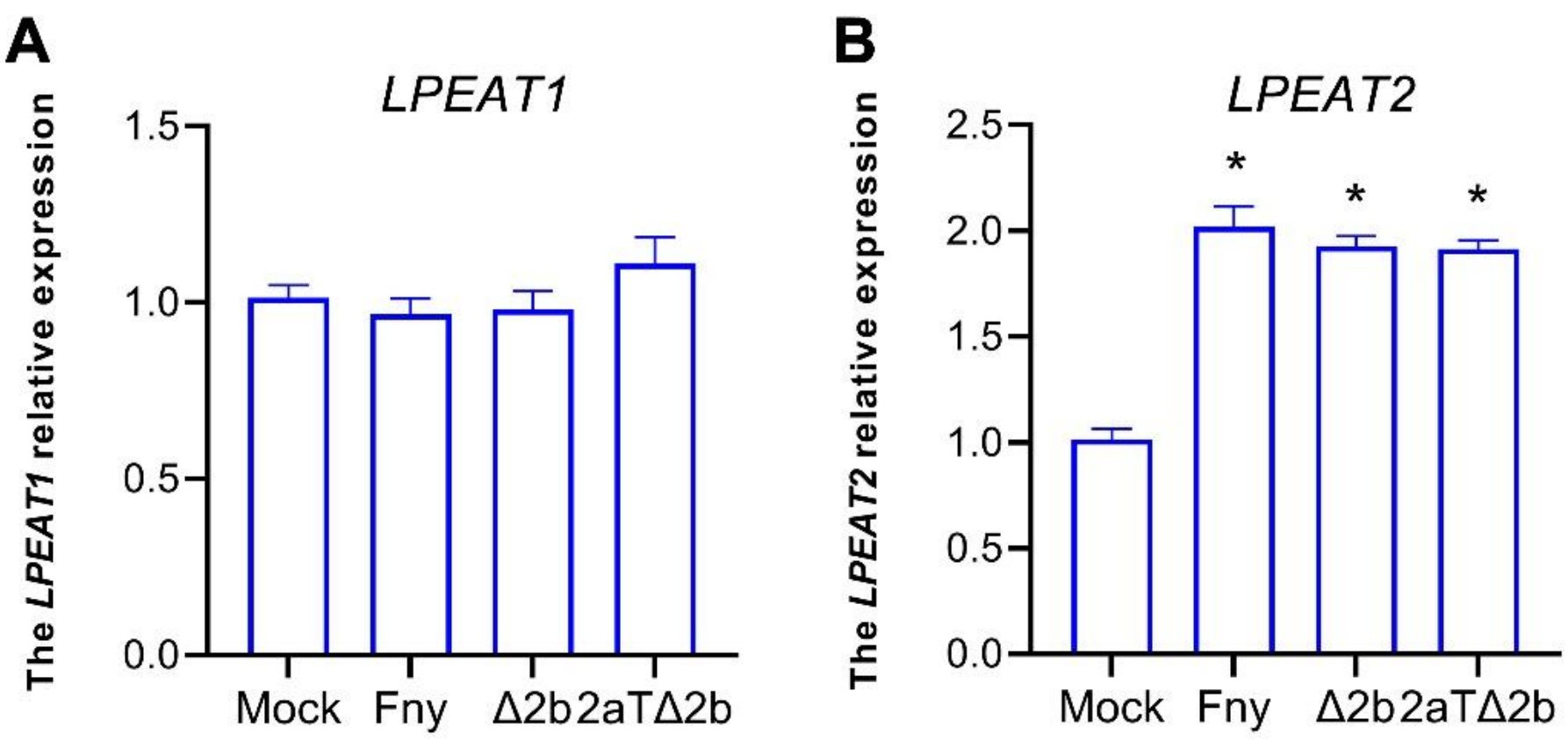

Figure S2. The expression of LPEAT2 is up-regulated in response to CMV infection. (A) and (B) Quantitative analysis of *LPEAT1* (A) and *LPEAT2* (B) mRNA accumulation in Arabidopsis, with or without viral infection. Col-0 plants were inoculated with buffer for mock treatment, or Fny-CMV, CMV-Δ2b, CMV-2aTΔ2b. Total RNA was extracted from systemic leaves 14 days post-infection, and the relative expression levels of the *LPEAT1* and *LPEAT2* genes were determined using RT-qPCR. *A. thaliana PP2A* mRNA served as an internal control. Error bars represent the standard deviation (SD) from three independent experiments, *$p < 0.05$ (Student's t-test).

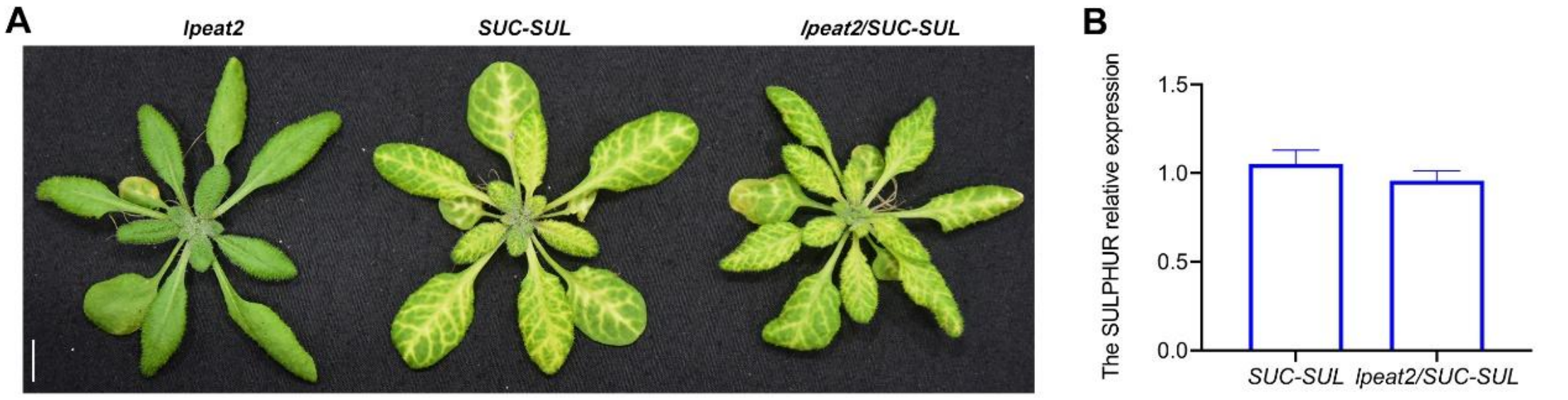

Figure S3. The silencing of *SULPHUR* (*SUL*) in SUC-SUL plants is not affected by LPAET2 mutation. (A) phenotype of *lpeat2*, *SUC-SUL* and *lpeat2 SUC-SUL* plants photographed approximately one month after germination. *SUC-SUL* and *lpeat2 SUC-SUL* showed the similar yellow vein phenotype caused by *SULPHUR* silencing. Bar= 1 cm. (B) RT-qPCR analysis showed that the relative expression level of *SULPHUR* gene in *SUC-SUL* and *lpeat2 SUC-SUL* plants did not show clear difference, *PP2A* was used as internal control, Error bars indicate standard deviation (SD).

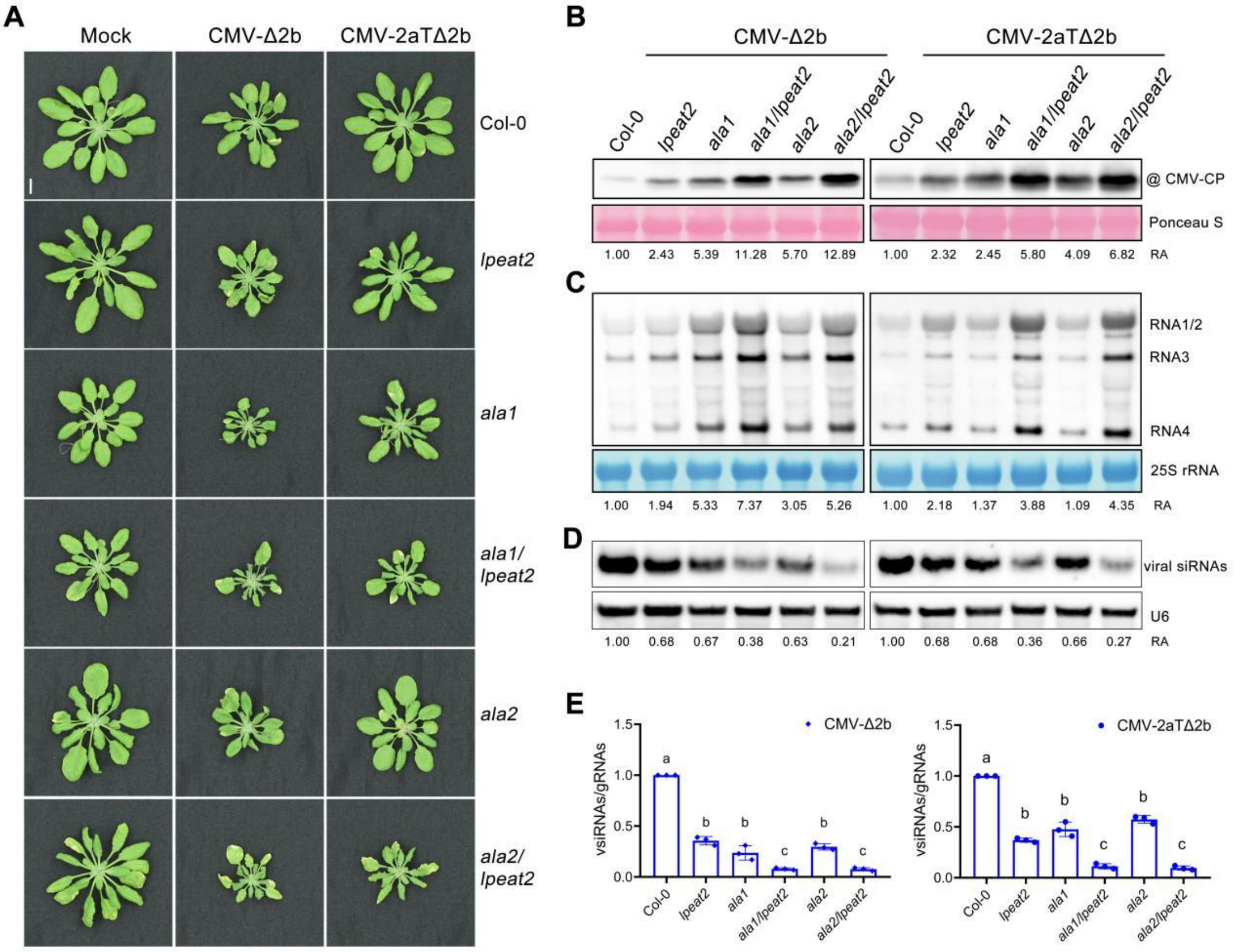

Figure S4. LPEAT2 functions independent of ALA1 and ALA2. (A) The phenotypes of plants photographed 14 days after inoculation with CMV-Δ2b, or CMV-2aTΔ2b or mock treatment with buffer, Bar= 1 cm. (B) Western blot analyses to detect the accumulation of virus coat protein in different plants, Rubisco in same membrane stained with ponceau staining to show equal loading. Relative accumulation (RA) of coat protein was calculated from Western blot signals using Image J. (C) and (D) Northern blot analyses of viral genomic RNAs or vsiRNAs accumulation in systemic leaves. 25S rRNA stained or U6

RNA probed as loading control, Relative accumulation (RA) of viral genomic RNAs or vsiRNAs was quantified using Image J by normalizing to 25S rRNA or U6, respectively. (E) Ratios of vsiRNAs/genomic RNAs were calculated from Northern hybridization signals in (C) and (D), with the ratio in Col-0 normalized to 1. The experiments were repeated three times independently with similar results. Data presented are means ± SEM from three replicates, letters indicate groups with significant differences (one-way ANOVA, Duncan, $p<0.05$) and dots represent the individual values.

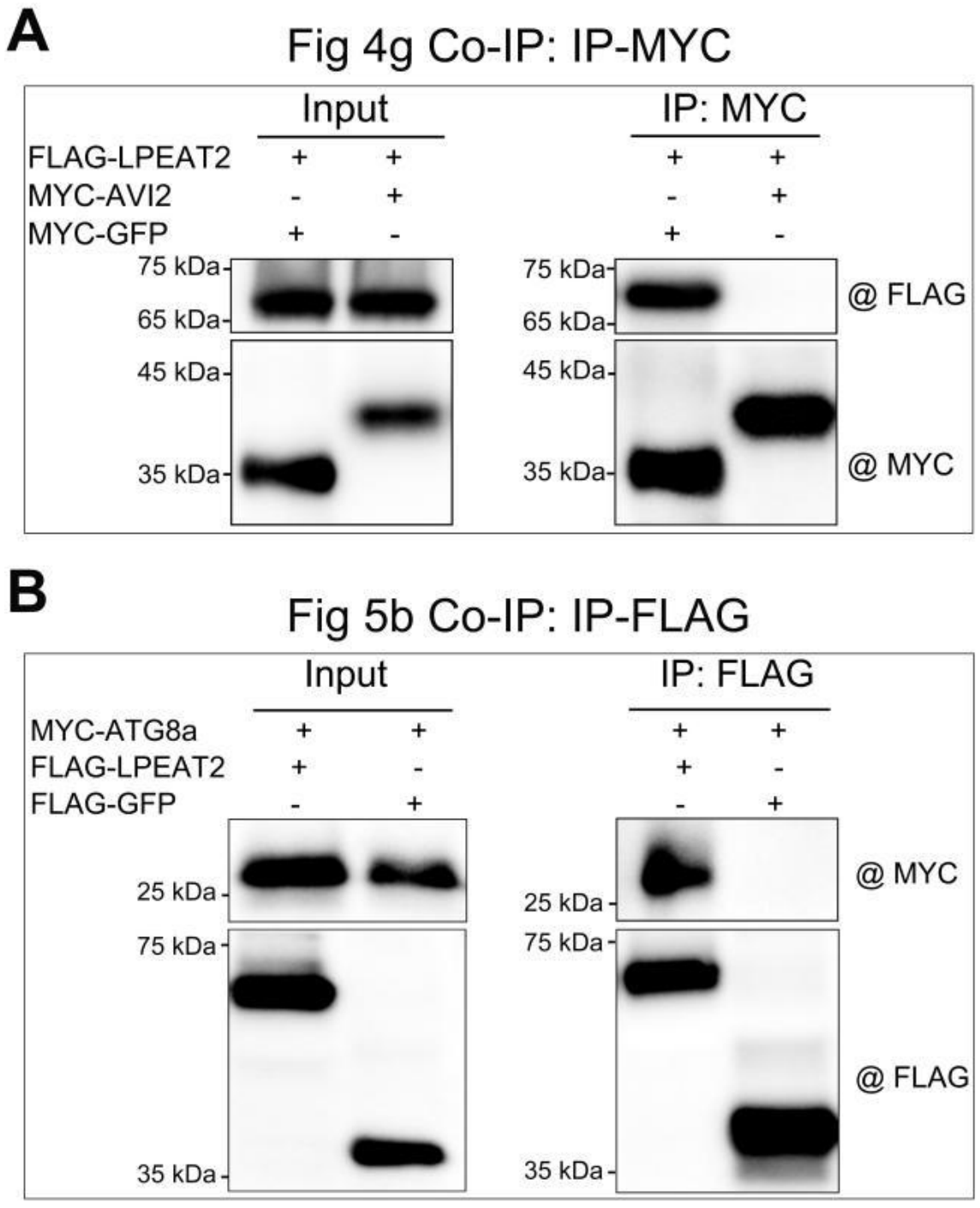

Fig. S5 Additional negative controls and experimental combinations for co-immunoprecipitation (Co-IP) assays corresponding to Fig. 4G and Fig. 5B. (A) Co-IP assay to assess the interaction between LPEAT2 and AVI2 proteins. (B) Co-IP assay to assess the interaction between LPEAT2 and ATG8a proteins.

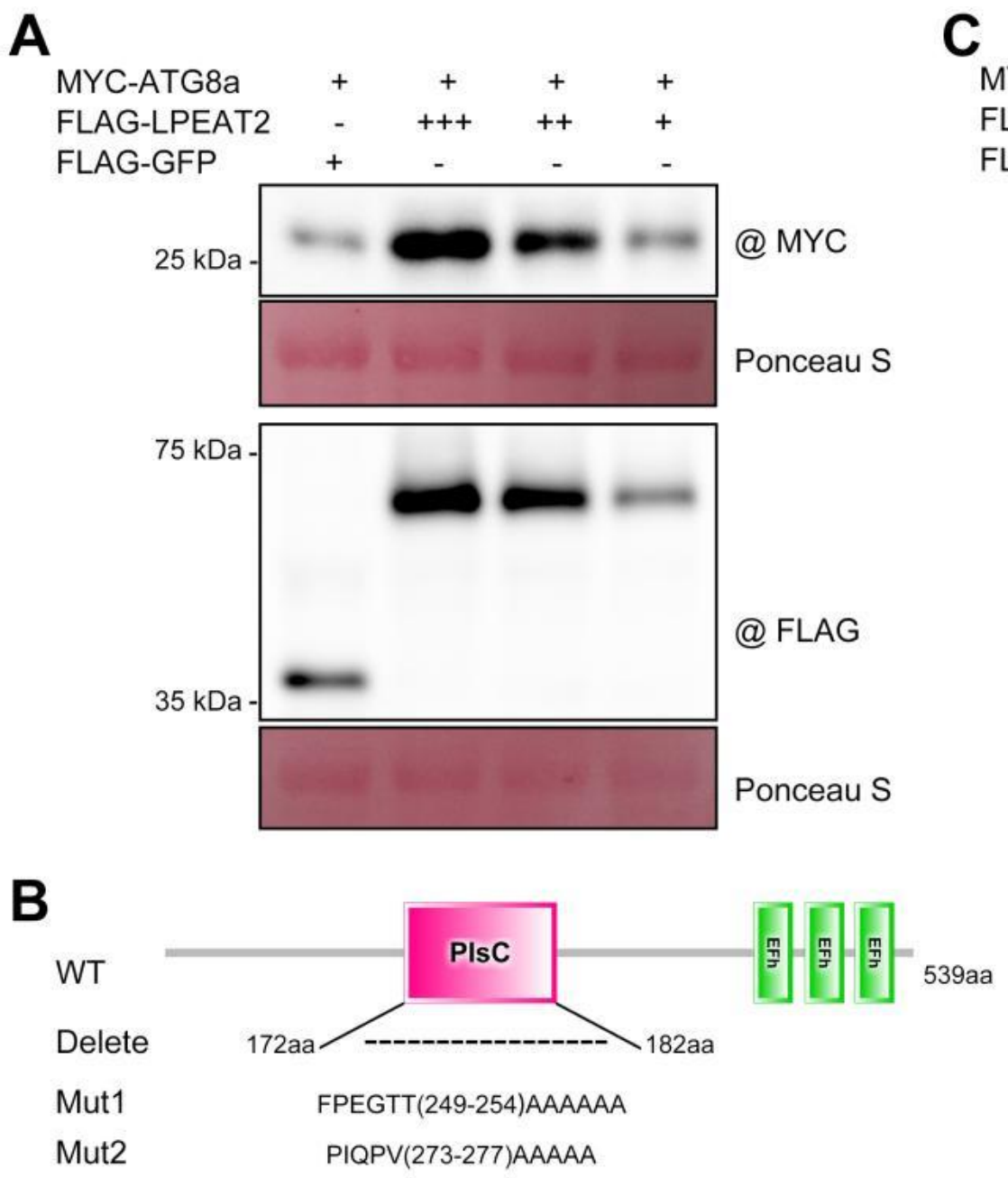

Fig. S6 The acyltransferase activity of LPEAT2 is essential for stabilizing the ATG8a protein. (A) AtLPEAT2 and AtATG8a were co-expressed in *N. benthamiana*, and protein accumulation levels were examined via Western blot assay using specific antibodies; Ponceau staining of Rubisco was employed as the loading control. (B) Schematic diagram of LPEAT2 acyltransferase activity-deficient mutants: the deletion mutation was generated by removing the 111-amino-acid (aa) PlsC domain, while the point mutations were constructed by substituting conserved amino acid residues with alanine. (C) Wild-type LPEAT2, LPEAT2 with deletion mutation or point mutation, and a GFP control were respectively co-expressed with ATG8a in *N. benthamiana*. The protein accumulation levels were detected by Western blot assay, with ponceau staining of Rubisco serving as the loading control.

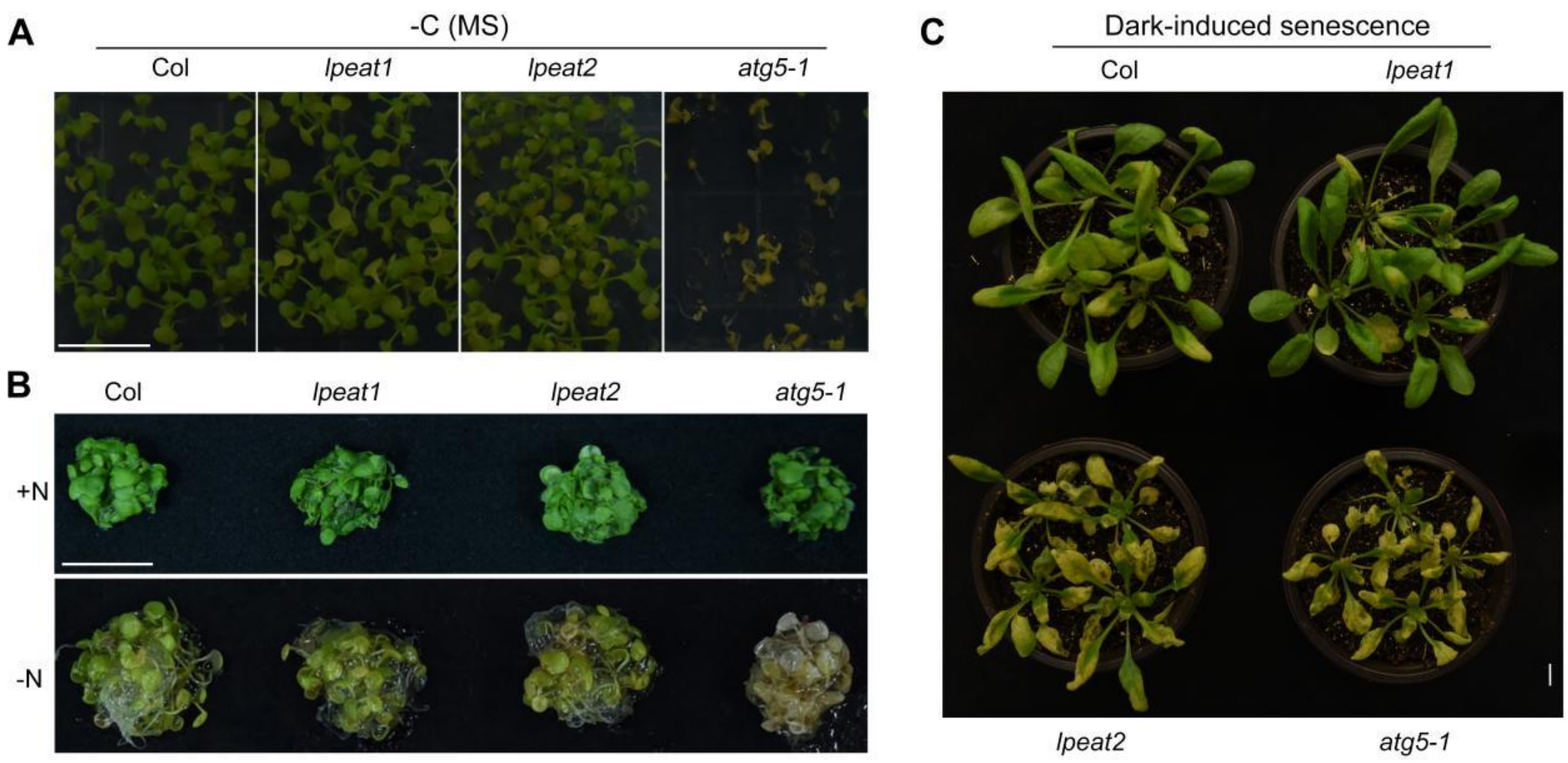

Figure S7. *lpeat2* mutants exhibit typical defects in autophagy under specific environmental stresses. (A) Plants were grown under long-day condition for 10 days in carbon-deficient MS (MS-C) medium plates, were then subjected to a 7-day dark period

treatment. Bar=1 cm. (B) Plants were grown under continuous light condition for 7 days on MS solid medium, were then transferred to Nitrogen-sufficient (+N) or Nitrogen-deficient (–N) MS liquid medium for an additional 7 days. Bar=1 cm. (C) enhanced darkness-induced senescence in *lpeat2*, *atg5-1*, compared to Col-0 and *lpeat1*. Plants were grown under long-day condition for approximately one month, were then subjected to a 7-day dark treatment, followed by a 5-7 day recovery period under long-day condition. Bar=1 cm.

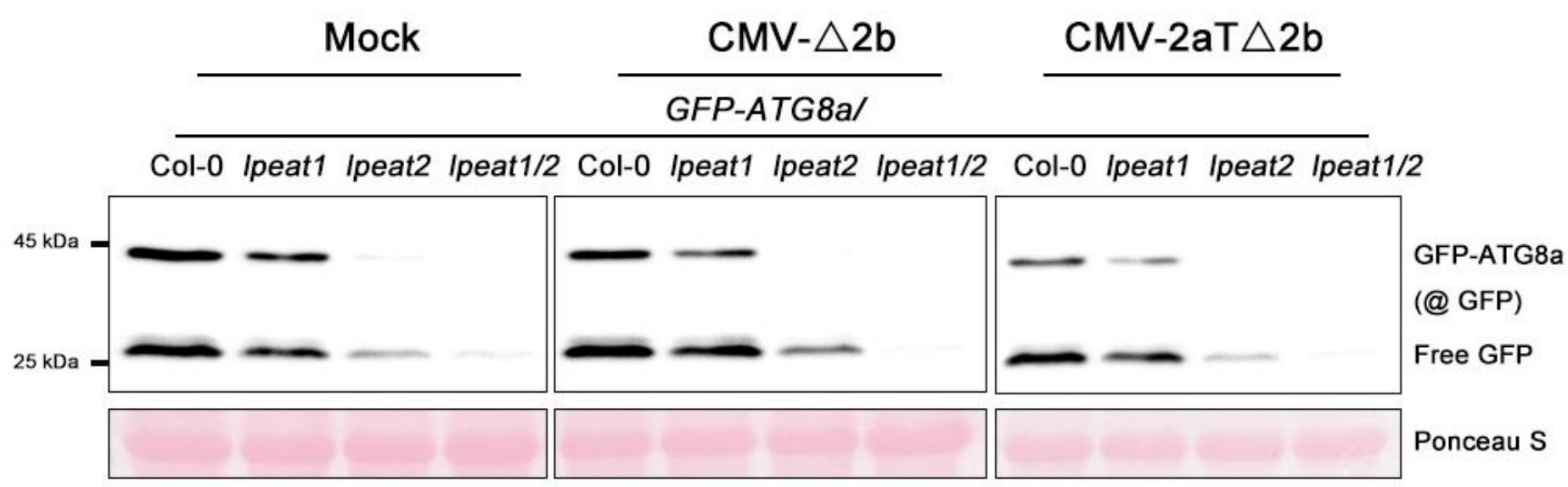

Figure S8. The accumulation of GFP-ATG8a protein in different crossed plants. Western blot analyses to detect the accumulation of GFP-ATG8a and free GFP proteins in plants after infection with CMV-Δ2b, CMV-2aTΔ2b, or mock treatment. Total proteins were probed with GFP antibodies, Rubisco in same membrane stained with ponceau staining to show equal loading.

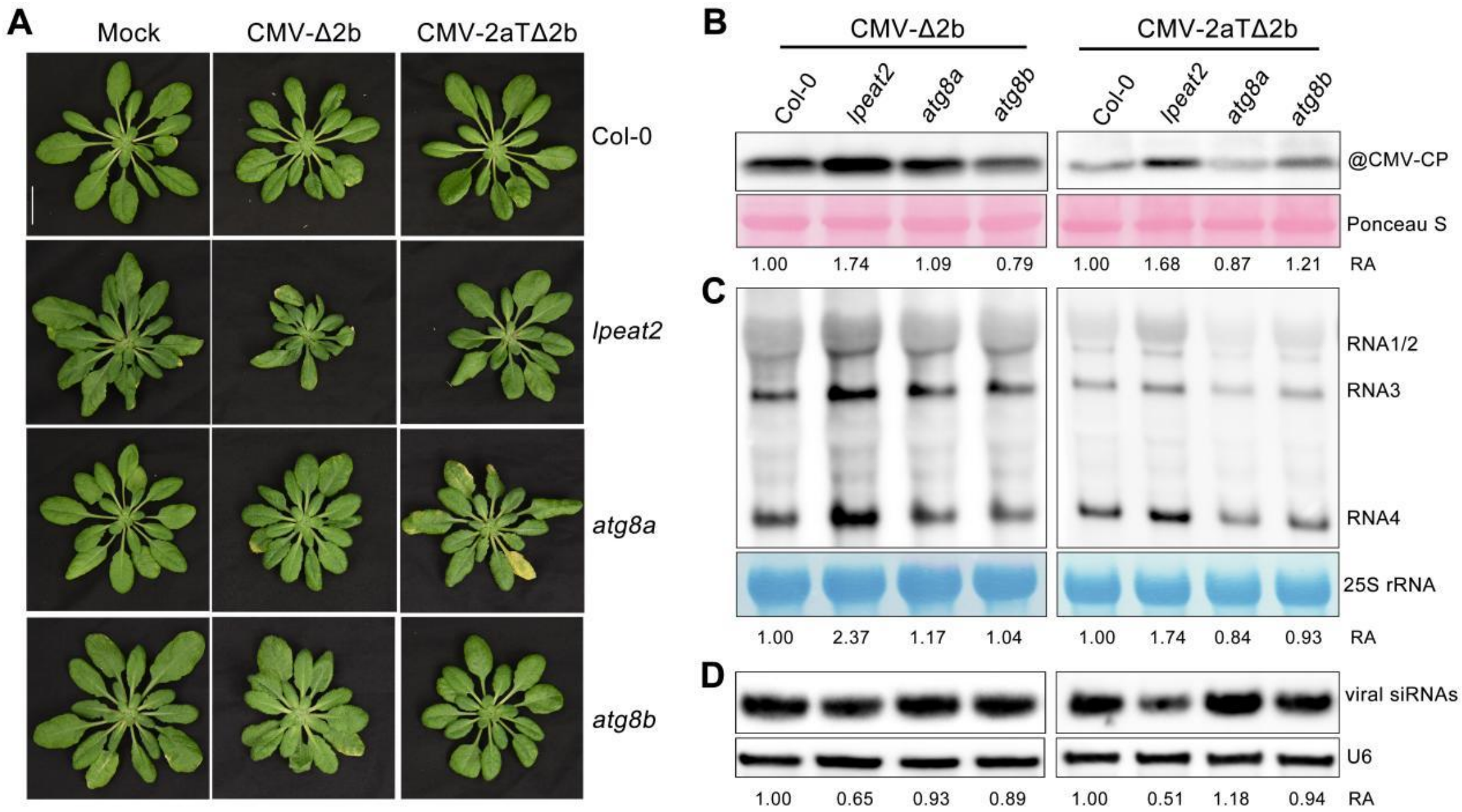

Figure S9. *atg8a* or *atg8b* single mutants did not show antiviral defects. (A) plants were photographed 2 weeks after infection with CMV-Δ2b, CMV-2aTΔ2b, or mock treatment, Bar= 1 cm. (B) Western blot analyses to detect the accumulation of virus coat protein in plants, Rubisco in same membrane stained with ponceau staining to show equal loading. Relative accumulation (RA) of coat protein was calculated from Western blot signals using Image J. (C) and (D) Northern blot analyses of viral genomic RNAs or

vsiRNAs accumulation in systemic leaves. 25S rRNA stained or U6 RNA probed as loading control, Relative accumulation (RA) of viral genomic RNAs or vsiRNAs was quantified using Image J by normalizing to 25S rRNA or U6, respectively.

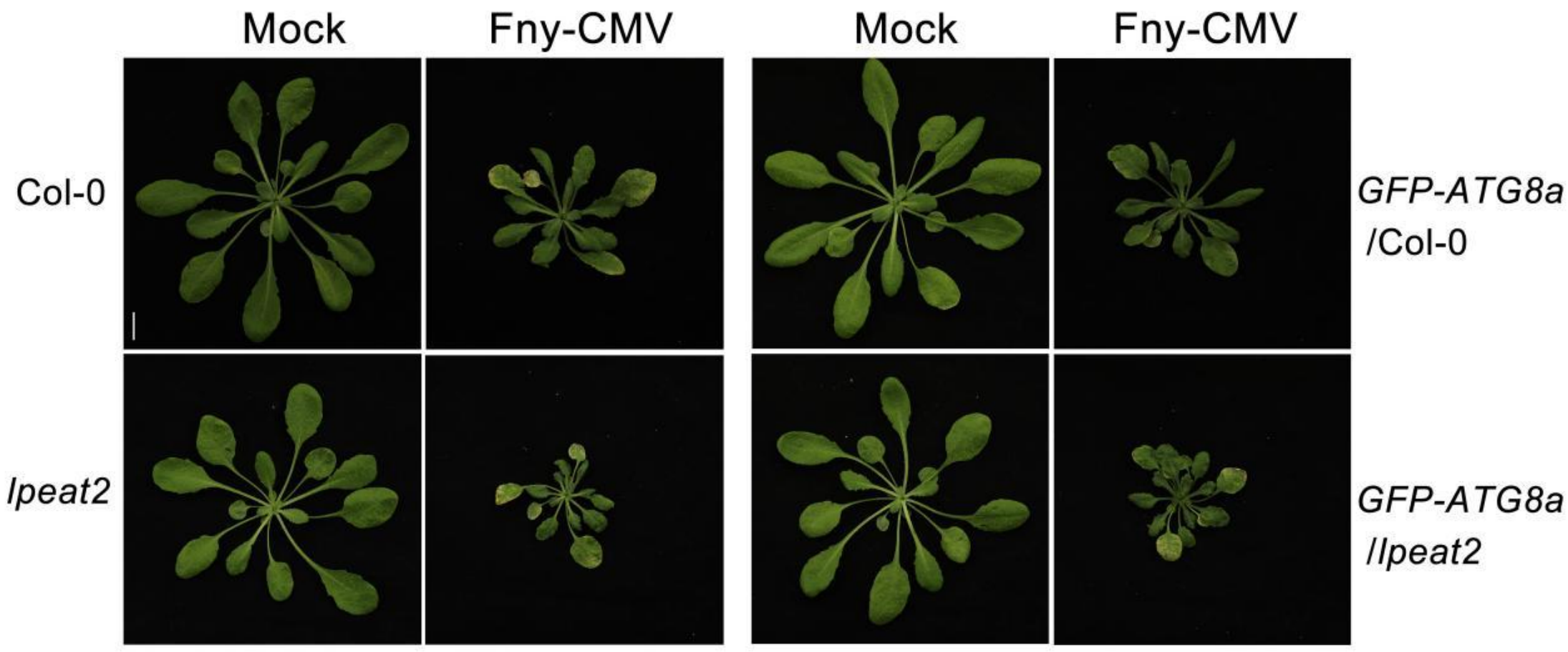

Fig S10. Phenotype of plants infected with Fny-CMV. The plants photographed 14 days after inoculation with Fny-CMV or mock treatment, Bar= 1 cm.

Fig. 2E Repeat#2

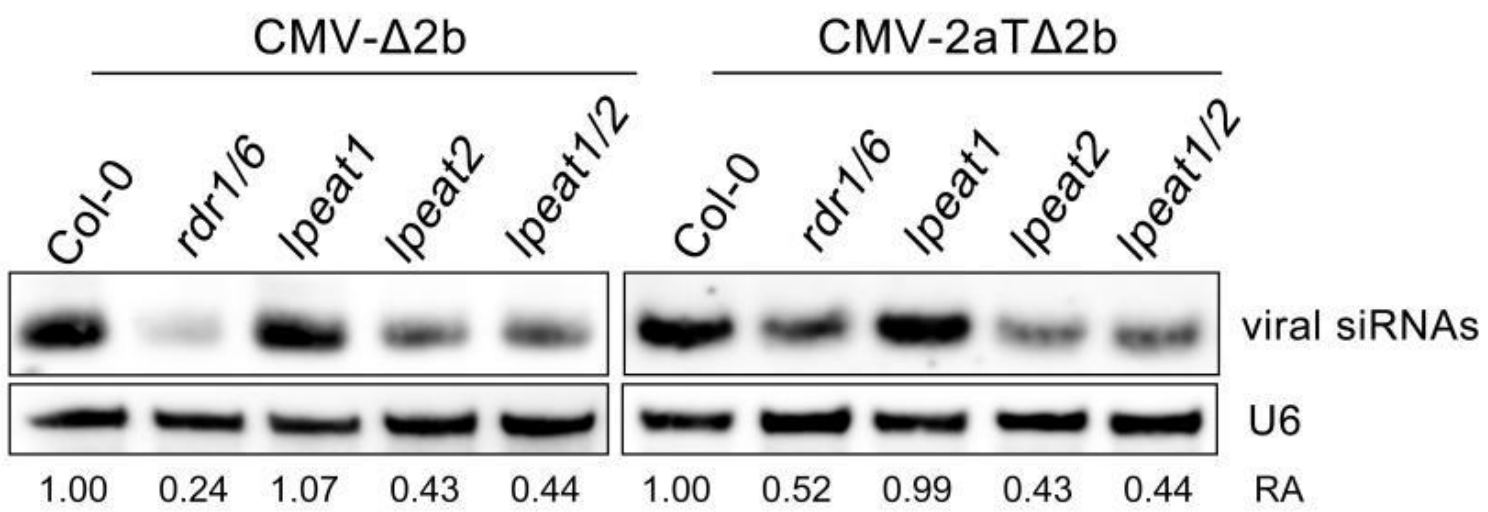

Fig. 2E Repeat#3

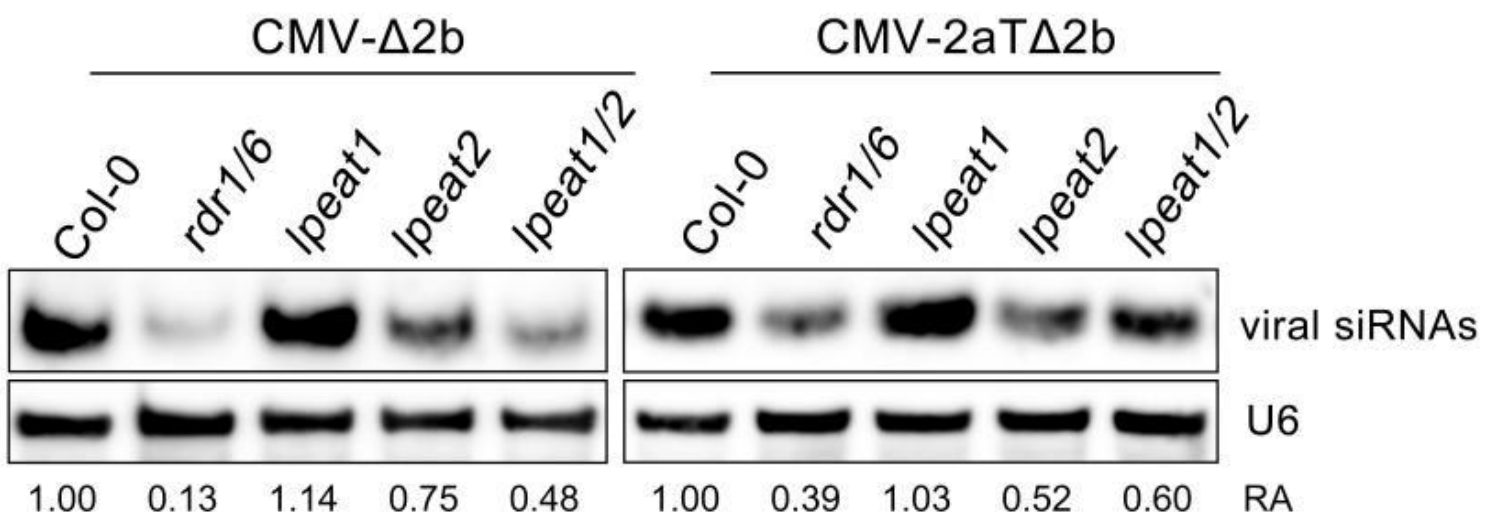

Fig. S11 Two additional biological replicates of viral siRNA detection via Northern blot assay, corresponding to Fig. 2E.

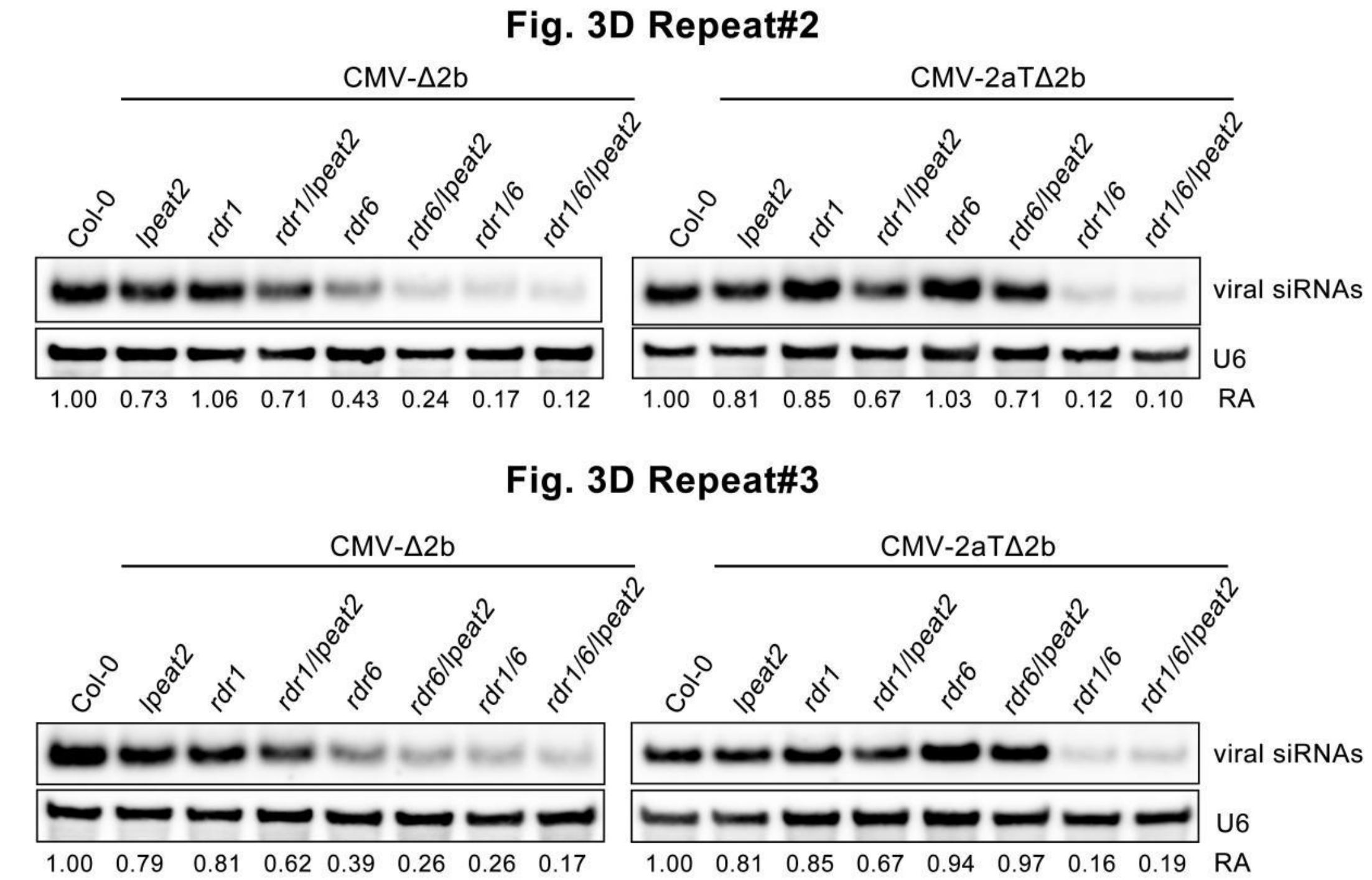

Fig. S12 Two additional biological replicates of viral siRNA detection via Northern blot assay, corresponding to Fig. 3D.

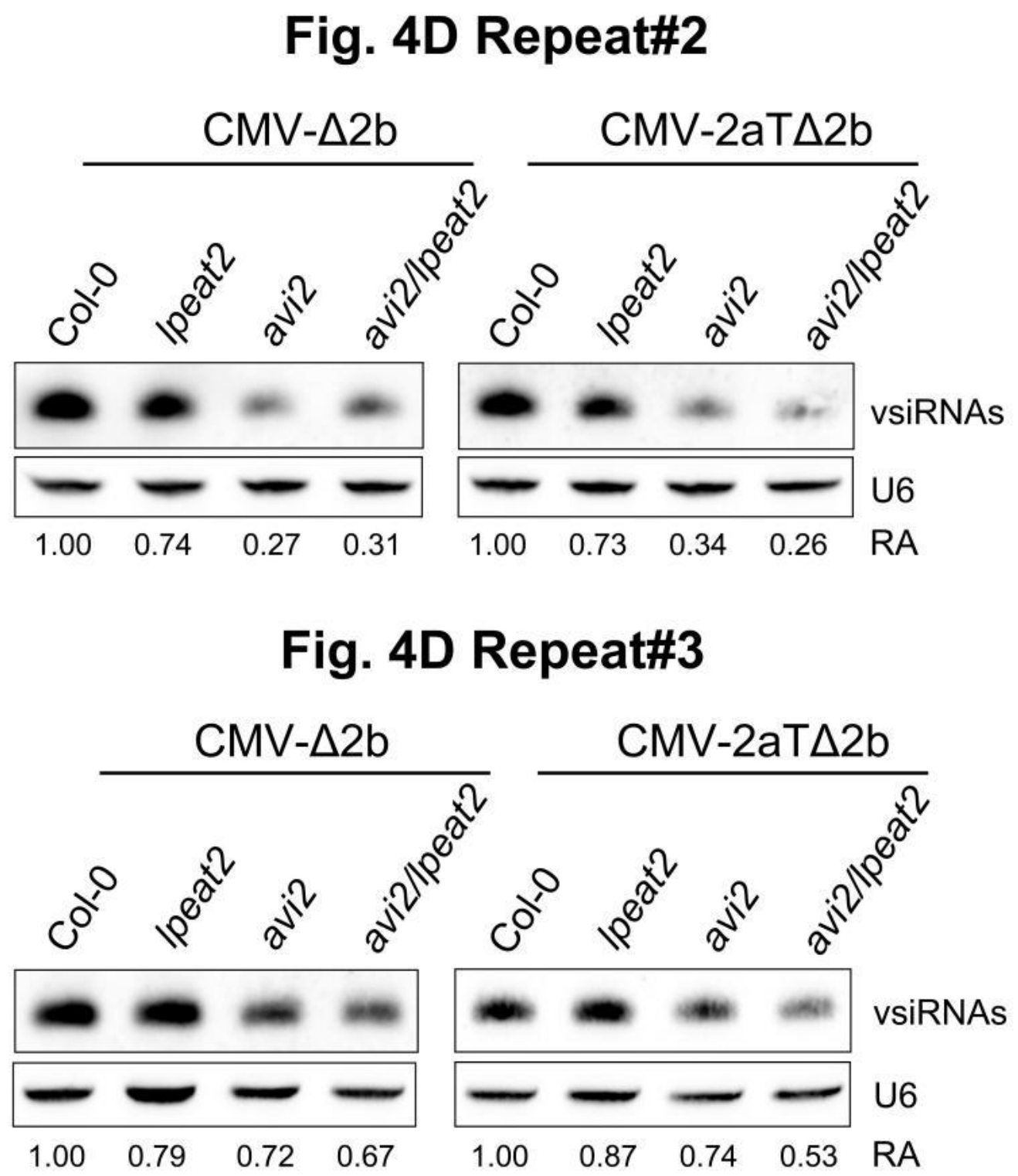

Fig. S13 Two additional biological replicates of viral siRNA detection via Northern blot assay, corresponding to Fig. 4D.

Fig 7D Repeat #2

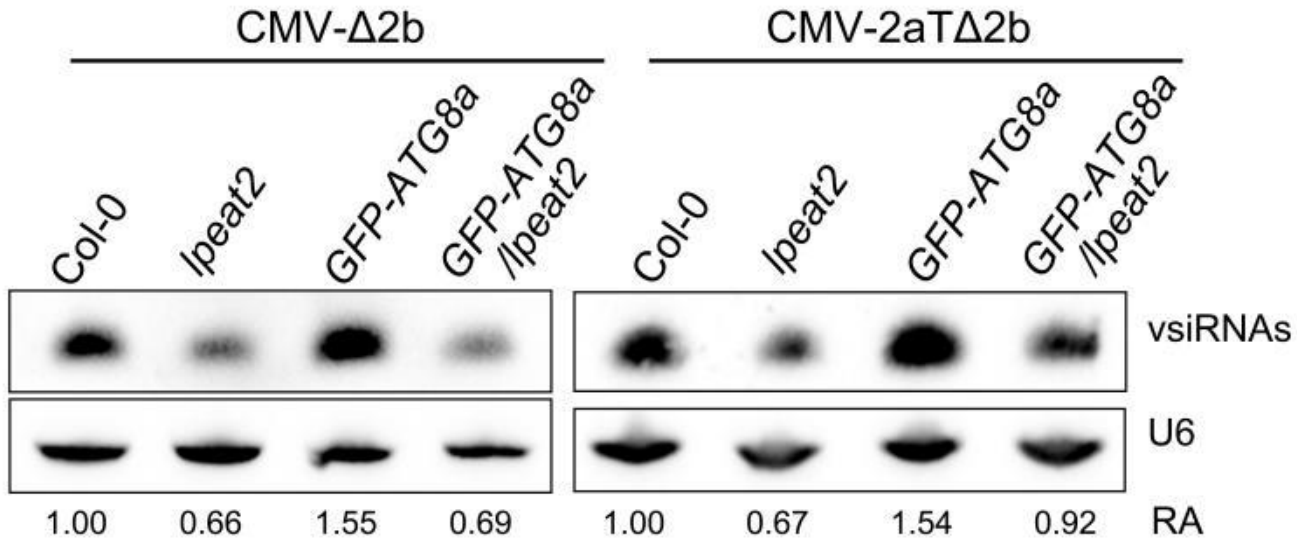

Fig 7D Repeat #3

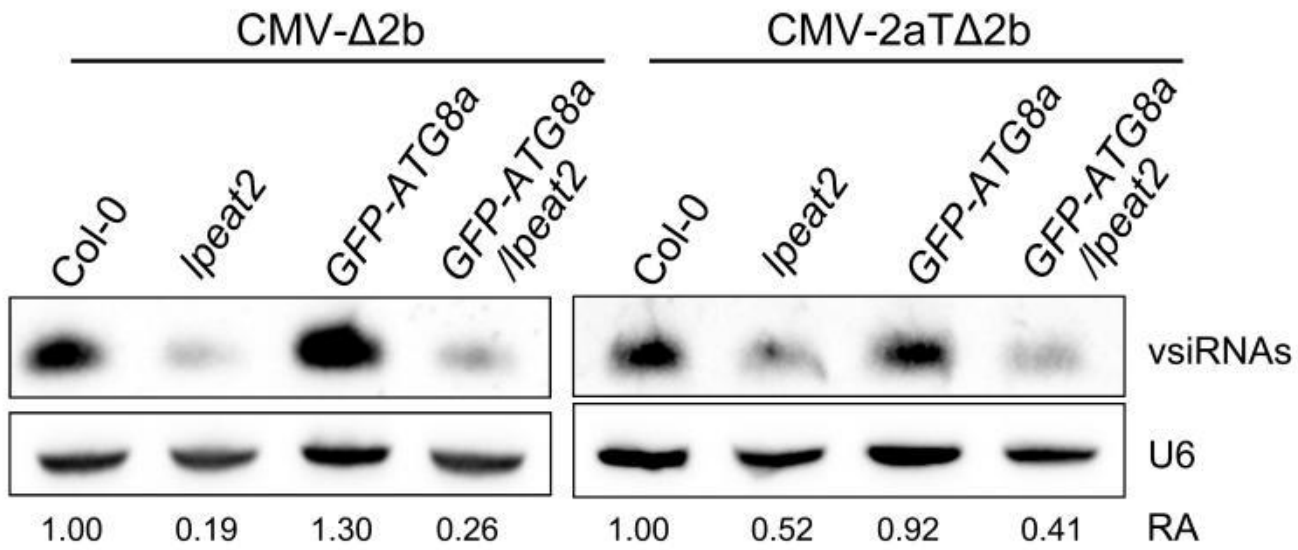

Fig. S14 Two additional biological replicates of viral siRNA detection via Northern blot assay, corresponding to Fig. 7D.

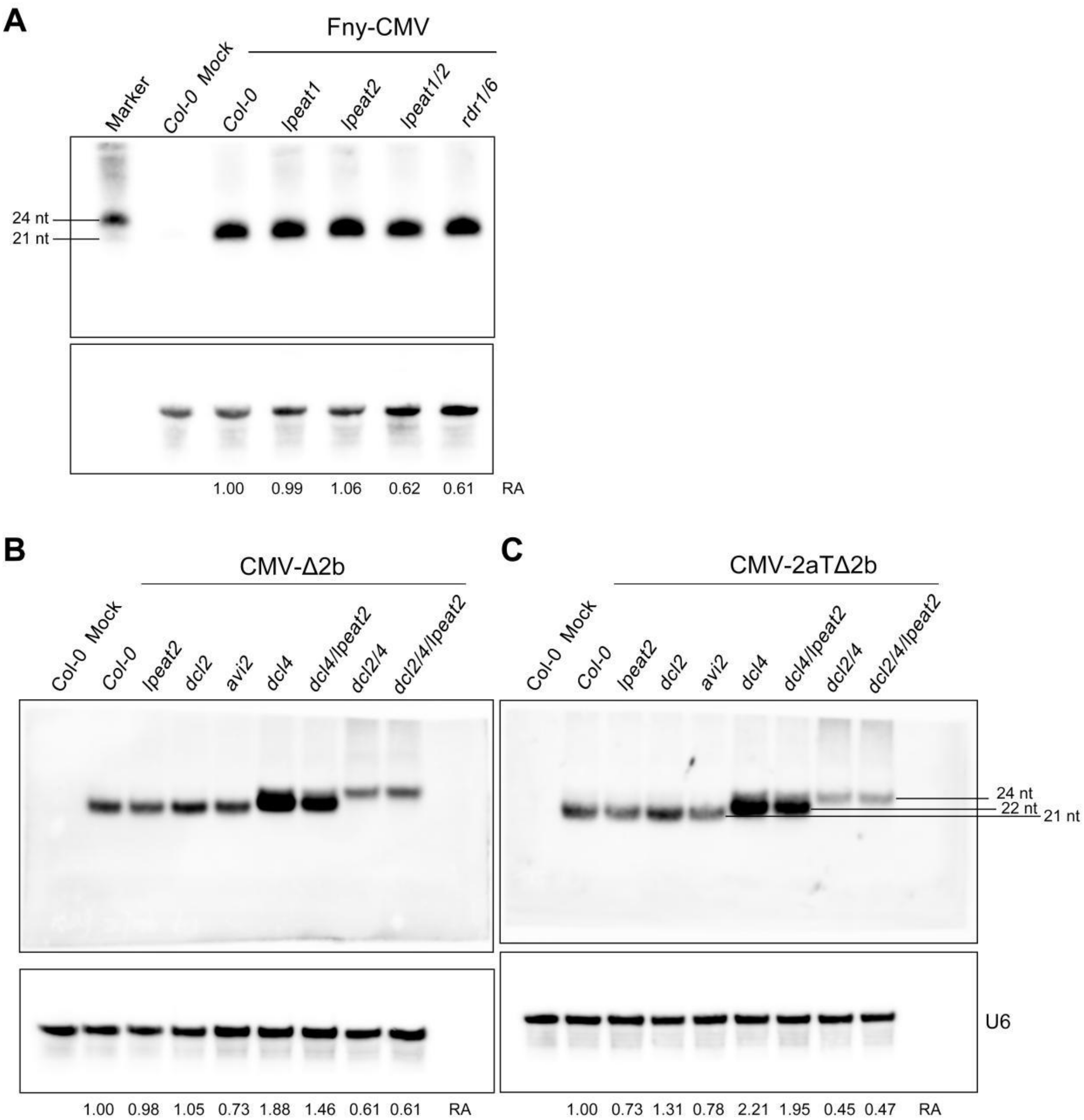

Fig. S15 Northern blot analyses of vsiRNAs produced in different Arabidopsis plants. (A) After wildtype Fny-CMV infection, 21 nt vsiRNAs were detected in Col-0, *lpeat1*, *lpeat2*, *lpeat1/2* and *rdr1/6* plants, the size of 21 nt vsiRNAs was indicated with small RNA marker. U6 RNA serving as the loading control. (B-C) after the infection of CMV-Δ2b (B) or CMV-2aT2b (C), 21 nt vsiRNAs were produced in Col-0, *lpeat2, dcl2, avi2*, compared to 22 nt viral siRNAs exclusively produced in *dcl4* and *dcl4 lpeat2* double mutants and 24 nt viral siRNAs only produced in *dcl2 dcl4* and *dcl2 dcl4 lpeat2* mutants. U6 RNA serving as the loading control.

Fig. 2E Original figures

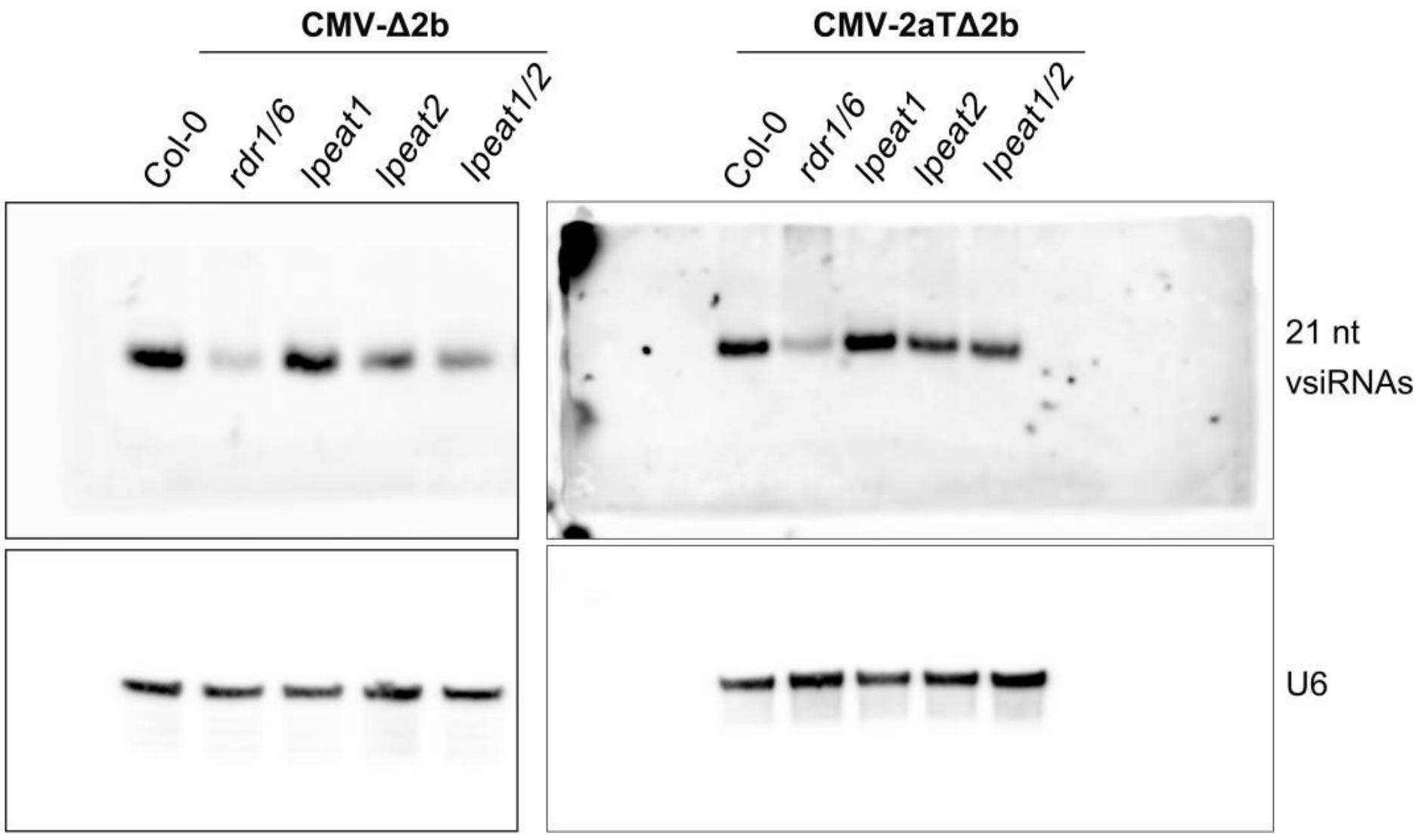

Fig. 3D Original figures

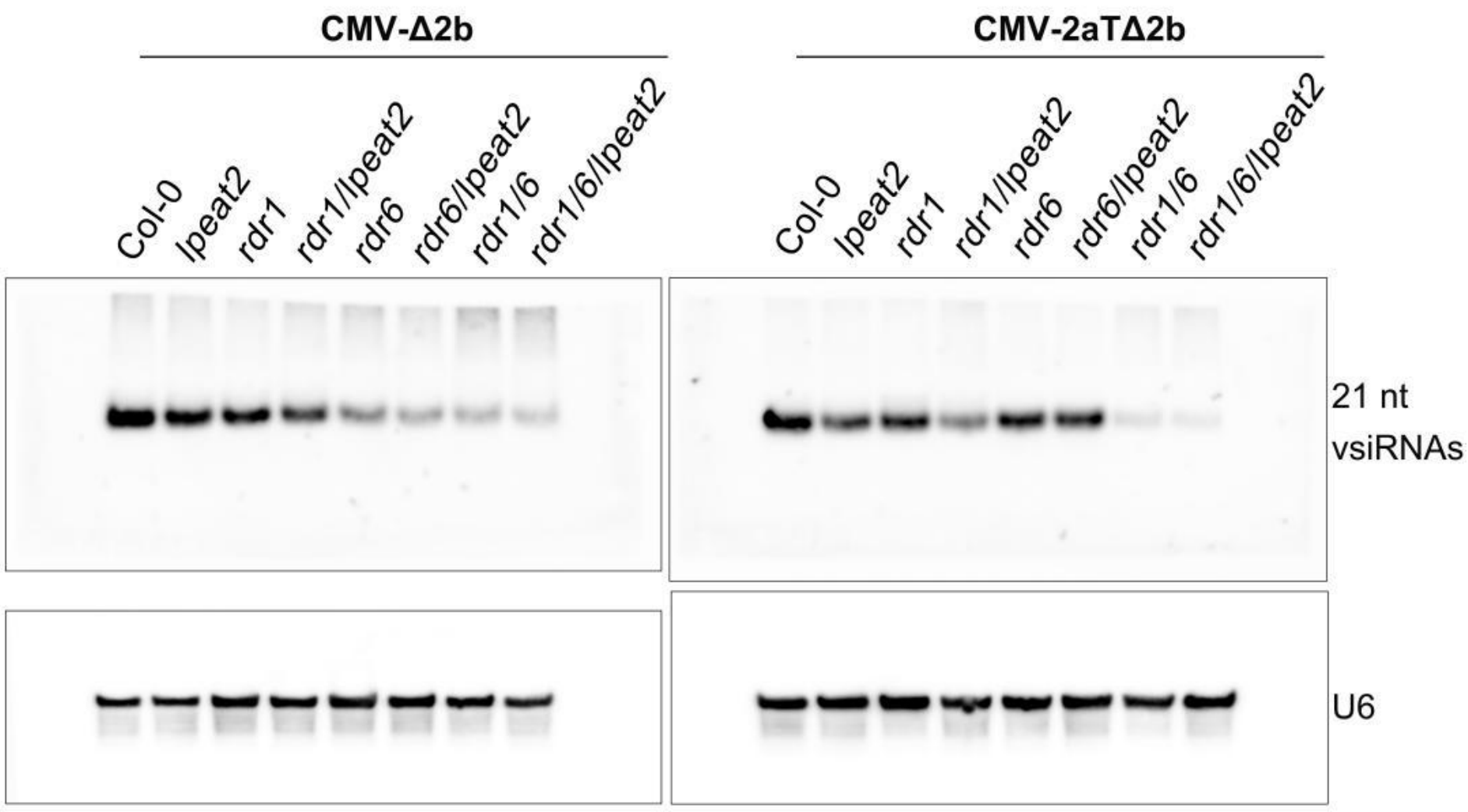

Fig. S16 The original images of Northern blot assay corresponding to Fig. 2E (up) or Fig. 3D (down), respectively.

Table S1. Primers used in the study

Name	5' to 3' Sequences
**Primers used for genotyping mutants by PCR**	
lpeat1 SALK_016798-LP	TTCAAAGCATGAGATAACCGG
lpeat1 SALK_016798-RP	AGTTTTGGGGCAATTTATTGG
lpeat2 SALK_073928-LP	TGGCTGATTCTTTGATCTTGG
lpeat2 SALK_073928-RP	ACATGGTTGAAATGGCAAGAG
lpeat2 SALK_107699-LP	ATGTATCCAAAAATCGCGTTG
lpeat2 SALK_107699-RP	AGCTTCCTGCGATAGATTTCC
atg8a SALK_045344-LP	TTTTCTGGGTTTGTTTCTCCC
atg8a SALK_045344-RP	TCATTAGCCCCAAAATCAATG
atg8b SALK_036033-LP	ACGAAAACGTTGAAAAACGAG
atg8b SALK_036033-RP	TTCTTCTCCATGATGATTCGG
LBb1.3	ATTTTGCCGATTTCGGAAC
**Primers used for quantitative RT-PCR**	
LPEAT1-qF	CTGTTTTCTGCTCCTTATCGTG
LPEAT1-qR	CGAAAAGCAAAACCCTAGAGAG
LPEAT2-qF	CGACGACCAAGATCAAAGAATC
LPEAT2-qR	GTGCAAAGATGGCTATGAGAAG
PP2A-qF	ATCGCTACTCAGTTTAACCACA
PP2A-qR	TAGCAATAGTTTGGGGCACTAA
SULFUR-qF	CTTCTGCAATCTGGGCTTCTC
SULFUR-qR	CAGTAGAGTTGATTTCAGTGGCT
**Primers used for *lpeat2* mutant complementation analysis**	
LPEAT2 native promoter-F	aaaagcaggctccgcGCATAATTCGAAACGTTTCG
LPEAT2 native promoter-R	catggtgaagggggcGTGGTGGAATGTTATTTGTTTC
pGWB10-npLPEAT2-F	caccATGGCGGATCCTGATCTGTCTTCTC
pGWB10-npLPEAT2-R	TTATGTTGGGGCCAAGTCAGGTGCA
**Primers used for gene overexpression**	
p1300-myc-LPEAT2-F	tctgaagaggacttgaatggtaccATGGCGGATCCTGATCTGTCTTCTC
p1300-myc-LPEAT2-R	tacgaacgaaagctctgcaggtcgacTTATGTTGGGGCCAAGTCAGGTGCA
**Primers used for Bimolecular Fluorescence Complementation (BiFC)**	
pDONR221-LPEAT2-F	ggggacaagtttgtacaaaaaagcaggcttcATGGCGGATCCTGATCTGTCTTCTC
pDONR221-LPEAT2-R	ggggaccactttgtacaagaaagctgggtcTGTTGGGGCCAAGTCAGGTGCAAAGA
pDONR221-ATG8a-F	ggggacaagtttgtacaaaaaagcaggcttcATGATCTTTGCTTGCTTGAAATTCGC
pDONR221-ATG8a-R	ggggaccactttgtacaagaaagctgggtcAGCAACGGTAAGAGATCCAAAAGTG
pDONR221-AVI2-F	ggggacaagtttgtacaaaaaagcaggcttcATGGAGACGGACAATGGGAAG
pDONR221-AVI2-R	ggggaccactttgtacaagaaagctgggtcGTATTCAGGACTGTACAGGC
pDONR221-GUS-F	ggggacaagtttgtacaaaaaagcaggcttcATGGTCCGTCCTGTAGAAACCCCAA
pDONR221-GUS-R	ggggaccactttgtacaagaaagctgggtcTTGTTTGCCTCCCTGCTGCGGTT
**Primers used for co-immunoprecipitation (Co-IP)**	
p1300-myc-AVI2-F	tctgaagaggacttgaatggtaccATGGAGACGGACAATGGGAAG

p1300-myc-AVI2-R	tacgaacgaaagctctgcaggtcgacCTAGTATTCAGGACTGTACAGGC
p1300-myc-ATG8a-F	tctgaagaggacttgaatggtaccATGATCTTTGCTTGCTTGAAATTCGC
p1300-myc-ATG8a-R	tacgaacgaaagctctgcaggtcgacTCAAGCAACGGTAAGAGATCCAAAAGTG
p1300-myc-GFP-F	tctgaagaggacttgaatggtacc
p1300-myc-GFP-R	tacgaacgaaagctctgcaggtcgac
p3301-flag-LPEAT2-F	gacgatgacaagacgcgtcccATGGCGGATCCTGATCTGTCTTCTC
p3301-flag-LPEAT2-R	attatggagaaagcttggatccTTATGTTGGGGCCAAGTCAGGTGCA
**Primers used for yeast two-hybrid (Y2H)**	
pGAD-ATG8a-F	gccatggaggccagtgaattcATGATCTTTGCTTGCTTGAAATTCGC
pGAD-ATG8a-R	agctcgagctcgatggatcccTCAAGCAACGGTAAGAGATCCAAAAGTG
pGAD-LPEAT2-F	gccatggaggccagtgaattcATGGCGGATCCTGATCTGTCTTCTC
pGAD-LPEAT2-R	agctcgagctcgatggatcccTTATGTTGGGGCCAAGTCAGGTGCA
pGAD-AVI2-F	gccatggaggccagtgaattcATGGAGACGGACAATGGGAAG
pGAD-AVI2-R	agctcgagctcgatggatcccCTAGTATTCAGGACTGTACAGGC
pGBK-ATG8a-F	atggccatggaggccgaattcATGATCTTTGCTTGCTTGAAATTCGC
pGBK-ATG8a-R	cgctgcaggtcgacggatcccTCAAGCAACGGTAAGAGATCCAAAAGTG
pGBK-LPEAT2-F	atggccatggaggccgaattcATGGCGGATCCTGATCTGTCTTCTC
pGBK-LPEAT2-R	cgctgcaggtcgacggatcccTTATGTTGGGGCCAAGTCAGGTGCA
pGBK-AVI2-F	atggccatggaggccgaattcATGGAGACGGACAATGGGAAG
pGBK-AVI2-R	cgctgcaggtcgacggatcccCTAGTATTCAGGACTGTACAGGC

F, forward; R, reverse.
